# Critical review of partial volume correction methods in PET and SPECT imaging: benefits, pitfalls, challenges, and future outlook

**DOI:** 10.1007/s00259-025-07612-5

**Published:** 2025-11-05

**Authors:** Mohammad Saber Azimi, Arman Rahmim, Hossein Arabi, Amirhossein Sanaat, Navid Zeraatkar, Yassine Bouchareb, Chi Liu, Abass Alavi, Michael King, Ronald Boellaard, Habib Zaidi

**Affiliations:** 1 Department of Medical Radiation Engineering, Shahid Beheshti University, Tehran, Iran; 2 Departments of Radiology and Physics, University of British Columbia, Vancouver, BC Canada; 3 Division of Nuclear Medicine & Molecular Imaging, Geneva University Hospital, Geneva, CH-1211 Switzerland; 4 Department of Radiology, University of Massachusetts Chan Medical School, Worcester, MA USA; 5 Department of Radiology & Molecular Imaging, College of Medicine and Health Sciences, Sultan Qaboos University, Muscat, 123 Oman; 6 Department of Radiology and Biomedical Imaging, Yale University, New Haven, CT USA; 7 Department of Radiology, Perelman School of Medicine, University of Pennsylvania, Philadelphia, PA USA; 8 Department of Radiology and Nuclear Medicine, Amsterdam UMC location Vrije Universiteit Amsterdam, Amsterdam, The Netherlands; 9 Department of Nuclear Medicine and Molecular Imaging, University of Groningen, University Medical Center Groningen, Groningen, Netherlands; 10 Department of Nuclear Medicine, University of Southern Denmark, Odense, Denmark; 11 University Research and Innovation Center, Óbuda University, Budapest, Hungary; 12 Division of Nuclear Medicine and Molecular Imaging, Geneva University Hospital, Geneva, CH-1211 Switzerland

**Keywords:** PET, SPECT, Partial volume correction (PVC), Image reconstruction, Anatomical information, Quantitative imaging

## Abstract

**Purpose:**

Partial volume effects (PVE) remain a major challenge in quantitative single-photon emission computed tomography (SPECT) and positron emission tomography (PET) imaging, often compromising both accuracy and reproducibility. While numerous Partial Volume Correction (PVC) methods have been proposed, their clinical translation is still limited. This review provides a clinically oriented evaluation of PVC methods with a particular focus on state-of-the-art applications in neurology, cardiovascular imaging, oncology, and radiopharmaceutical therapy dosimetry, highlighting where these techniques offer the greatest added value. In addition, we outline which PVC techniques have the potential to be used in clinical practice and which remain primarily suited for research purposes, along with their suitability in each of the above-mentioned clinical domains. Finally, this review addresses the central question of whether PVC is essential in clinical practice or whether its impact is context dependent.

**Methods:**

This review categorizes PVC approaches into three partially overlapping classes: reconstruction-based, post-reconstruction-based, and AI-driven or hybrid methods. Each class is further divided into anatomical and non-anatomical subcategories. We systematically compare their clinical applicability across key dimensions: quantitative accuracy, lesion detectability, robustness to noise and artifacts, anatomical dependence, generalizability across scanners and tracers, and clinical readiness.

**Results:**

PVC techniques often improve quantitative accuracy in small structures and in regions affected by spill-over from adjacent high-uptake tissues. However, these benefits can come at the cost of increased noise or edge artifacts, which may limit their robustness for routine clinical use. Post-reconstruction methods are sensitive to segmentation errors, while AI-driven models, despite their promise, require further validation using clinical benchmarks, comparison to ground truth, and testing on diverse datasets. Issues, such as generalizability and interpretability remain significant barriers.

**Conclusion:**

This review emphasizes the importance of application-tailored PVC protocols for reliable quantitative imaging in neurology, cardiology, oncology, and radiopharmaceutical therapy dosimetry. Not all PVC methods are beneficial; some may even impair interpretation in certain contexts. We provide a practical overview of which PVC approaches are most beneficial for each clinical scenario, aiming to guide both researchers and clinicians in selecting appropriate techniques for future studies and routine practice, and also outline key areas requiring further development for broader integration into research and clinical workflows.

**Graphical abstract:**

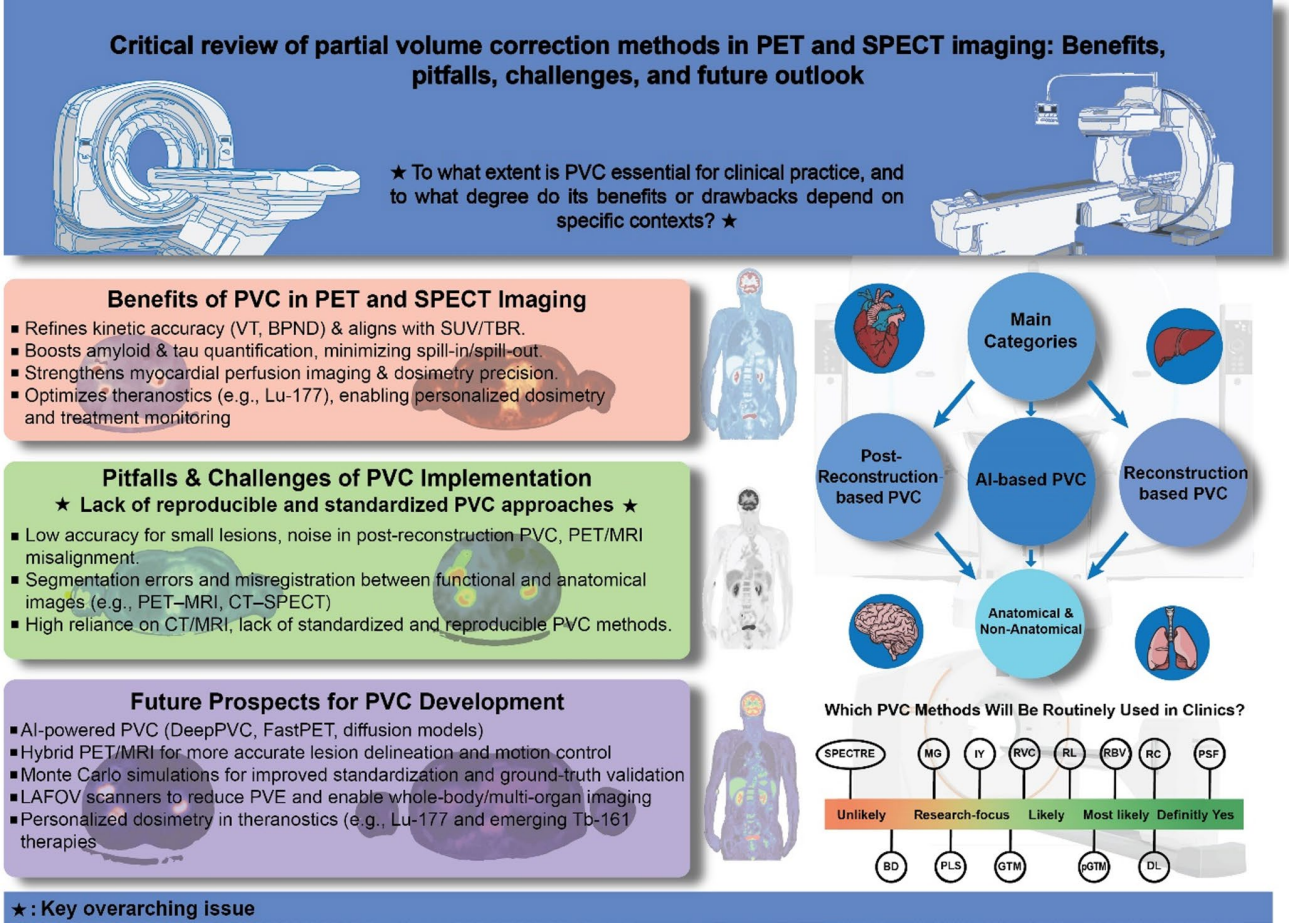

**Supplementary Information:**

The online version contains supplementary material available at 10.1007/s00259-025-07612-5.

## Introduction

**Fig. 1:**
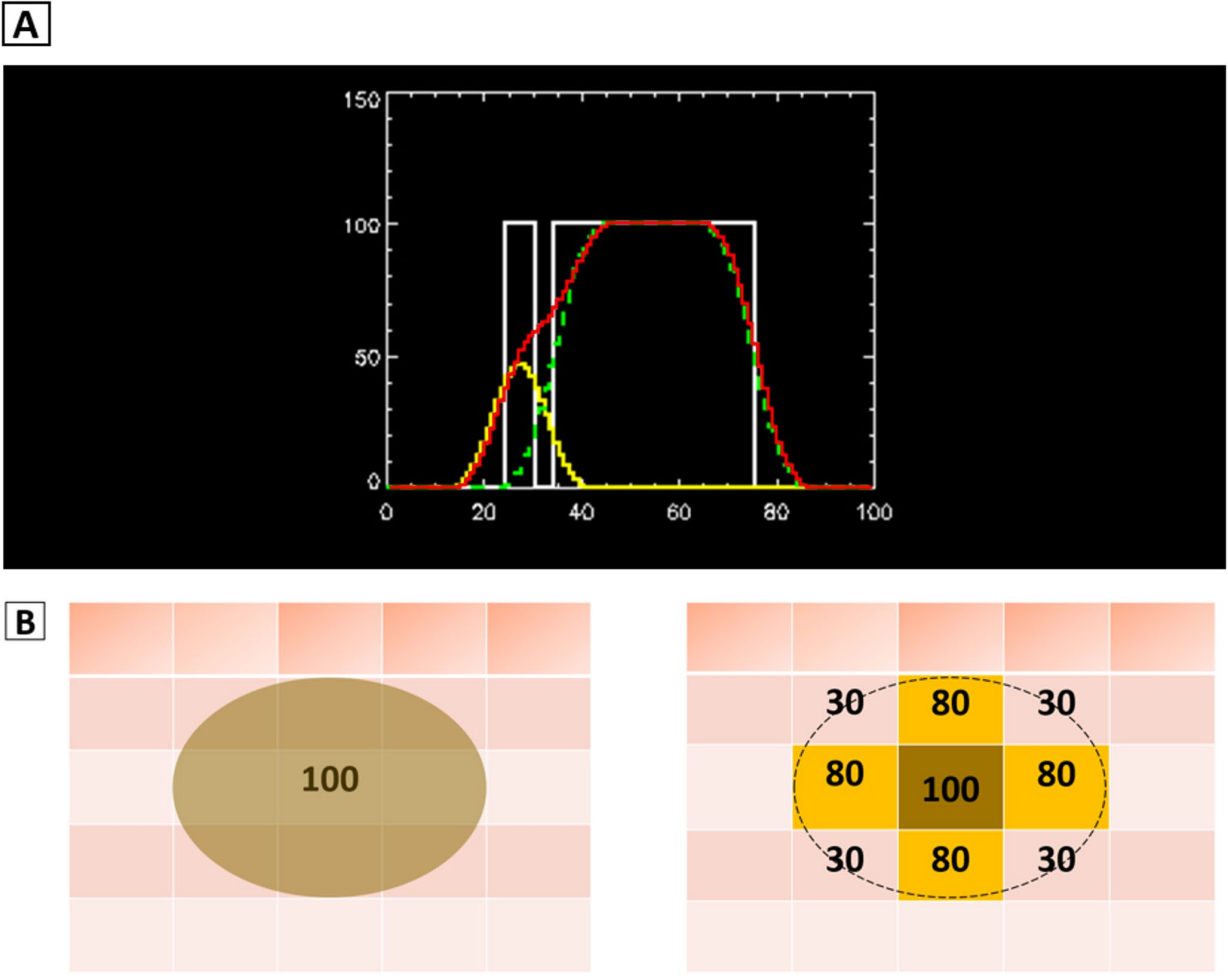
Depiction of the partial volume effect (PVE) in medical imaging. (**A**) A one-dimensional illustration of the PVE. The x-axis represents the position along a line profile through adjacent structures, and the y-axis represents the relative activity concentration. The white lines represent the relative concentration of activity in two structures with the same activity concentration but of different widths, separated by a narrow non-activity region. The yellow line represents a thin structure whose width is 2–3 times smaller than the full-width-at-half-maximum (FWHM) of the system, blurred by the spatial resolution of PET or SPECT imaging. Its maximum value is reduced to about half the true value, with activity spilling into adjacent regions (spill-out) and partly into the neighbouring thicker structure (spill-in). The green line represents a larger structure, where the centre activity is preserved but edges are underestimated due to spill-out. The red line shows the total activity profile (sum of thin and thick structures), illustrating how adjacent structures are blurred together. (**B**) Illustration of the tissue-fraction effect: tracer distribution sampled on a voxel grid where voxel boundaries do not align with the actual structure, leading to partial volume averaging

Positron emission tomography (PET) and single-photon emission computed tomography (SPECT) play crucial roles in functional imaging for a wide variety of clinical indications including neurology, cardiology, and oncology by visualizing metabolic and molecular processes in vivo [1–4]. However, their quantitative accuracy is limited by the partial volume effect (PVE), which stems from the finite spatial resolution of imaging systems. As shown in Fig. 1A, PVE leads to signal spill-over and underestimation of tracer uptake in small or adjacent structures, especially when their size is equal to or smaller than 2–3 times the full-width-at-half-maximum (FWHM) of the imaging system [1, 2, 5–7]. In typical clinical setting, the FWHM values range from 3 to 6 mm for PET scanners and from 7 to 12 mm for SPECT systems, depending on the specific system and reconstruction protocols. Therefore, structures smaller than approximately 6–15 mm in PET or 14–25 mm in SPECT are most susceptible to severe PVEs. Even structures marginally larger than FWHM can suffer partial volume losses due to spill-over effects and motion-related artifacts from cardiac or respiratory activity, underlining the relevance of motion correction algorithms [8–18]. Figure 1B illustrates the impact of voxel size and the resulting tissue-fraction effect at structure boundaries. While both PET and SPECT are affected by PVEs, PET generally offers higher spatial resolution and sensitivity, whereas SPECT is more prone to scatter and collimator-related distortions [19]. As a result, Partial Volume Correction (PVC) strategies optimised for one modality may not directly translate to the other [20]. In SPECT, lower resolution makes PVE more pronounced, particularly in cardiac imaging, dosimetry, and small-lesion quantification [21–23], and advanced techniques such as blood-concentration-based or iterative multi-target correction (MTC) can improve accuracy but remain computationally intensive [24–26].

Addressing PVE improves PET/SPECT accuracy. This can be achieved by enhancing scanner resolution or modelling resolution effects during reconstruction [27–31]. PVC techniques, ranging from region-based (RB) to advanced iterative and reconstruction-integrated methods, provide more elaborate correction [32–45]. Recent studies emphasize the value of post-processing and AI-enhanced reconstruction methods for PVC. Deep learning (DL) techniques show promise in low-dose and high-resolution imaging, and PVC is increasingly recognized as vital in oncology, brain segmentation, radionuclide therapy, and radiation treatment planning, by improving absorbed dose calculation accuracy and lesion delineation [46–51].

Instead of simply updating the comprehensive review published in Physics in Medicine and Biology 13 years ago [34], this review provides a broad and up-to-date survey of PVC in PET and SPECT imaging across neurology, cardiology, and oncology. Our aim is to highlight strengths, limitations, and real-world applicability of PVC methods, with a particular focus on their role in clinical workflows, an aspect not systematically addressed in previous reviews. PVC techniques are categorized into three main groups, reconstruction-based, post-reconstruction-based, and hybrid/AI-based approaches, each with guided or unguided variants. While prior studies have described these methods, significant gaps remain in systematically comparing them across clinical domains and clarifying their deployment in practice. This review addresses these gaps and explores the critical question: Is PVC essential and indispensable for clinical practice, or does its impact vary depending on context, with the potential to be either beneficial or detrimental?

To enable structured comparison, we introduce a ten-category classification framework based on three core dimensions: imaging modality (PET or SPECT), anatomical guidance (guided vs. unguided), and algorithmic approach (conventional vs. AI/hybrid). While a direct combination of these binary dimensions would yield eight categories, the inclusion of hybrid methods, particularly those integrating AI into both reconstruction and post-reconstruction stages, results in ten practically distinct groupings. This framework allows systematic grouping and nuanced comparison of PVC methods across contexts, acknowledging methodological overlaps and highlighting where certain techniques excel. Following the review of existing studies, the discussion section (section V) provides an overall assessment of different PVC techniques, exploring their advantages, limitations, challenges, and opportunities for future application in diverse clinical settings (the reader can immediately skip to that section for a bird’s-eye view of status and prospects of PVC. In particular, we provide Tables in section V summarizing most common PVC algorithms categorized by their benefits, pitfalls, and likelihood of routine clinical deployment). Finally, the conclusion summarizes insights into the overall role of PVC in PET and SPECT imaging, providing a nuanced perspective on its relevance and implications for clinical adoption.

## Overview of partial volume correction strategies

Over the years, PVC methodologies have advanced significantly, leveraging high-resolution anatomical information from CT or MRI to mitigate PVE and improve quantitative PET and SPECT accuracy. These approaches fall into two main categories: post-reconstruction and reconstruction-integrated techniques.

**Fig. 2:**
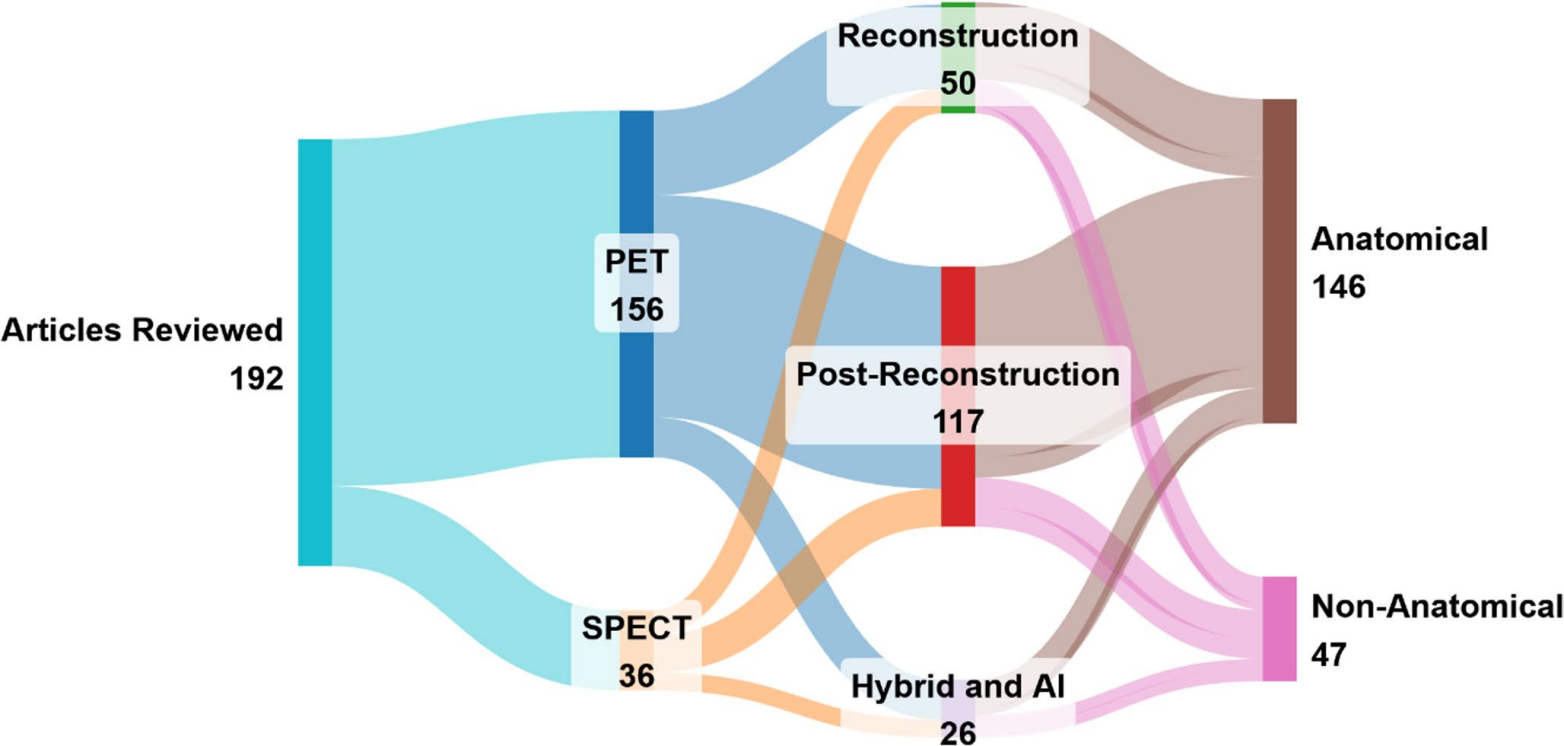
Sankey diagram illustrating the classification of the 192 reviewed articles by imaging modality (PET, SPECT), PVC implementation stage (reconstruction-based, post-reconstruction, or hybrid/AI-based), and anatomical guidance (anatomical vs. non-anatomical). Flow widths are proportional to the number of studies in each category, revealing that most PET studies employed anatomically guided post-reconstruction PVC, while non-anatomical methods remain less represented

Post-reconstruction strategies include regional methods (e.g., Recovery Coefficient (RC) [1], Geometric Transfer Matrix (GTM) [12]) that correct predefined homogeneous regions, and voxel-wise approaches (e.g., Müller-Gärtner (MG) [52], Region-Based Voxel-Wise (RBV) [33], image deconvolution [53]) aimed at restoring localized activity and reducing spill-over. Sinogram-domain methods correct directly on projection data before reconstruction, benefiting from reduced spatial noise correlation and more tractable pipelines [54–57]. Reconstruction-based methods incorporate the system’s point spread function (PSF) into image formation, inherently adjusting for PVE and improving structural fidelity [58]. At the cutting edge, AI-based and simulation-supported strategies are reshaping PVC innovation [59–61]. DL models can adaptively restore resolution from large datasets, while phantom-based simulations provide controlled environments for development and benchmarking [62]. These methodologies are summarized in Fig. 2 summarizes the distribution of the 192 reviewed studies according to imaging modality (PET vs. SPECT), PVC implementation stage (reconstruction, post-reconstruction, or hybrid/AI), and anatomical guidance (anatomical vs. non-anatomical). This visual mapping highlights methodological diversity across the literature, as well as dominant trends in clinical applications and algorithmic strategies.

## PVC approaches in PET imaging

### Reconstruction-based PVC in PET imaging

Reconstruction-based PVC techniques integrate PVE correction directly into the image reconstruction process, thereby improving resolution, quantitative accuracy, and noise characteristics. Depending on the use of anatomical priors (e.g., MRI, CT), they can be categorized into anatomically-guided and non-anatomically-guided approaches. PET-specific applications: In PET, these methods are particularly advantageous in neurology, cardiology, and oncology, where accurate small-structure quantification is critical [63–101]. A comparative overview of PET reconstruction-based PVC strategies is provided in Supplementary Table S1.

#### Anatomically guided reconstruction-based PVC

These approaches embed high-resolution anatomical information, most often from MRI, and occasionally CT, into the PET reconstruction process to reduce PVEs. MRI is frequently preferred for its superior soft tissue contrast, enabling more accurate resolution recovery, lesion detectability, and quantification. Studies have consistently shown that anatomical priors improve PET accuracy: for instance, Vunckx et al. [63] reported that the Bowsher prior enhanced spatial resolution by 15–20% and reduced SUV bias in small cortical regions, while Gutierrez et al. [64] achieved PVE errors below 5% in small brain structures using MRI-guided voxel-based PVC. Other works have integrated advanced priors, such as joint entropy [65, 66] or Markov random fields [67], into reconstruction, balancing noise suppression with detail preservation. MRI-guided methods have proven effective across brain, cardiac, and oncological PET, with applications in tau/amyloid quantification, dopamine transporter imaging, and tumour metabolic activity assessment. Despite these benefits, performance depends heavily on high-quality anatomical data, accurate PET/MRI registration, and reliable segmentation; errors in these steps can induce quantification bias. Computational demands are higher than for non-anatomical methods, but in anatomically rich regions, these techniques remain a gold standard for synergistic PET–MRI reconstructions.

#### Non-anatomically guided reconstruction-based PVC

In the absence of structural imaging, these methods integrate the system’s PSF, statistical priors, or sparsity constraints directly into iterative reconstruction. This enables PVE correction for stand-alone PET systems or cases without MRI/CT. High-resolution PET studies have shown that reconstruction-based PVC methods can improve quantitative accuracy, with within- and post-reconstruction strategies demonstrating benefits in brain FDG data [88]. Further, kernel-based PSF correction approaches have been developed, showing improved PVC performance compared with MR-based methods in brain PET [89]. Quantitative oncology studies reported 10–15% SUV accuracy improvements when applying partial-volume correction during PSF-modelled OSEM reconstruction [92], while generalized PSF modelling further optimized quantitation across a wider range of PET applications [93]. In oncological PET, penalized-likelihood reconstructions enhanced stability of quantitation and improved partial-volume correction [94]. Super-resolution–based methods have also been applied for brain PET, achieving improved correction of partial-volume effects [95]. Finally, dynamic PET studies demonstrated that resolution modelling significantly improved brain kinetic parameter estimation, particularly for FDG modelling [96].

### Post-reconstruction PVC in PET imaging

Post-reconstruction PVC methods apply correction after image reconstruction, using either anatomical priors or intrinsic image properties to mitigate PVEs. This is valuable when advanced reconstruction-based correction is unavailable. PET-specific applications: In PET, these methods have been used extensively across neurological, oncological, and cardiac studies [102–200], as summarized in Supplementary Table S2.

#### Anatomically guided post- reconstruction-based PVC

**Fig. 3:**
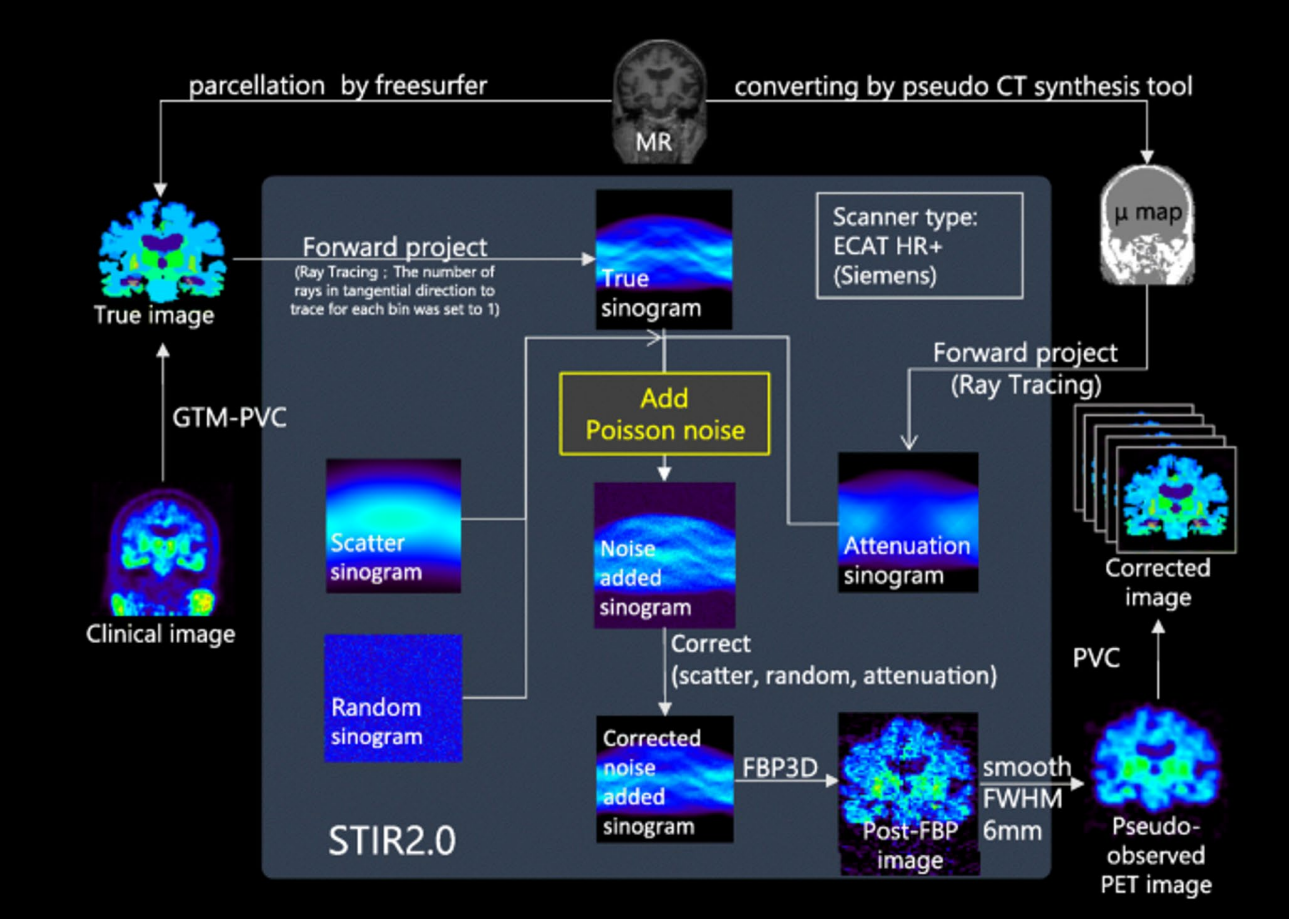
Workflow of the PET PVC process, starting from MR-based segmentation and µ-map generation, followed by forward projection, noise addition, scatter/attenuation correction, and image reconstruction. PVC is applied in the final step to produce corrected PET images ready for analysis. These schematic highlights how anatomical priors and accurate system modelling are integrated to minimize partial volume effects. Reprinted with permission from [163] under a Creative Commons Attribution License (CC BY 4.0 DEED)

**Fig. 4:**
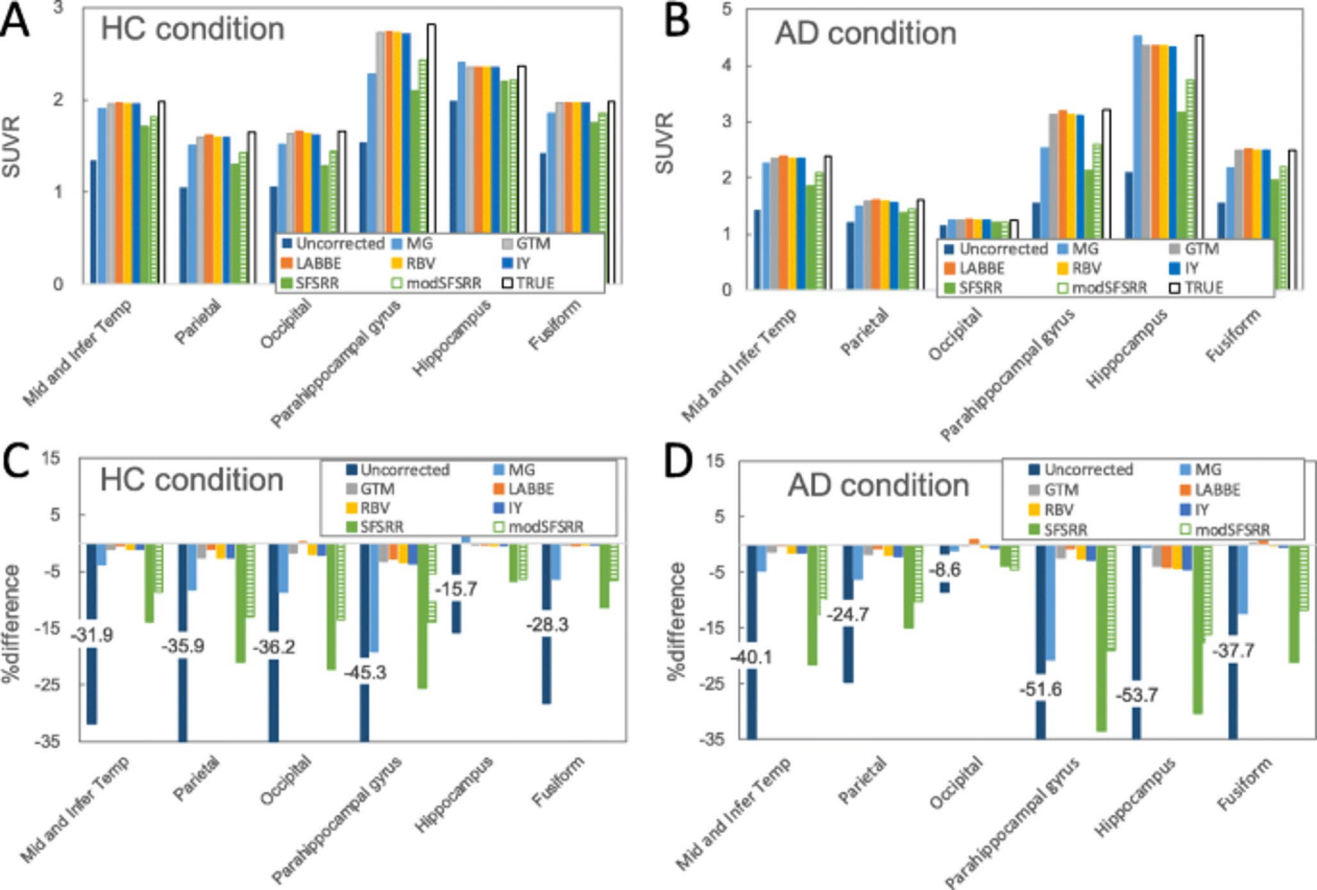
Comparison of SUVR values and percentage differences between pseudo-observed, PVC-corrected, and true SUVRs in HC (**A, C**) and AD (**B, D**) conditions. Methods tested include MG, GTM, RBV, SFSRR, and modSFSRR. Results show that modSFSRR consistently achieves the smallest deviation from true SUVRs, while uncorrected images have the largest bias, particularly in the hippocampus and parahippocampal gyrus for AD patients. Reprinted with permission from [163] under a Creative Commons Attribution License (CC BY 4.0 DEED)

These methods apply correction after PET image reconstruction using anatomical priors, often derived from CT or MRI, to improve the accuracy of quantification. They are widely used in oncology, neurology, and cardiology to enhance lesion detectability and biomarker precision. Applications include tau and amyloid imaging in Alzheimer’s disease, dopaminergic PET in Parkinson’s disease, and brain metabolism mapping. Toolboxes like PETPVC and pipelines such as APPIAN enable standardized implementation, incorporating alignment, segmentation, and quality control. Representative studies highlight their practical impact: Azimi et al. [200] found that radiomic feature reproducibility in brain PET strongly depends on the PVC method, with RVC and RL showing the best stability (>60% features with COV < 25% and ICC ≥ 0.75), while MTC and PLS were least reliable. Gallivanone et al. reported that applying MRI-guided GTM PVC increased tumour SUV_mean_ accuracy by >10% in lung and breast cancer, thereby improving prediction of disease-free survival [110]. Sari et al. [164]. developed a method using MRA and Single-Target Correction (STC) for carotid artery PET-FDG images, restoring at least 92.4% of true signal intensity. Malpas et al. [156] demonstrated that in longitudinal Alzheimer’s PET, PVC revealed uptake increases over 24 months that were not visible in uncorrected scans, underlining the need for both corrected and uncorrected data. However, these gains depend heavily on accurate segmentation and high-quality co-registration, with performance sensitive to MRI resolution and artifacts. Figures 3 and 4 illustrate the complete PVC processing workflow (from MRI-based parcellation to corrected PET output) and compare multiple PVC algorithms (e.g., MG, GTM, RBV, SFSRR, modSFSRR) in Alzheimer’s disease (AD) and healthy control (HC) datasets, showing that advanced methods, like modSFSRR, yield the lowest bias, especially in small structures, such as the hippocampus.

#### Non-anatomically-guided post-reconstruction-based PVC

**Fig. 5:**
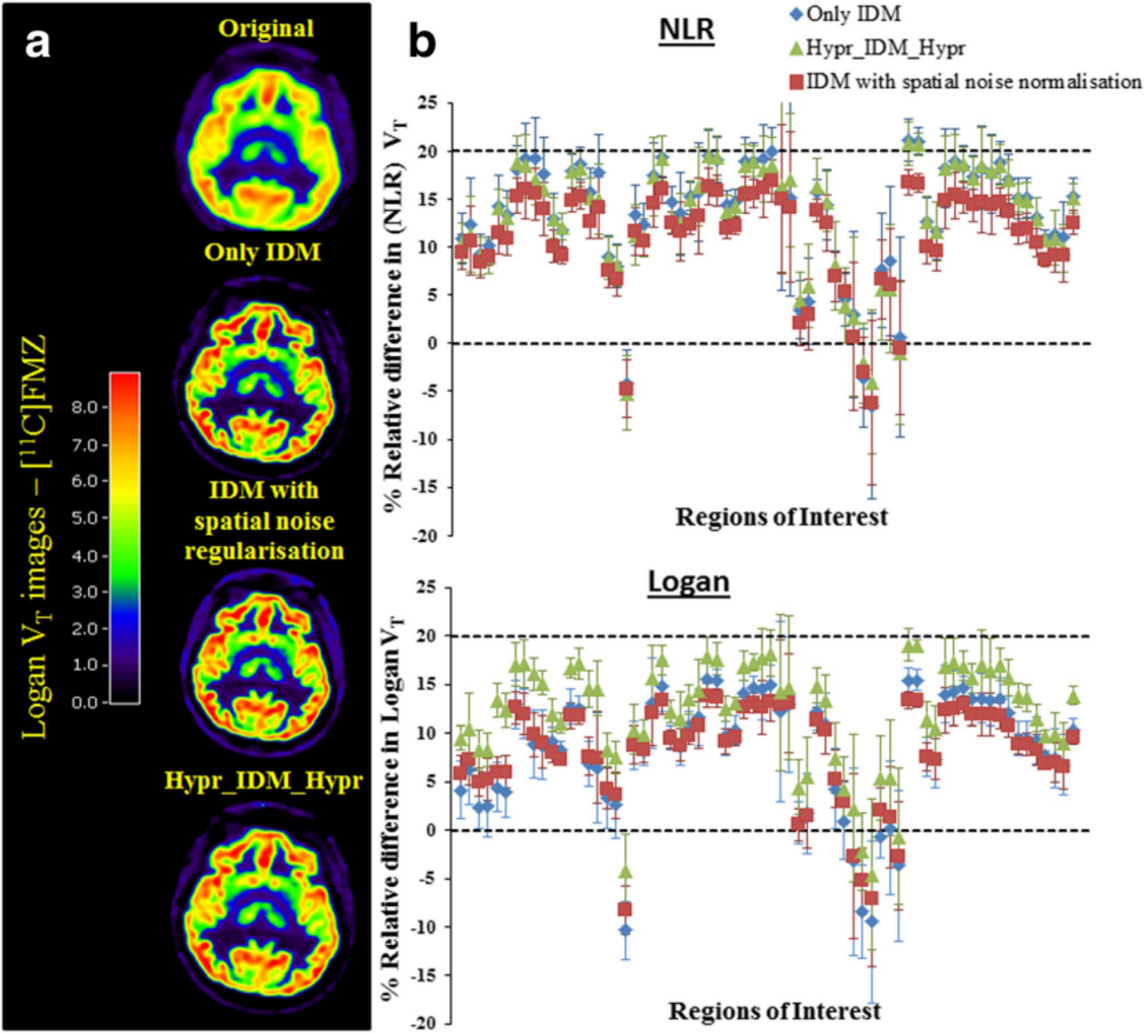
(**a**) Representative [¹¹C]FMZ VT maps from Logan analysis, comparing uncorrected iterative deconvolution (IDM), IDM with spatial noise regularisation, and IDM with HYPR filtering. (**b**) Regional quantitative effects on VT values computed with both NLR and Logan methods, showing reduced bias and variability after noise suppression, particularly in small cortical structures. This highlights the role of advanced denoising in stabilising post-reconstruction PVC results. Reprinted with permission from [195] under a Creative Commons Attribution License (CC BY 4.0 DEED)

These methods enhance PET quantification using statistical models, deconvolution techniques, and machine learning without requiring additional anatomical data, making them valuable when MRI or CT is unavailable. They have been applied in cardiac PET, tumour response assessment, and brain imaging, where they improve SUVs and regional uptake consistency. Hofheinz et al. [188] introduced a model-free GTM variant that improved lesion SUV accuracy by 15–25% without anatomical priors. Golla et al. [195] found that iterative deconvolution with HYPR denoising improved grey matter VT estimation by 12% compared to uncorrected PET. Mikasa et al. [194] showed that dual-time-point standardization reduced inter-scan SUV variability in oncological PET by up to 10%. Hatt et al. observed that applying PVC increased AUC for predicting therapy response in oesophageal cancer from 0.67 to 0.74 [189], with notable improvements in tumour heterogeneity quantification [190, 192]. Despite these benefits, non-anatomical methods can amplify noise and lack structural guidance in heterogeneous or low-contrast regions. Hybrid strategies that combine statistical modelling with anatomical estimation are emerging to mitigate these issues. Figure 5 demonstrates how iterative deconvolution with HYPR denoising improves grey matter quantification in [¹¹C]FMZ PET. The images and plots show that applying spatial noise *regularisation* or HYPR filtering reduces variability and yields VT estimates closer to reference values across multiple brain regions.

## Hybrid and AI-based PVC approaches in PET imaging

Hybrid and AI-based PVC approaches combine classical correction frameworks with machine learning or DL algorithms. These may operate with or without anatomical priors and can augment or replace steps in both reconstruction-based and post-reconstruction-based pipelines. PET-specific applications: PET implementations have shown improvements in small-structure quantification and robustness in multi-tracer studies [201–217]. Detailed examples are summarized in Supplementary Table S3.

### Anatomically-guided hybrid and AI-based PVC

Anatomically-guided hybrid PVC methods enhance PET quantification by combining traditional correction strategies with DL models that exploit structural information from high-resolution modalities, such as MRI. These approaches are especially beneficial in neurological imaging, where precise localization and tissue differentiation are crucial. They have been applied in tracer evaluation, disease phenotyping, and PET/MR synergistic imaging.

**Fig. 6:**
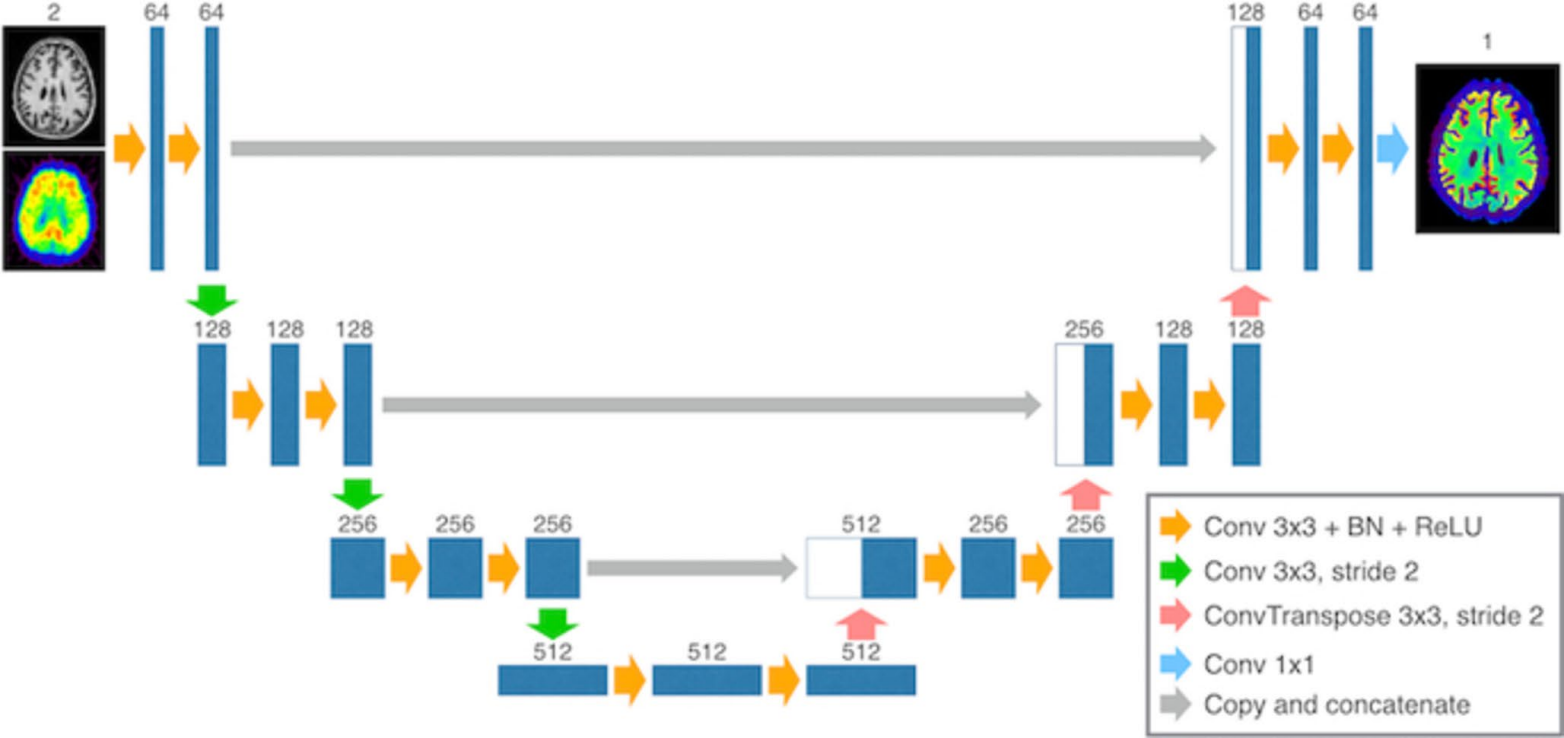
The 2D U-Net model used for predicting PVC maps in brain PET imaging. It features an encoder-decoder architecture with skip connections, convolutional layers, batch normalization, and ReLU activation. Down-sampling and up-sampling are performed with convolutional layers. This architecture enables accurate PVC prediction while preserving fine structural details. The final output is generated through a 1 × 1 convolutional layer. Numbers indicate the number of channels. Reprinted with permission from [205] under a Creative Commons Attribution License (CC BY 4.0 DEED)

**Fig. 7:**
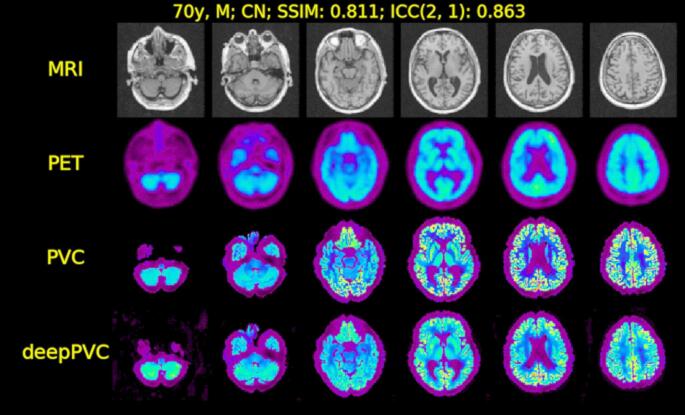
MR, PET, and both real and predicted PVC maps for instances with the highest ICC in the [^18^F]-FDG test data. CN stands for cognitively normal; MR for magnetic resonance; PET for positron emission tomography; and PV for partial volume. The deepPVC predictions show high similarity to reference PVC maps, with improved structural definition and contrast in cortical and subcortical regions. Reprinted with permission from [205] under a Creative Commons Attribution License (CC BY 4.0 DEED)

Xu et al. [201] developed a multi-step framework combining PET segmentation, denoising, and PVC (using Anscombe transformation, iterative volume-consistent PVC, and clustering), improving clarity and quantification accuracy. Song et al. [202] implemented a super-resolution CNN incorporating MRI-derived priors, achieving improved PET image sharpness compared to conventional methods. Zhao et al. [203] showed that PVC improved ^18^F-AV1451 tau deposition measurements in small brain regions, thereby enhancing correlation analyses. Corda-D’Incan et al. [211] proposed Syn-Net for synergistic PET-MR reconstruction, achieving both noise reduction and sharper structural boundaries, while Matsubara et al. [205] introduced deepPVC, predicting PVC maps directly from PET and MRI without requiring segmentation, reducing processing time. Lin et al. [217] introduced MRI-styled PET, a deep-learning fusion framework leveraging T1-weighted MRI to enhance FDG-PET structural details and quantitative accuracy degraded by PVE, achieving higher SSIM and PSNR than conventional anatomy-guided PVC and baseline fusion models. Despite improvements in image sharpness, noise reduction, and SUV recovery, these methods remain sensitive to registration quality, anatomical variability, and diversity of training datasets. Figures 6 and 7 show a representative 2D U-Net-based PVC model for brain PET and its application in [^18^F]-FDG imaging, illustrating architectural components (encoder–decoder, skip connections) and comparative visual results across MR, PET, real PVC, and predicted PVC maps, highlighting consistency in small brain regions and tracer distribution.

### Non-anatomically-guided hybrid and AI-based PVC

Non-anatomically-guided PVC approaches remove the dependency on structural imaging by leveraging DL, statistical modelling, or generative techniques trained directly on PET data. Marsh et al. [209] demonstrated that incorporating PVC into ^124^I-CLR1404 PET/CT improved tumour dosimetry and treatment response assessment in head and neck cancer models.

**Fig. 8:**
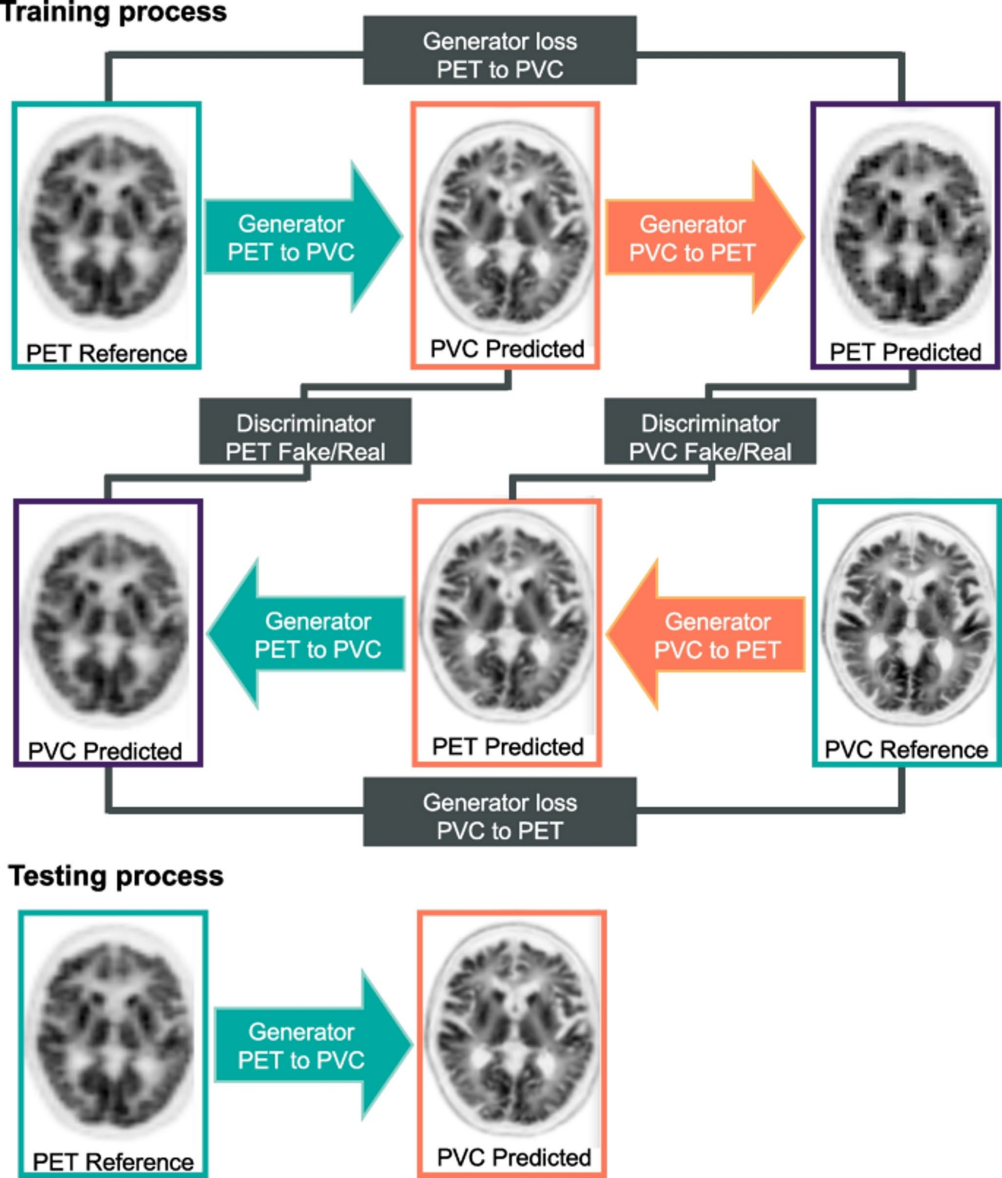
Schematic representation of the Cycle-GAN architecture for PET-PVC synthesis, illustrating the bidirectional transformation between non-PVC and PVC PET images. The upper panel shows the training procedure, while the lower panel details the testing phase. Reprinted with permission from [214] under a Creative Commons Attribution License (CC BY 4.0 DEED)

**Fig. 9:**
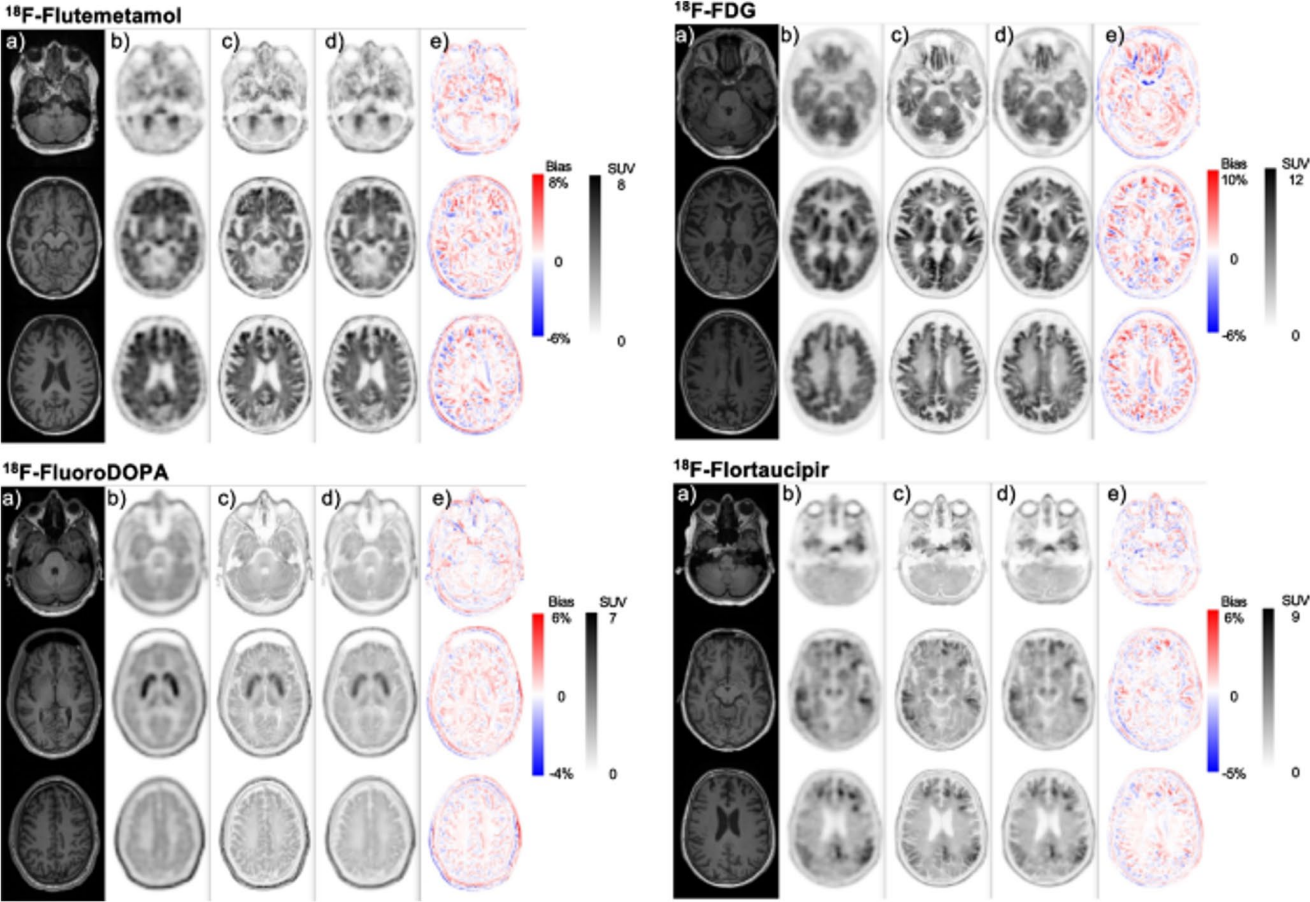
Example slices from multi-tracer brain PET scans of various patients showcasing: (a) co-registered T1-weighted MRI, (b) non-PVC PET images, (c) MRI-guided reference PVC PET images, (d) DL-predicted PVC PET images, and (e) corresponding bias maps. These results highlight the model’s ability to reproduce reference PVC image quality while maintaining tracer-specific uptake patterns. Reprinted with permission from [214] under a Creative Commons Attribution License (CC BY 4.0 DEED)

Jomaa et al. [213] combined iterative deconvolution with shearlet transform, improving SNR and recovery coefficients without anatomical priors. Sanaat et al. [214] introduced a CycleGAN-based PVC framework for multi-tracer datasets, transforming non-PVC PET images into PVC-enhanced images rated visually comparable to original PVC outputs (Figs. 8 and 9). Azimi et al. [215, 216] developed DL architectures capable of generating full-dose PVC PET images and attention-based correction for ^18^F-FDG brain PET, improving image quality without requiring anatomical information.

These models facilitate automated, segmentation-free correction pipelines and show robustness across tracers and acquisition protocols. However, they face challenges in interpretability, generalizability, and standardization of validation pipelines. Performance can vary significantly with scanner characteristics, tracer type, and acquisition conditions, necessitating further multi-centre validation before widespread clinical adoption.

## PVC approaches in SPECT imaging

### Reconstruction-based PVC in SPECT imaging

Reconstruction-based PVC in SPECT follows the same categorization principles described above. SPECT-specific applications: Particularly important in neurological, cardiac, and skeletal imaging, often incorporating CT-based anatomical priors [218–228]. Key methods are summarized in Supplementary Table S4.

#### Anatomically-guided reconstruction-based PVC

These methods leverage structural priors (e.g., CT or MRI) to enhance reconstruction accuracy and quantification. They offer strong performance in contrast recovery and lesion detectability, particularly in brain, cardiac, and skeletal imaging. For example, Erlandsson et al. [218] introduced p-PVC for SPECT, correcting for distance-dependent blurring and integrating filtered back-projection (FBP), which improved quantification with faster processing; a follow-up version using OSEM yielded higher striatal contrast (1.53) than both OSEM with resolution recovery (1.42) and standard OSEM (1.04). Chan et al. [220] applied anatomical priors in cardiac SPECT/CT to reduce noise and improve myocardial uptake quantification, while Liu et al. [221] demonstrated enhanced image quality in low-dose cardiac scans with iterative PVC. Kangasmaa et al. [223] showed that Bayesian CT-guided reconstruction methods (AMAP-S and AMAP-R) provided superior accuracy over OSEM for bone SPECT, with lesion-visible CT scans more accurately depicting lesion shape and size. Hybrid approaches such as SPECTRE [224], which uses diagnostic PET to guide theranostic SPECT reconstruction, have also improved resolution and dosimetry with reduced noise.

Despite these advantages, anatomically-guided methods remain highly dependent on precise segmentation and registration, making them sensitive to misalignment and anatomical variability, and their computational demands can limit scalability in routine clinical practice.

#### Non-anatomically-guided reconstruction-based PVC

These techniques rely on physical modelling (e.g., attenuation, scatter, resolution) without requiring anatomical inputs, and are useful when structural imaging is unavailable or unreliable. Morphis et al. [226] used Monte Carlo–based correction for attenuation, scatter, and collimator response in ^99m^Tc and ^198^Au SPECT/CT, achieving reliable quantification of small sources. Similarly, Leube et al. [227] reported that resolution modelling in ^177^Lu-SPECT/CT substantially improved recovery coefficients, especially for small spheres (up to 5.8), though positional variability increased, underlining the need for harmonized acquisition protocols. While such methods are modality-independent and avoid dependency on CT/MRI, they are more susceptible to noise and less effective in recovering small or ambiguous structures. Moreover, despite the accuracy of Monte Carlo simulations, their high computational demands and reduced robustness in complex anatomy still restrict widespread clinical adoption.

### Post-reconstruction PVC in SPECT imaging

Post-reconstruction PVC in SPECT is applied in settings where raw projection data are unavailable or reconstruction methods are limited. SPECT-specific applications: Frequently used for brain and myocardial perfusion imaging [21, 22, 24–26, 229–241], with examples listed in Supplementary Table S5. Among post-reconstruction PVC techniques, perturbation-based GTM (pGTM) has shown promise in SPECT for reducing Gibbs artifacts and improving myocardial perfusion imaging. However, challenges such as CT–SPECT registration errors, resolution mismatches, and difficulty in separating small anatomical structures can still limit accuracy.

#### Anatomically-guided post-reconstruction-based PVC

These methods apply anatomical priors (typically from CT or MRI) after image reconstruction to correct for PVEs and have shown strong results in cardiac and neurological SPECT. For instance, Liu et al. [229] used an automatic multi-atlas segmentation approach in cardiac SPECT/CT, improving image quality and quantification; Ren et al. [232] optimized PVC factors in ^99m^Tc-PYP SPECT, enabling reproducible differentiation of ATTR cardiomyopathy; Furuta et al. [24] demonstrated improved SBR and SUR accuracy in ^123^I-FP-CIT SPECT, reducing SUR error to 6.2%. In radiopharmaceutical therapy, Liu et al. [21] evaluated three PVC methods (RC-PVC, RVC, IY) in ^177^Lu-PSMA-617 SPECT/CT, with IY and RC-PVC markedly reducing tumor MAE and improving kidney quantification.

**Fig. 10:**
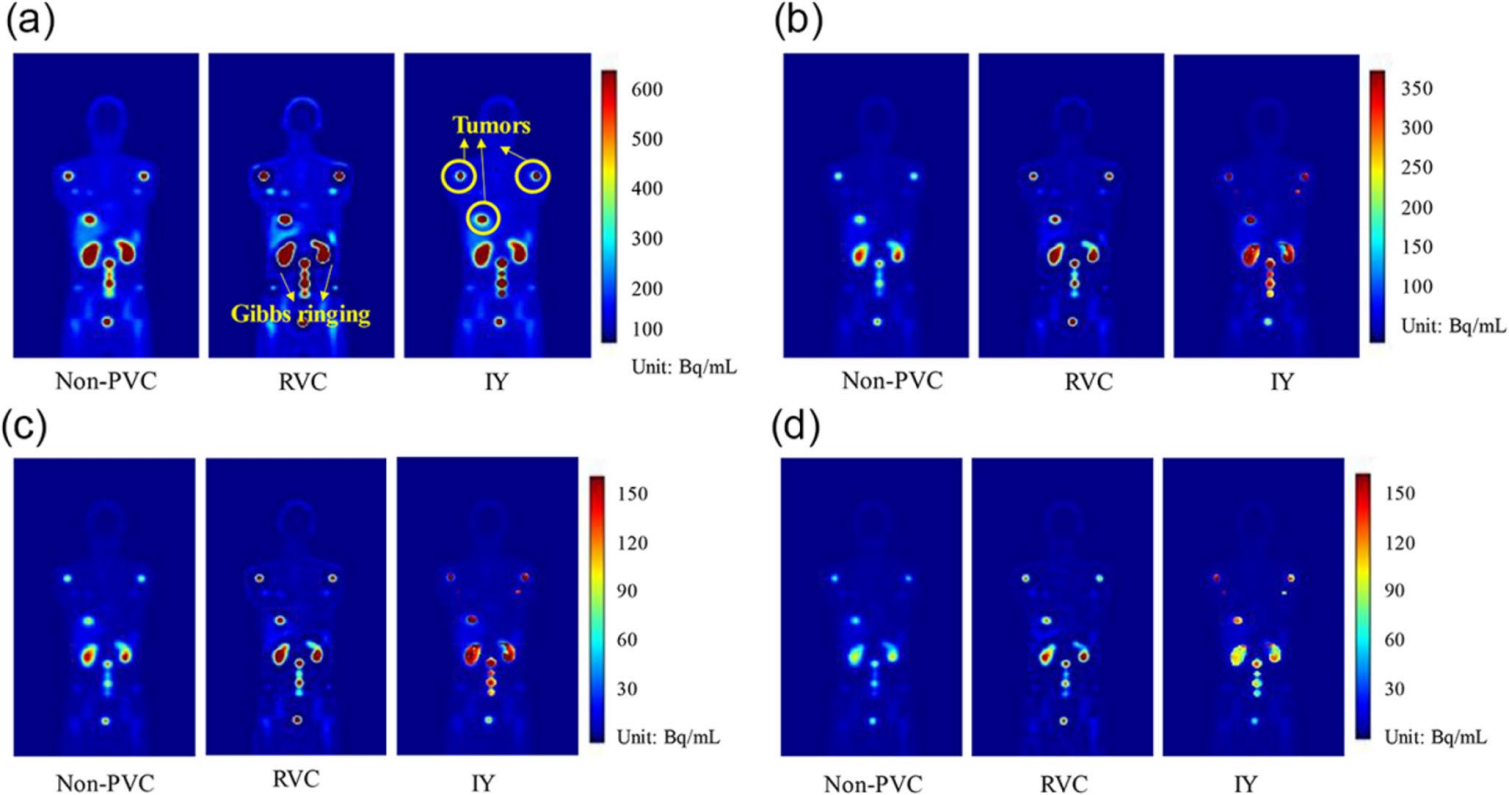
Representative non-PVC reconstructed images and PVC results (RVC and IY) for an XCAT phantom at four imaging time points: (**a**) 2 h, (**b**) 20 h, (**c**) 40 h, and (**d**) 200 h. The PVC-corrected images show improved activity recovery and clearer delineation of structures compared to non-PVC images, particularly at earlier time points when partial volume effects are more pronounced. Reprinted with permission from [21] under a Creative Commons Attribution License (CC BY 4.0 DEED)

**Fig. 11:**
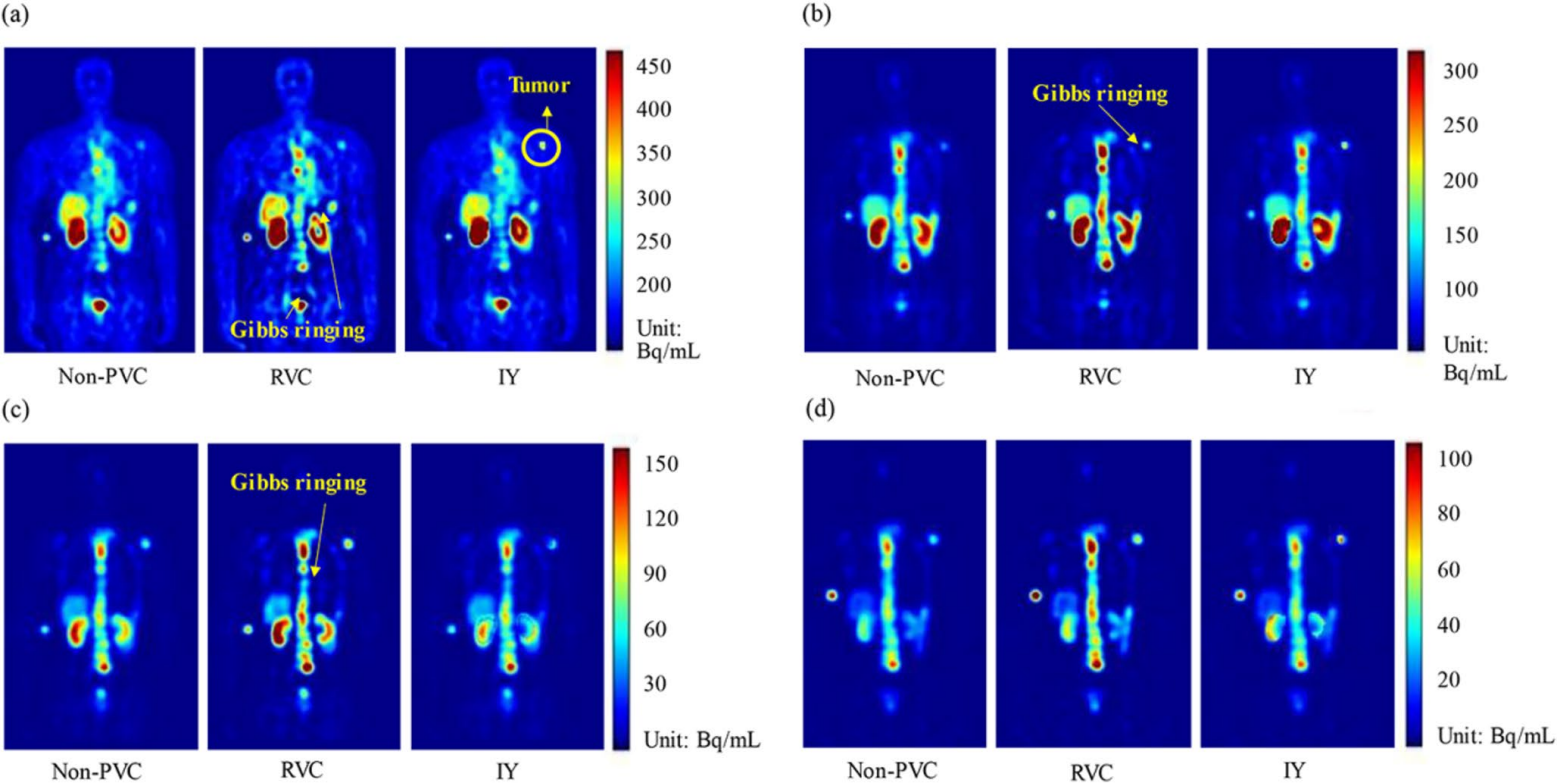
Representative clinical SPECT images for a patient, with corresponding PVC results (RVC and IY) at (**a**) 2 h, (**b**) 20 h, (**c**) 40 h, and (**d**) 200 h imaging time points. The PVC-corrected images demonstrate enhanced lesion contrast and quantitative accuracy, reducing underestimation of activity evident in non-PVC reconstructions. Reprinted with permission from [21] under a Creative Commons Attribution License (CC BY 4.0 DEED)

Strengths of anatomically-guided post-reconstruction PVC include improved accuracy in standardized uptake metrics (SBR, SUR, RC), reduced quantification error in both phantom and patient data, and enhanced reproducibility in dose mapping. However, performance depends heavily on accurate segmentation and inter-modality registration, and errors in alignment or subject-specific anatomy can degrade correction accuracy. The computational complexity and requirement for high-quality anatomical imaging may limit adoption in resource-constrained settings. Figures 10 and 11 illustrate representative examples from phantom and clinical SPECT datasets, showing how RVC and IY PVC methods improve activity recovery over time across different imaging time points (2–200 h).

#### Non-anatomically-guided post-reconstruction-based PVC

These techniques correct PVEs using statistical models, blind deconvolution, or RC scaling without relying on structural imaging. Yin and Chiu [26] demonstrated that segmentation-free blind deconvolution with anatomical filtering improved quantification in ^123^I-mIBG cardiac SPECT/CT without pre-measured PSF. Finocchiaro et al. [22] used phantom-derived RCs to enhance activity estimation in PRRT with ^177^Lu-labeled analogues, whereas Ramonaheng et al. [237] showed via Monte Carlo simulations that optimized RCs and calibration factors significantly improved ^177^Lu SPECT accuracy. Azimi et al. [240] applied PVC in ^177^Lu-PSMA therapy planning, reducing RMSE by 2.83% and improving dose homogeneity.

**Fig. 12:**
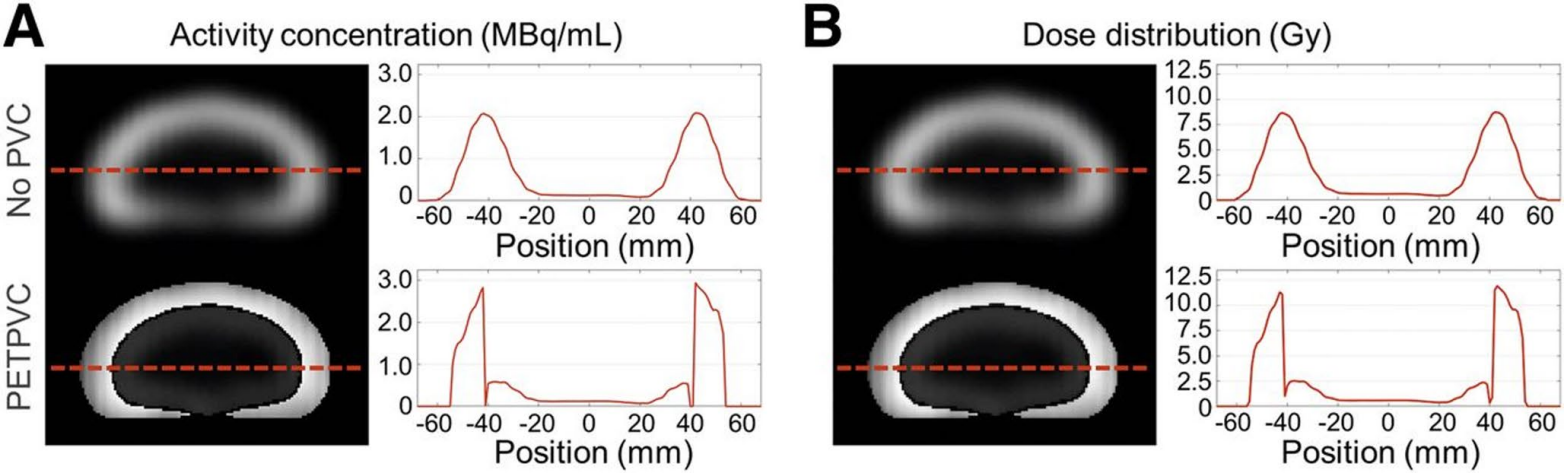
Effects of partial volume correction (PVC) on quantitative accuracy in molecular radiotherapy. (**A**) Activity concentration maps (MBq/mL) and corresponding line profiles across the kidney phantom before (No PVC) and after PVC. (**B**) Dose distribution maps (Gy) and line profiles for the same cases. Data were reconstructed using Flash3D (48 iterations, 1 subset, 8-mm Gaussian filter). PVC markedly improves boundary definition between compartments, recovers true activity levels, and yields dose distributions that align more closely with nominal reference values, highlighting its clinical relevance for accurate dosimetry. Reprinted with permission from [236] under a Creative Commons Attribution License (CC BY 4.0 DEED)

The appeal of non-anatomical methods lies in simplicity, segmentation independence, and adaptability across tracers and protocols. However, their performance can vary with tracer type, lesion size, noise level, and camera resolution. Without anatomical constraints, they may lack spatial specificity and struggle in heterogeneous or low-contrast regions. Nevertheless, voxel-based and RC-based corrections, particularly in radiopharmaceutical therapy, have shown clear clinical value. As illustrated in Fig. 12, applying PVC to a 2-compartment kidney phantom markedly improves activity concentration and dose distribution profiles, especially at organ boundaries, yielding results that better match nominal reference values.

### Hybrid and AI-based PVC in SPECT imaging

The same general concept of hybrid and AI-enhanced PVC applies to SPECT. SPECT-specific applications: Early work has shown promise in combining anatomical priors with AI to improve quantification [23, 242–248]. Representative studies are summarized in Supplementary Table S6. In AI-based PVC, DL approaches have demonstrated encouraging results in SPECT, such as artifact reduction and improved activity recovery in ^177^Lu SPECT/CT, but these techniques remain experimental and require further validation before routine adoption.

#### Anatomically-guided hybrid and AI-based PVC

**Fig. 13:**
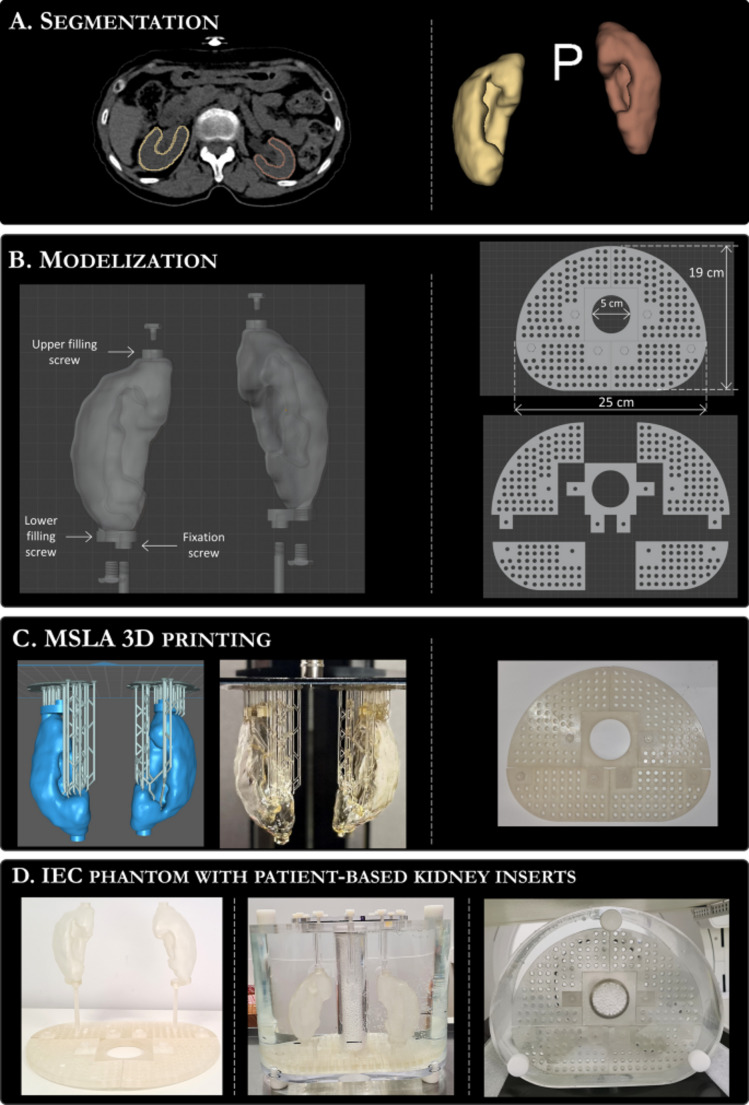
Workflow for designing and fabricating patient-specific kidney inserts for PVC validation in ^177^Lu SPECT imaging. Steps start with (**A**) manual segmentation of kidneys from 68Ga-DOTATOC PET/CT scans using 3D-Slicer, followed by (**B**) creation of a modular support system in Blender tailored for IEC phantom integration. (**C**) The kidney inserts and supports are then produced using MSLA 3D printing technology, ensuring anatomical fidelity. (**D**) The final assembly integrates the patient-based inserts into the IEC phantom, enabling realistic evaluation of anatomy-based PVC performance in quantitative imaging studies. Only one representative pair of inserts is shown. Reprinted with permission from [23] under a Creative Commons Attribution License (CC BY 4.0 DEED)

Anatomically-guided hybrid and AI-based PVC methods in SPECT imaging combine the structural specificity of CT/MRI priors with the automation and adaptability of AI algorithms or physics-based modelling. Adam et al. [242] used a 3D-printed phantom to validate a Monte Carlo SPECT-based dosimetry workflow for ^131^I therapy, refining activity predictions. Yousefi et al. [243] quantified intramyocardial blood volume using ^99^mTc-RBC SPECT/CT, demonstrating the clinical feasibility of integrating anatomical priors. Salvadori et al. [23] improved kidney activity quantification in ^177^Lu SPECT through anatomy-based PVC combined with patient-specific segmentation and 3D-printed phantom inserts. As shown in Fig. 13, the process includes segmentation of kidney structures from PET/CT data, CAD-based model design, MSLA 3D printing, and integration into an IEC phantom for validation studies.

These techniques have shown promise in improving spill-out correction in organ-focused applications like kidney and myocardial imaging, where accurate uptake estimation is critical for therapy planning. By reducing reliance on manual processing and enhancing reproducibility, they help standardize quantitative outputs. However, their clinical scalability remains limited by the need for consistently high-quality anatomical inputs, challenges in multimodal registration, and restricted generalizability from narrow training datasets. Without robust multi-centre validation, their wider applicability is still uncertain, though targeted dosimetry use cases appear increasingly well-supported.

#### Non-anatomically-guided hybrid and AI-based PVC

**Fig. 14:**
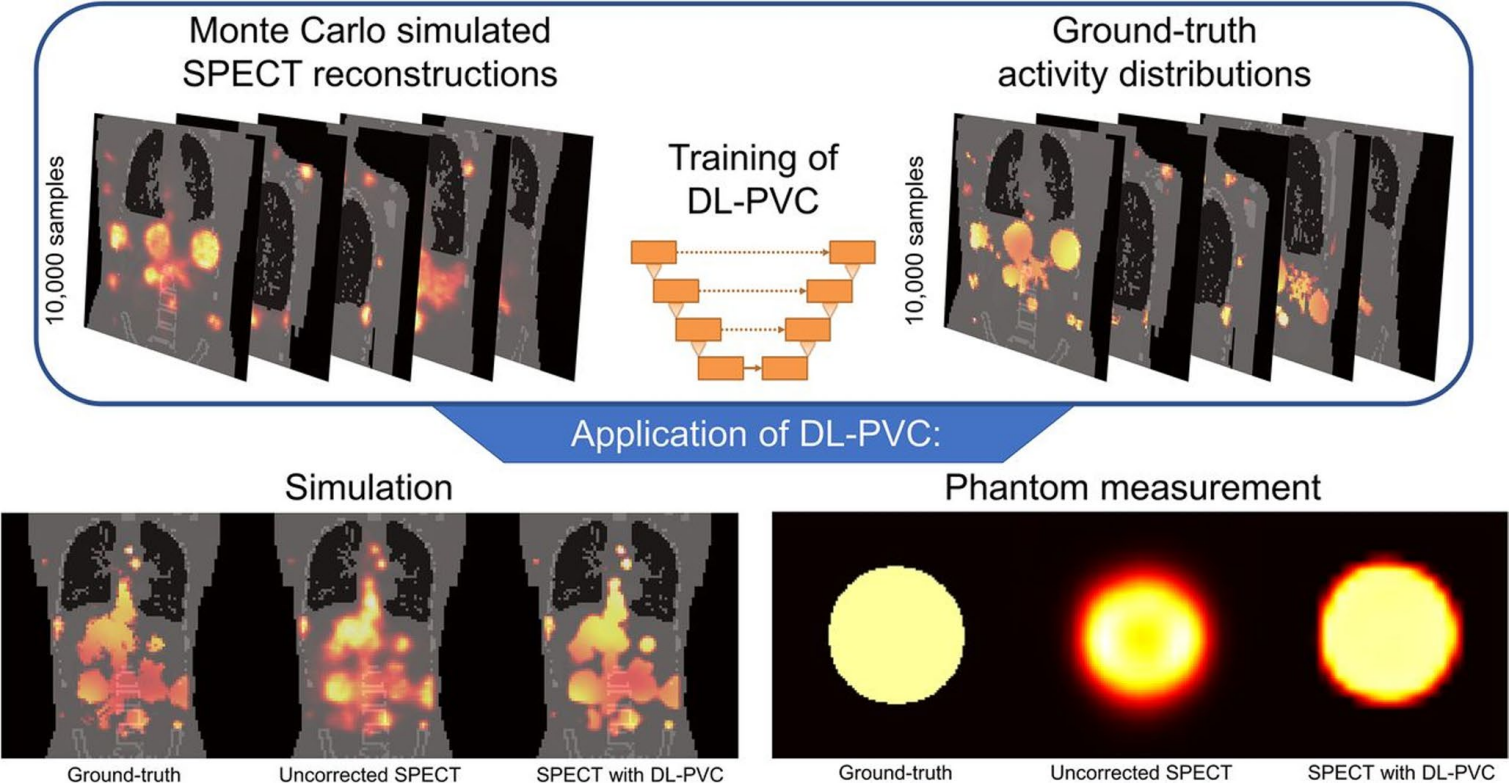
Workflow and results of the deep learning–based PVC (DL-PVC) method for ^177^Lu SPECT/CT imaging. The upper panel shows Monte Carlo–simulated SPECT reconstructions and corresponding ground-truth activity distributions used to train the DL-PVC model with 10,000 samples. The lower panel demonstrates the application of the trained model, comparing ground-truth data, uncorrected SPECT, and DL-PVC–corrected images in both simulation and physical phantom measurements. DL-PVC substantially improves activity recovery and spatial accuracy, especially in small or high-contrast structures. Reprinted with permission from [246] under a Creative Commons Attribution License (CC BY 4.0 DEED)

In contrast, non-anatomically-guided hybrid and AI-based PVC approaches remove the requirement for structural priors by relying on DL models or perturbation-based corrections. Gillen et al. [244] demonstrated improved SPECT quantification using case-specific PSF-based PVC, highlighting the potential of perturbation-guided strategies. Xie et al. [245] proposed a DL-based PVC for cardiac SPECT that required no anatomical guidance, achieving results comparable to anatomically-guided methods and simplifying clinical deployment. Leube et al. [246] developed and validated a deep learning–based PVC method for ^177^Lu SPECT/CT, trained on Monte Carlo–simulated datasets, which significantly improved activity recovery and spatial accuracy in both simulated and physical phantom experiments (Fig. 14). Wang et al. [247] found that an AttGAN model outperformed U-Net, VC, and non-PVC approaches in reducing noise and improving ^99^mTc-TRODAT-1 quantification for Parkinson’s detection, with clinical results aligning with simulations.

These approaches offer segmentation independence, real-time applicability, and compatibility with diverse tracers. However, clinical translation is challenged by the “black-box” nature of DL, limited interpretability, and a lack of standardized validation pipelines. Performance can vary based on training data diversity and scanner-specific factors. While these models are increasingly central to automated PVC pipelines, broad adoption will require transparent validation, robust generalization, and regulatory readiness.

## Discussion, Challenges, limitations and outlook

PVC is widely used in quantitative PET and SPECT imaging to mitigate PVEs, providing not only enhanced tumour detection but also more accurate quantification, including improved SUV/SUVR recovery, reduced small-lesion bias, and more reliable dosimetry, though its effectiveness still varies depending on lesion size, cancer type, and imaging modality [20, 113, 139, 249]. While PVC improves sensitivity, particularly in small lesions, it often reduces specificity and introduces inconsistencies in diagnostic accuracy [164, 168, 184, 185]. In oncology, studies have shown mixed results: PVC enhances tumour detection in head and neck cancers but has limited value in oesophageal cancer [173, 174, 178]. Differences in PVC methodologies, lesion delineation, and biological diversity contribute to these variations [162, 163, 176, 177, 183].

Most oncological PVC applications use simple RC-based methods, which assume spherical tumours with uniform activity distribution. However, these methods are highly sensitive to tumour size estimation errors, leading to biases, either over- or under-estimation, in SUV/SUVR and in absolute quantification [227, 236, 244]. Studies on relapsed/refractory non-Hodgkin’s lymphoma show that while PVC improves metabolic activity assessment, it cannot fully correct PVE in small tumours due to spatial resolution limitations [19, 235]. Additionally, iterative deconvolution methods (e.g., Lucy-Richardson) introduce noise and artifacts, reducing clinical feasibility [179, 181, 183]. For longitudinal imaging, errors in PSF modelling, PET-MRI registration, and segmentation propagate through PVC algorithms, degrading accuracy [155, 224, 230, 232]. Despite its promise, PVC struggles with standardization, with current methodologies failing to reach a gold standard [234, 237, 242].

Across domains, the value of PVC is task-driven rather than method-specific [177, 181, 188]. In neurology, PVC improves small-structure SUVR/VT and group-level effect sizes when registration and segmentation are robust, especially in regions prone to spill-over (e.g., hippocampus, cortical ribbon) [165, 167–169, 176, 178, 179, 183]. In oncology, benefits concentrate on sub-centimetre lesions and quantitative endpoints (SUV/SUVR, TLG, dosimetry), whereas routine diagnostic gains remain inconsistent across tumour types; size-stratified analyses and protocol harmonisation are essential [181, 182, 184, 185, 188–190, 193]. In cardiology, PVC can refine perfusion and volumetric indices provided PET/SPECT–CT alignment and motion control are reliable [220–222, 226, 230]. These distinctions underscore aligning PVC choice, QC steps, and decision thresholds with the intended clinical endpoint. While both PET and SPECT suffer from PVEs, the nature and severity of PVEs differ between the modalities. PET generally offers higher spatial resolution and improved sensitivity, while SPECT is more prone to scatter and collimator-related distortions, with PVE remains a major source of error in Lu-177 SPECT/CT, particularly for structures smaller than approximately three times the system’s FWHM. Consequently, PVC strategies optimized for PET may not directly translate to SPECT and vice versa. The implications for resolution modelling, noise propagation, and algorithmic robustness must be considered modality-specific. In SPECT imaging, PVE is more pronounced due to lower spatial resolution, making PVC potentially more impactful for cardiac imaging, dosimetry, and small lesion quantification [21–23]. Advanced PVC techniques, such as blood-concentration-based and iterative MTC, improve quantification accuracy but remain computationally intensive [24–26]. Some studies show that GTM and RBV increase group separation but also introduce noise and repeatability issues [209, 243, 246]. DL approaches like DeepPVC have improved processing speed, but their accuracy in small brain regions remains inferior to conventional methods [205, 214–216]. Additionally, in ^99m^Tc-tetrofosmin SPECT, PVC reduced activity bias in perfusion defects, but CT-SPECT misalignment remained a limitation [220–222, 243, 244].

Current evidence does not justify universal, across-the-board use of PVC in routine PET and SPECT imaging. Instead, we advocate a standardised, task-aligned workflow: (i) harmonised acquisition/reconstruction protocols and explicit PSF modelling across centres and vendors (as promoted by EARL accreditation), (ii) validation using shared digital/physical phantoms and recovery-coefficient–based, size-stratified analyses, (iii) consensus guidelines for segmentation and registration per organ/lesion, with integrated QC steps, and (iv) publicly available, annotated datasets and phantom studies for benchmarking and reproducibility [24, 31, 58, 144, 165, 179, 222, 238]. Corrected and uncorrected readouts should be reported side by side with endpoint-specific thresholds, with PVC generally recommended when the target structure is smaller than 2–3 times the system’s FWHM (i.e., ≤ 10–15 mm in PET and ≤ 15–25 mm in SPECT), noting that full recovery is rarely achieved for sub-millilitre volumes, underscoring persistent limitations of PVC for very small structures, and therefore, lesions ≤ 10 mm should be excluded from quantitative analyses unless validated PVC is available [199, 249]. For broad clinical adoption, implementations must be simpler, robust, and fast, preferably reconstruction-integrated or well-validated PET-only pipelines with explicit QC for registration and motion, so that bias–variance trade-offs are controlled and results remain reproducible [250–252]. These steps align with ongoing quantitative imaging harmonisation initiatives from SNMMI/EANM procedure standards and theranostic dosimetry guidance endorsing harmonised PVC, including the EANM Research Ltd. (EARL) accreditation program, which sets performance standards and harmonised PET/CT reconstruction protocols to ensure cross-centre comparability and reproducibility [253–256].

While Marquis et al. [51] introduced anatomically-informed RC models that significantly improved mean quantification accuracy in PET/SPECT, their evaluation framework did not assess precision or robustness under varying conditions. Reliability and generalizability of such PVC methods remain to be thoroughly investigated, and performances of such correction strategies, and associated software tools, may be task-specific and require validation for different clinical scenarios.

The use of PVC in SPECT imaging is somewhat distinct, with Perturbation-based GTM (pGTM) as an approach of good potential for PVC [42]. This method is particularly effective in addressing Gibbs artifacts in low-resolution systems like SPECT with parallel-hole collimators. In cardiac SPECT, PVC has proven valuable for improving myocardial perfusion imaging, enabling clearer visualization of ischemic regions and scars. However, significant challenges persist, including resolution mismatches and registration errors between CT and SPECT, leading to inaccuracies in parameters like intramyocardial blood volume (IMBV). Moreover, signal spillover in small or boundary regions often remains uncorrected, and in some cases, differential methods result in negative voxel values. Anatomical structures like myocardial walls or ventricular blood in non-contrast CT scans may be poorly distinguished, impacting PVC accuracy. While anatomy-based methods have shown promise in voxel-based dosimetry, precise image reconstruction and correction of PVE remain critical for improved estimates. Emerging techniques, such as DL-based PVC, have demonstrated encouraging results in applications like ^177^Lu SPECT/CT, offering artifact correction and improved activity recovery [246]. However, these approaches are still experimental, requiring further development for routine clinical use. The future of PVC in SPECT imaging lies in developing larger disease-specific datasets, standard evaluation metrics, and seamless integration of advanced correction tools into clinical workflows. The structure of Table 1 reflects the categorization of PVC studies based on imaging modality (PET, SPECT, or both) and clinical focus. While several pitfalls and challenges, such as segmentation inaccuracies, noise amplification, or dependency on high-resolution anatomical imaging, are inherently relevant to both PET and SPECT, they are allocated here according to the primary modality focus in the reviewed studies. This facilitates a comparative view of method-specific advantages, technical barriers, and translational prospects.

Post-reconstruction PVC can improve recovery but often amplifies mid-frequency noise (e.g., RL ringing) and remains vulnerable to PET–anatomy misregistration and segmentation variability. GTM/RBV may elevate noise and struggle with complex signals and small regions, while MG/SGTM depend heavily on high-quality MRI and accurate tissue labelling. Parallel level set (PLS) approaches likewise introduce risks of over-smoothing or instability in heterogeneous regions. More practical implementations therefore combine deconvolution with advanced denoising, integrate respiratory/cardiac or data-driven motion correction, and apply automated checks to mitigate registration or segmentation errors. Minimising manual VOI delineation remains critical to reduce operator dependence and variability. Taken together, these safeguards are essential if post-reconstruction PVC methods are to become reliable and reproducible enough for routine clinical adoption [107, 144, 179, 200].

**Table 1:** Summary of the benefits, pitfalls, challenges, limitations, and future outlook of studies focusing on PVC in PET and SPECT imaging systems. Rows are grouped by imaging modality and clinical domain (e.g., oncology, neurology, therapy planning)

Modality	Benefits	Pitfalls	Challenges	Outlook
PET	Enhances sensitivity for smaller lesions and improves treatment response prediction in cancers such as head and neck.	Reduced specificity and inconsistent improvement in diagnostic accuracy unless SUV/SUVR thresholds are appropriately adjusted.	Lack of standardized PVC protocols and inconsistencies in lesion delineation.	Development of DL-based PVC (e.g., DeepPVC) holds promise but requires further optimization for small regions.
	Improves accuracy of kinetic parameters like VT and BPND, aligning them with SUV and TBR.	Post-reconstruction PVC methods introduce artifacts such as Gibbs ringing and edge overshoot, and noise amplification limits the clinical applicability of iterative deconvolution methods (e.g., Lucy–Richardson).	Misregistration between PET and MRI reduces alignment accuracy for GTM and MG-based PVC methods.	AI advancements, including FastPET and diffusion models, show potential to overcome PVC challenges.
	Enhances quantification in amyloid and tau studies with reduced signal spill-in/spill-out using RBV techniques.	Limited impact on lesions ≤ 10 mm due to segmentation inaccuracies and noise amplification, with PVC benefits diminishing in longitudinal studies due to added noise and potential data distortion.	High dependency on accurate PSF modelling and segmentation precision.	Emerging hardware (e.g., LAFOV scanners) could address PVE challenges in multi-organ studies.
	Shows utility in early disease detection (e.g., Huntington’s disease gene carriers) by capturing metabolic changes.	High dependency on high-resolution anatomical information (e.g., MRI or CT) and segmentation accuracy, with no clear gold standard for method comparison; MRI-based PVC used as a reference remains imperfect.	Limited generalizability of DeepPVC due to reliance on tracer-specific features.	Research should prioritize harmonizing PET-MRI data integration and optimizing DL-PVC for multi-task applications.
SPECT	Improves quantitative accuracy in myocardial perfusion imaging and dosimetry, especially for small objects.	Post-reconstruction PVC methods amplify noise and struggle with spillover correction in small or boundary regions, particularly in low-resolution systems.	Alignment issues between CT and SPECT reduce accuracy in hybrid imaging.	Deep learning (e.g., DL-PVC) and Monte Carlo simulations hold potential for improving PVC in SPECT.
	Reduces errors in myocardial blood volume (IMBV) estimates and enhances dose differentiation in kidney imaging.	Overcorrection by PVC may lead to overestimated specific binding ratios (SBR) in some cases, and current methods often fail to fully correct PVE in non-uniform tissue distributions or small anatomical structures.	Voxel-level dosimetry for anisotropic SPECT resolution requires optimization and adaptation; full recovery is rarely achieved for sub-mL volumes, underscoring limitations in very small structures.	Automating segmentation processes and adapting PVC methods for fast-paced clinical workflows are key.
	Advanced PVC methods like RGTM improve accuracy in challenging conditions (e.g., small nucleus accumbens regions).	PSF modelling often assumes spatial invariance, overlooking motion-related or system-induced variations, and noise/artifacts (e.g., Gibbs) remain significant obstacles in fast clinical workflows.	Computationally intensive segmentation and motion artifacts hinder clinical adoption.	Standardizing calibration and recovery factors across centers can enhance reproducibility and utility.
	Enhances spatial resolution and signal differentiation in SPECT imaging (e.g., with methods like SPECTRE).	Dependence on high-resolution CT for anatomical guidance limits routine clinical use; absence of commercial PVC software and computational intensity hinder broader adoption.	Errors in segmentation and resolution mismatches between CT and SPECT reduce quantification accuracy.	Validation of advanced methods like DL-PVC and multi-resolution modelling can pave the way for clinical adoption.
PET/SPECT	Enhances dosimetry accuracy in radionuclide therapies such as Lu-177 theranostics, improving personalized treatment planning and therapeutic monitoring by reducing PVE-related errors, which are particularly pronounced for structures < 3×FWHM	Lack of standard evaluation metrics and high dependency on high-resolution anatomical imaging (e.g., MRI or CT), particularly in radiotherapy planning (RTP), limit consistent application; such imaging may not always be available, reducing reproducibility and clinical reliability.	The need for standardized and reproducible PVC methods remains a key challenge, as no clear gold standard exists for method comparison, and most current evidence is still phantom-based with limited patient validation.	Deep learning models like DeepPVC, FastPET, and diffusion models show promise but still require optimization and validation across different tracers. Personalized dosimetry in theranostics (e.g., Lu-177, Tb-161) is an emerging application.
PET/SPECT	Enhances delineation of PTV and BTV by reducing PVE, leading to better dose distribution and improved tumor targeting	Increased computational complexity and dependency on segmentation accuracy may introduce errors in volume definition, while heterogeneous tracer uptake (e.g., due to perfusion changes) can reduce the accuracy of anatomy-based methods.	Standardization of PVC algorithms for RTP remains a challenge, as different correction methods may yield variable outcomes; generalizability across scanners, tracers, and protocols remains limited.	Integration of AI-driven PVC methods with adaptive radiotherapy planning could optimize treatment personalization and dose accuracy.

From a clinical perspective, only a limited subset of PVC methods, most notably PSF-integrated reconstruction techniques, are currently adopted in routine clinical workflows, as they are embedded in commercial PET and SPECT software. Other methods, including anatomically guided approaches (e.g., PETPVC) and AI-driven or voxel-wise techniques, are still predominantly used in research settings due to limited validation and lack of regulatory approval. These distinctions are reflected in Table 2 and illustrated in Figure S1, which provide an overview of the clinical readiness and translational maturity of various PVC methods.

While advancements in DL offer promise for PVC without anatomical data, these methods are still in early stages and require extensive validation. Looking ahead, hardware advancements, especially the emergence of long axial field-of-view (LAFOV) PET/CT scanners, such as the uEXPLORER, PennPET Explorer, and Biograph Vision Quadra, have shown strong potential in addressing PVE challenges [257]. These systems provide dramatically higher sensitivity, improved lesion detectability, and the capability for comprehensive (e.g., whole-body) imaging protocols without bed translation. For example, the uEXPLORER offers up to 15–68× higher sensitivity compared to conventional PET, while both PennPET Explorer and Biograph Vision Quadra demonstrated improved signal-to-noise ratios and spatial resolution, enabling lower-dose or faster scans [258]. Studies by Mannheim et al. [259] have shown significant variations in PVE across the FOV, driven by object size, signal-to-background ratio, and isotope type, with contrast recovery coefficient (CRC) differences reaching up to 50%. These findings underscore the importance of accounting for FOV-dependent PVE variability in clinical interpretations, particularly in multi-organ or dynamic studies. Furthermore, the increasing use of novel radiopharmaceuticals, such as ¹⁶¹Tb, ¹⁷⁷Lu, and ²²⁵Ac theranostic agents used for radiopharmaceutical therapies, will likely require adapted PVC methods that account for their unique spatiotemporal biodistributions. Future research should explore how radiopharmaceutical properties interact with resolution effects and impact the optimization of PVC algorithms.

Looking ahead, AI holds significant promise in addressing the limitations of PVC. For instance, Panin et al. introduced the FastPET network, which uses compressed TOF data as input to reconstruct high-quality PET images without blurring [260]. This approach, referred to as reconstruction-based PVC, represents a much-needed advancement, combining PVC with AI to deliver enhanced resolution and speed. While early results are promising, further research and clinical validation are necessary to fully realize its potential. Similarly, DL methods and diffusion models offer hope for the future, potentially enabling PVC to become a routine clinical tool, tailored to the specific requirements of each task. PVC-specific standardization is still lacking; however, future integration with broader SNMMI/EANM harmonization efforts may provide the framework needed for clinical validation [261]. Meanwhile, recent AI-based approaches have begun to address issues like noise sensitivity and limited generalizability, though further validation remains necessary [61].

Despite these promising efforts, it is important to recognize several outstanding challenges. AI-based PVC methods often require large, diverse, and well-annotated datasets, which are not always available [215]. Moreover, models trained on one type of scanner, population, or protocol may not generalize well to others, leading to performance variability [262]. The “black box” nature of DL also limits interpretability, making it difficult for clinicians and regulators to understand the rationale behind corrections, thereby affecting trust and adoption in clinical settings [263].

To further support our analysis, Figure S1 illustrates a comparative assessment of clinical readiness across 11 common PVC methods based on literature-reported evidence of their deployment, reliability, and feasibility. Additionally, Figure S2 provides a side-by-side comparison of SUV_max_ changes across different PVC techniques in typical brain and oncology imaging settings, highlighting variability in signal recovery and emphasizing the context-specific nature of each method’s effectiveness. These visualizations aim to complement the tabulated summaries by offering a clearer overview of method-specific trends and performance characteristics across modalities.

**Fig. 15:**
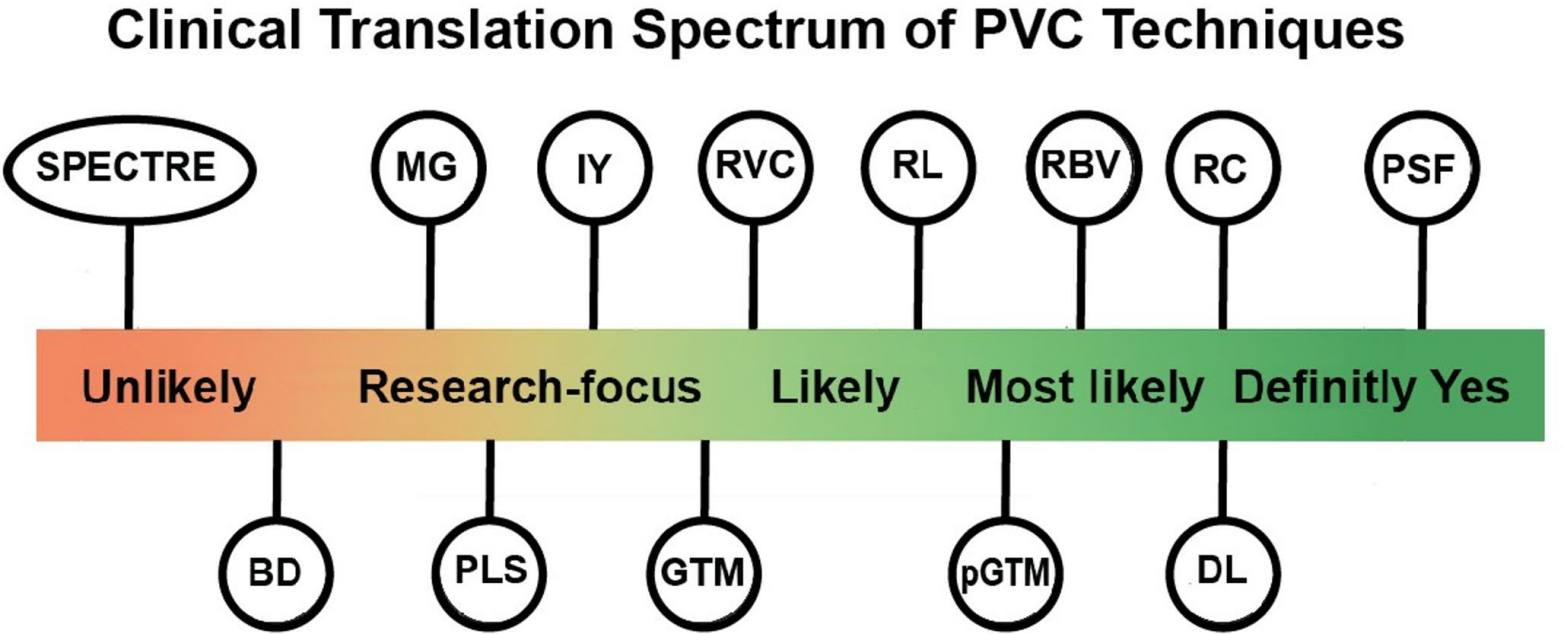
Graphical summary of the clinical readiness of partial volume correction (PVC) methods. PVC methods are positioned along a spectrum from unlikely to definitely yes regarding their likelihood of routine clinical adoption. This figure provides a visual overview to complement the detailed comparative information presented in Table 2

Bridging the gap between research and clinical practice requires a multidisciplinary approach, focusing on the development of robust and efficient PVC methods, simplifying segmentation processes, and validating these techniques on larger, more diverse clinical datasets. Collaboration between imaging experts, clinicians, and machine learning researchers could accelerate the integration of PVC into routine clinical workflows. With these efforts, PVC can evolve into a critical tool for enhancing diagnostic precision and personalizing patient care, paving the way for a more accurate and efficient clinical paradigm. Table 2 provides a comparative overview of commonly used PVC methods in PET and SPECT imaging, including their strengths, limitations, and potential for adoption in clinical workflows, while Fig. 15 graphically summarizes their relative likelihood of routine clinical use.

**Table 2:** Comparative overview of commonly used PVC methods in PET and SPECT, including methodology, strengths, limitations, clinical potential, and implementation resources (open-source tools, pipelines, github repositories)

Method	Open-Source Implementation/Tools	Benefits	Pitfalls	Mainly Applied to (Organ/Tissue)	Will It be Routinely Deployed in the Clinic?
Point Spread Function (PSF)	PETPVC Toolbox	Improves internal signal reconstruction accuracy Reduces need for post-reconstruction corrections	Sensitive to spatial resolution variations Limited in handling complex datasets	Brain, cardiac, oncology (General PET/SPECT)	Definitely yes (already deployed)
Recovery Coefficient (RC)	MIRDPVC	Simple and quick for initial calculations Effective for small and spherical lesions	Typically assumes uniform activity distribution Sensitive to tumour size estimation errors High noise and artifacts	Tumours (oncology PET/SPECT, small lesions, radiopharmaceutical dosimetry)	Most likely yes
Deep Learning-based (DL)	Implemented in specific research studies; not publicly available	Faster processing Advanced noise removal and signal correction Scalable to large datasets	Requires large datasets for training Challenges in generalization to various data types Limited for small regions	Brain, cardiac, oncology (multi-tracer PET/SPECT, General PET/SPECT). emerging applications	Most likely yes
Region-Based Voxelwise (RBV)	PETPVC Toolbox	Reduces extracortical noise Effective for correcting signals in small regions	Overemphasizes cortical signals Limited accuracy in group-level statistical analyses	Brain (cortex/hippocampus, Alzheimer’s, tau/small structures)	Most likely yes
Perturbation-based GTM (pGTM)	Implemented in specific research studies; not publicly available	Reduces Gibbs artifacts Effective in low-resolution systems like SPECT	Requires precise modelling Sensitive to structural misalignments and noise	Heart (SPECT MPI), tumours (oncology SPECT)	Most likely yes
Iterative Deconvolution	PETPVC Toolbox	Effective in reducing spillover and spill-in effects Useful for high-resolution reconstructions	Amplifies noise in dynamic datasets Computationally expensive Limited clinical standardization	Brain, tumours, (General PET/SPECT)	Most likely yes (Research-focused)
Geometric Transfer Matrix (GTM)	PETPVC Toolbox	High accuracy in reducing partial volume effects Suitable for longitudinal studies	Requires precise PET/MRI registration Sensitive to structural changes like brain atrophy Increases noise	Brain (cortical regions, longitudinal neuroimaging, Alzheimer’s)	Most likely yes(Research-focused)
Iterative Yang (IY)	PETPVC Toolbox	Suitable for iterative reconstructions Reduces noise in dynamic images	Computationally intensive Introduces noise and artifacts under certain conditions	Brain (dynamic PET), cardiac (SPECT MPI), small tumours	Unlikely (primarily for research)
Parallel level set (PLS)	Implemented in specific research studies; not publicly available	Better quantification in small cortical regions (e.g., amyloid/tau PET).	Requires high-quality MRI segmentation/registration	Brain (amyloid/tau PET, cortical imaging), small tumours (oncology PET). MRI-guided, mainly brain PET	Unlikely (Primarily research-focused)
Müller-Gärtner (MG)	PETPVC Toolbox	Effective for brain signal correction in Alzheimer’s patients Compatible with longitudinal approaches	Highly dependent on PET/MRI alignment Susceptible to segmentation errors	Brain (Alzheimer’s, cortical imaging, hippocampus)	Unlikely (primarily for research)
Blind Deconvolution (BD)	Implemented in specific research studies; not publicly available	Reduces noise without prior PSF knowledge; improves contrast.	Requires simultaneous PSF estimation; prone to errors with motion artifacts or noisy datasets.	General PET/SPECT, small tumours (research-focused)	Unlikely (primarily for research)
SPECTRE	Implemented in specific research studies; not publicly available	Improves spatial resolution Enhances signal differentiation Effective in oncology imaging	Computationally demanding Susceptible to scatter correction and image misalignment challenges	Tumours (oncology SPECT, bone metastases, radiopharmaceutical therapy dosimetry)	Unlikely (primarily for research)

## Conclusion and future perspectives

Based on the available evidence, PVC can be either beneficial or detrimental depending on the specific task, and we cannot and should not assert its effectiveness categorically across all applications. For instance, while PVC improves quantification in small lesions, it may also amplify noise or introduce segmentation-induced bias and artifacts in complex or heterogeneous regions, thereby reducing interpretability or diagnostic confidence. What is clear, however, is that while various PVC methods exist, they have not yet been widely adopted as practical tools in clinical practice. By “widely adopted”, we refer to their integration into standardized clinical workflows, inclusion in vendor-supported software, and use in multi-centre trials. Moreover, validated software for post-reconstruction PVC methods is not widely adopted by industry, limiting standardization and broader clinical implementation. These methods face numerous challenges, including computational complexity and time consumption, sensitivity to noise, reliance on accurate image registration, lack of generalizability, difficulties with small structures and uptake heterogeneity, advancements in reconstruction-based approaches like PSF, and cost and equipment limitations.

In contrast, reconstruction-based methods, such as PSF modelling, are now widely implemented on modern PET and SPECT systems. These methods address PVEs inherently during the reconstruction process, reducing the need for separate post-reconstruction PVC corrections. While DL-based models have shown promising results, in task-specific image evaluations, there are still significant barriers to the clinical translation of AI tools, and the number of studies in this area remains limited. However, these methods are rapidly evolving, and we are optimistic about their potential to become more effective and support existing approaches in the future. Ultimately, this review synthesizes findings across more than 150 articles and identifies context-specific advantages of PVC techniques across neurological, oncological, and cardiac applications.

As a result, there is an urgent need for large disease-specific datasets, standardized and reproducible PVC approaches, and integration of advanced image enhancement tools into existing clinical workflows. These efforts will be crucial for the successful adoption and clinical implementation of both PVC and AI-driven methods. Finally, while PVC methods have clear potential, their application must be context-aware, evidence-driven, standardized, and reproducible to ensure reliable clinical integration.

## Supplementary Information

Below is the link to the electronic supplementary material.

Supplementary Material 1

## Data Availability

The data used in this work is not available.

## Abbreviations

PET: Positron Emission Tomography
SPECT: Single–Photon Emission Computed Tomography
CT: Computed Tomography
MRI: Magnetic Resonance Imaging
PVE: Partial Volume Effect
PVC: Partial Volume Correction
FWHM: Full Width at Half Maximum
FOV: Field of View
PSF: Point Spread Function
RBV: Region–Based Voxel–wise
GTM: Geometric Transfer Matrix
RC: Recovery Coefficient
SGTM: Simplified Geometric Transfer Matrix
MG: Müller–Gärtner
pGTM: Perturbation–Based Geometric Transfer Matrix
IDM: Iterative Deconvolution Model
IY: Iterative Yang
RL: Richardson–Lucy Deconvolution
RVC: Reblurred Van Cittert
PLS: Parallel Level Set
BD: Blind Deconvolution
SFSRR: Structural–Functional Synergistic Resolution Recovery
STC: Single–Target Correction
TOF: Time–of–Flight
QC: Quality Control
FBP: Filtered Back–Projection
SNR: Signal–to–Noise Ratio
MAE: Mean Absolute Error
RMSE: Root Mean Square Error
MPI: Myocardial Perfusion Imaging
SBR: Specific Binding Ratio
ICC: Intraclass Correlation Coefficient
COV: Coefficient of Variation
SSIM: Structural Similarity Index
PSNR: Peak Signal–to–Noise Ratio
CRC: Contrast Recovery Coefficient
SUV: Standardized Uptake Value
TBR: Tumour–to–Background Ratio
VT: Distribution Volume
BPND: Binding Potential Non–Displaceable
IMBV: Intramyocardial Blood Volume
TLG: Total Lesion Glycolysis
HMR: Heart–to–Mediastinum Ratio
SUR: Specific Uptake Ratio
DL: Deep Learning
AI: Artificial Intelligence
CNN: Convolutional Neural Network
DeepPVC: Deep Learning–Based Partial Volume Correction
CycleGAN: Cycle–Consistent Generative Adversarial Network
FastPET: AI–Based PET Image Reconstruction Model
DL: PVC–Deep Learning–Based Partial Volume Correction
SUVR: Standardized Uptake Value Ratio
^18^F: FDG–Fluorodeoxyglucose, a PET radiotracer
^11^C: PiB–Pittsburgh Compound B, used in amyloid imaging
^18^F: AV1451–Tau PET imaging tracer
^18^F: Flutemetamol–PET tracer for amyloid imaging
^99m^Tc: Technetium–99 m, a common SPECT radiotracer
^177^Lu: Lutetium–177, used in radionuclide therapy
^131^I: Iodine–131, used in thyroid and radiotherapy imaging
^99m^Tc: PYP–Technetium–99 m Pyrophosphate, used in cardiac imaging
^99m^Tc: RBC–Technetium–99 m Red Blood Cell Labelling
^68^Ga: DOTATOC–Gallium–68 DOTATOC, used in neuroendocrine tumour imaging
^18^F: MK–6240–PET tracer for tau pathology
¹¹C: UCB–J–Synaptic Vesicle Glycoprotein 2 A tracer
¹⁸F: NaF–Sodium Fluoride
¹²³I: FP–CIT–DaT tracer
¹²³I: mIBG–Meta–Iodobenzylguanidine
⁹⁹ᵐTc: TRODAT–1–DaT SPECT tracer
¹²⁴I/¹³¹I: CLR1404–Alkylphosphocholine theranostic
¹¹C: FMZ–Flumazenil
¹⁶¹Tb: Terbium–161
²²⁵Ac: Actinium–225
NSCLC: Non–Small Cell Lung Cancer
PSMA: Prostate–Specific Membrane Antigen
HNSCC: Head and Neck Squamous Cell Carcinoma
AD: Alzheimer’s Disease
MCI: Mild Cognitive Impairment
DAT: Dopamine Transporter
MAO: B–Monoamine Oxidase B
LAFOV: Long Axial Field–of–View
OSEM: Ordered Subset Expectation Maximization
HYPR: HighlY constrained backPRojection
ROI: Region of Interest
VOI: Volume of Interest
PTV: Planning Target Volume
BTV: Biological Tumor Volume
RTP: Radiotherapy Planning
ATTR: Transthyretin Amyloidosis
AMAP: S / AMAP–R–Anatomically–guided Maximum A–Posteriori
PETPVC: PET Partial Volume Correction toolbox
APPIAN: Automated Pipeline for PET Image Analysis
HC: Healthy Controls
SNMMI: Society of Nuclear Medicine and Molecular Imaging
EANM: European Association of Nuclear Medicine
EARL: EANM Research Ltd. (Accreditation Program)
