# Supplementary material for: Critical review of partial volume correction methods in PET and SPECT imaging: benefits, pitfalls, challenges, and future outlook
Source: Eur J Nucl Med Mol Imaging. 2025 Nov 5;53(4):2830–61. doi: 10.1007/s00259-025-07612-5 (PMC12920307; doi:10.1007/s00259-025-07612-5)
Supplement: Supplementary file 1 — Supplementary Material 1 [file 259_2025_7612_MOESM1_ESM.pdf]

Supplementary Material

Supplemental Table 1. Summary of studies reporting on reconstruction-based PVC in PET imaging. "W-R" denotes within reconstruction.

**Publication**	**Data details**	**Categories of PVC**	**Algorithm/Architecture**	**Evaluation metrics**
Vunckx et al. (Vunckx *et al.*, 2011)	BrainWeb phantom (PET/MRI)	W-R Anatomical Priors	A-MAP, JE, modified locally JE	Mean absolute bias, % noise
Bousse et al. (Bousse *et al.*, 2012)	Simulated/clinical PET brain data	W-R ROI-based	Markov Random Fields (MRF) with mean field approximation	RC, NMSE, CV, RSTD
Gutierrez et al. (Gutierrez *et al.*, 2012)	18F-FDG brain PET of 19 dementia patients	W-R Anatomical Voxel-based	Comparison of MRI segmentation methods (SPM versions)	Bias, accuracy
Bauer et al. (Bauer *et al.*, 2013)	Over 400 subjects FDG brain PET	W-R Voxel & ROI-based	Normalization regions and PVC methods analysis	Chi square test, T-test, ANOVA test
Sattarivand et al. (Sattarivand *et al.*, 2013)	Brain and sphere phantoms	W-R Region-based (sinogram-space	sGTM, GTM comparison in sinogram space	Mean/SD of RC
Tang and Rahmim(Tang and Rahmim, 2014)	Simulated MRI/PET	W-R	Wavelet JE (WJE)-MAP	Noise vs Bias
Dutta et al. (Dutta *et al.*, 2015)	BrainWeb phantom, human datasets	W-R PET/MRI Joint Entropy	Image deblurring with joint entropy prior	RMSE, mean SUV ratio
Caldeira et al. (Caldeira *et al.*, 2015)	MR-BrainPET, BrainWeb database	W-R Anatomical Priors	OP-OSEM with/without Gaussian filtering	Noise, RMSE, AUC
Novosad and Reader (Novosad and Reader, 2016)	BrainWeb dynamic simulations (18F-FDG)	W-R Kernel Functions	Spectral analysis, kernel temporal functions	RMSE, COV, bias
Turco et al. (Turco *et al.*, 2016)	Myocardial (18F-FDG), XCAT phantom	W-R Edge-preserving	MAP with anatomical priors	RC, CRC, CNR
Hutchcroft et al. (Hutchcroft *et al.*, 2016)	BrainWeb MRI datasets (PET lesion contrast)	W-R	Kernel-based patch MR reconstruction	Lesion RC, SNR
Mehranian et al. (Mehranian *et al.*, 2017a)	BrainWeb PET/MRI datasets	W-R Synergistic PET-MRI	Weighted priors with MR sensitivity	Quant. Error, Noise Reduction
Mehranian et al. (Mehranian *et al.*, 2017c)	BrainWeb Phantom	W-R MR-Guided	Penalized MLAA (P-MLAA+), anato-functional joint entropy and anatomical quadratic penalty functions	Quantification errors, visual quality, noise reduction

Baker et al. (Baker *et al.*, 2017a)	AV-1451 scans (18F, T1 MRI)	W-R Rousset-Based PVC	Spearman correlation for PVE analysis	SUVR, ROI bleed-in
Filipović et al. (Filipović *et al.*, 2018)	Simulated/clinical datasets	W-R Multimodal MR-guided	MRF-guided posterior probability estimation	Variance, Regularization Bias
Belzunce et al. (Belzunce *et al.*, 2018)	BrainWeb (FDG PET)	W-R Resolution Modelling	MAP, MR voxel adjustments	Brain Contrast, Bias
Bland et al. (Bland *et al.*, 2019)	BrainWeb/real FDG data	W-R MR/PET-informed	Kernel, MAP, MR-guided methods, Hybrid Kernel-Anato MAP	SSIM, NRMSE, Bias
Turco et al. (Turco *et al.*, 2019)	In vivo sheep datasets, ex vivo scans	W-R Motion + Edge TV Prior	OSEM ± RR, MAP-TV	Absolute Bias, GT Similarity
Mehranian et al. (Mehranian *et al.*, 2019)	FDG PET/MR phantoms, in vivo datasets	W-R Synergistic PET-MR	Weighted priors, MAP-EM	Quant. Error, Artifacts
Deidda et al. (Deidda *et al.*, 2019)	Torso/Jaszczak phantom, clinical datasets	W-R Hybrid Kernel	Iterative LM-HKEM	CNR, CRC, Bias
Chen et al. (Chen *et al.*, 2019)	PET/MRI (20 patients)	W-R Patch-Based MR Kernel	Matrix formed via MR features for kernel construction	SNR, CV
Raptis et al. (Raptis *et al.*, 2020)	Real PET/MRI data	W-R LR/Rousset	Lucy-Richardson, High-Res Scanner PVC	Activity Recovery, Bias
Sudarshan et al. (Sudarshan *et al.*, 2020)	Simulated PET-MRI datasets, BrainWeb phantom	W-R Joint PET-MRI Prior	MRF, Bayesian priors	RRMSE, SSIM
Kang and Lee(Kang and Lee, 2021)	BrainWeb Simulated/real data	W-R l1-Bowsher	Iterative l1 Norm Weighting	Bias, Small Lesion Intensity
Ibaraki et al. (Ibaraki *et al.*, 2022)	FDG PET Subjects	W-R PSF-based	PSF-OSEM	SUVR, Spatial Resolution
Mandeville et al. (Mandeville *et al.*, 2024)	Simulated/real PET, murine functional data	W-R bsGTM	B-spline GTM, Unbiased PVC Reconstruction	SNR, CNR, BPND, ΔBPND
Mehranian et al. (Mehranian *et al.*, 2017b)	Florbetaben/FDG-PET, 3D brain data	W-R Multi-parametric Priors	Burg Joint Entropy Prior in MAP	Bias-Variance, Feature Preservation
Ahn et al. (Ahn *et al.*, 2013)	2D XCAT liver/lung lesions	W-R CRC-based Penalties	Penalized-likelihood (nonquadratic penalties)	RC (Pre/Post-PVC)
Meechai et al. (Meechai *et al.*, 2015)	FDG PET, phantoms	W-R Super-resolution	Super-resolution (SR) with shifted grids	ARC, percentage yield
Bowen et al. (Bowen *et al.*, 2013)	Dynamic FDG brain PET	W-R RSF + Perturbation GTM	Perturbation GTM PVC with PSF OSEM	Bias (Kinetics, CMRGlc)

Ashrafinia et al. (Ashrafinia *et al.*, 2014)	Simulated lung FDG PET	W-R PSF-based Framework	Iterative EM	Detection Tasks (PWMF/NPWMF)
Irace et al. (Irace *et al.*, 2016)	Breast lesion phantom	W-R Spatial PCA (PSF Estim.)	varRM, DIRECT, Deconvolution Methods	%BIAS, FOV-SD, RMSE
Cysouw et al. (Cysouw *et al.*, 2016)	FDG PET/CT for Small Tumors studies	W-R Resolution Modelling	LR PVC, Masked Spillover Methods	Accuracy, precision, recovery coefficient, bias, activity concentration ratios.
Ashrafinia et al. (Ashrafinia *et al.*, 2017)	Simulated dynamic liver tumors (XCAT)	W-R PSF Modelling	OSEM + Analytical PSF Models .	CRC, Noise-Bias, SUV_{mean} bias, SUV_{max} bias.
Xu et al. (Xu *et al.*, 2015)	PET/CT (NEMA Phantom)	W-R Iterative Shape Consist	Iterative Shape/Denoising Weighted PVC	Structure Integrity, PVE Assessment
Wu et al. (Wu *et al.*, 2021)	Phantom PET (Fluoride) + Clinical Nodules	W-R Bayesian Likelihood	Q.Clear (PSF/TOF Bayesian), OSEM Models	RC, CR, SUV_{max}, SUV_{mean}, and $\%\Delta SUV_{mean}$
Turco et al. (Turco *et al.*, 2020)	Ex vivo ovine hearts	W-R Edge + Anatomy Priors	MAP + RR, TV Regularization	Absolute quantification, relative tracer distribution
Zhao et al. (Zhao *et al.*, 2023)	Na Phantom + Mouse FDG PET	W-R PSF-OSEM Modelling	Optimized PSF-OSEM, Deformed Models	FWHM, CNR, CRhot

Supplemental Table 2. Summary of studies reporting on post-reconstruction-based PVC in PET imaging. "W-P" refers to within post-reconstruction

**Publication**	**Data details**	**Categories of PVC**	**Algorithm/Architecture**	**Evaluation metrics**
Lehnert et al. (Lehnert *et al.*, 2012)	Simulated 11C-raclopride rat brain PET, Monte Carlo simulation	W-P GTM method	Evaluating PVE on TACs and binding potential	RMSE for TACs, BPND stability
Du et al. (Du *et al.*, 2013)	Dog cardiac perfusion PET data, simulation studies	W-P Image-based	Spill-in/spill-out correction for cardiac PET	LV wall uniformity, quantitative accuracy
Wang and Fei(Wang and Fei, 2012)	simulated PET data	W-P Voxel-based	Bayesian deconvolution with edge-preserving constraints	Bias, COV, recovery ratio, recovery error
Yan et al. (Yan *et al.*, 2015)	Simulated/clinical FDG brain PET	W-P Voxel-based	MRI-guided PET filtering method	MSE, PSNR, SSIM, bias, COV
Gao et al. (Gao *et al.*, 2021)	BrainWeb phantom, in vivo human PET (BLSA)	W-P Voxel-based	Least squares with MRI-guided NLM (NLMA) regularization	Bias-noise tradeoff, visual improvement
Zhu et al. (Zhu *et al.*, 2021)	BrainWeb phantom, in vivo PET-MR	W-P MR-guided	Deconvolution with parallel level set (PLS) regularization	Amyloid quantification, quantitative performance
Zhu et al. (Zhu *et al.*, 2019)	BrainWeb phantom	W-P Subtle MR guidance	Comparison of NLMA and SBPLS for MR-guided PVC	Bias, COV
Gallivanone et al. (Gallivanone *et al.*, 2012)	49 patients Gastric cancer 18F-FDG PET-CT	W-P RC- based	PVC-SUVBW correlation with histology data	Statistical analysis (Kruskal-Wallis, Mann-Whitney) and k-means
Gallivanone et al. (Gallivanone *et al.*, 2014)	Breast cancer PET (40 patients)	W-P Region and Voxel based	Pretherapy PET PVC-SUVBW correlation with histology	ER/PgR, hormone receptor, Mib-1 proliferation
Picchio et al. (Picchio *et al.*, 2014)	Head/neck cancer PET-CT (19 patients)	W-P Region-based	SUV_{mean} corrected with visual evaluation	Survival prediction, FDG uptake parameters
Gargouri et al. (Gargouri *et al.*, 2018)	PET/CT data	W-P Various approaches	Lucy-Richardson deconvolution with block shrink denoising	SUV measurements
Taghvaei et al. (Taghvaei *et al.*, 2018)	Refractory NHL PET-CT (17 patients)	W-P Region-based	Adaptive contrast-oriented thresholding	SUV_{max}, TLG, pvcTLG
Baun et al. (Baun *et al.*, 2018)	Breast cancer recurrence PET (102 patients)	W-P Region-based	ROVER™ software	SUV_{max}, SUV_{mean}, $cSUV_{mean}$
Sattarivand et al. (Sattarivand *et al.*, 2012)	3D digital and physical phantoms	W-P Region-based	sGTM: Enhanced GTM with spill-over corrections	Accuracy, precision, robustness against mis-registration, and PSF estimate errors

McGinnity et al. (McGinnity *et al.*, 2013)	[11C]DPN PET paired datasets	W-P SFS-RR-Based	Structural-Functional Synergistic (SFS-RR)	Global/Regional [11C]DPN VT
Evans et al. (Evans *et al.*, 2015)	12 mice injected with 18F-FDG, 6 treated for MI.	W-P Region-based	MRI segmentation; GTM PVC	IDIF extraction improvement, Ki values
Funck et al. (Funck *et al.*, 2014)	simulated and clinical PET datasets.	W-P	idSURF algorithm with MRI gray matter surface	Accuracy, precision, MRSE
Coello et al. (Coello *et al.*, 2013)	Synthetic/clinical 18F-FDG PET with MR volumes	W-P Voxel-based	LoReAn algorithm vs. GTM	Bias, STD, COV, Pearson's correlation coefficient
Kim et al. (Kim *et al.*, 2013)	Eight healthy volunteers using [18F]DOPA PET.	W-P Region-based	SFS-RR vs. GTM	ICC, variability, Ki cer recovery
Pandey et al. (Pandey *et al.*, 2012)	Phantom and 42 lung nodules in 5 patients	W-P RC-Based	Spreadsheet for SUV calculation	Recovery coefficient, background ratios
Muellauer et al. (Muellauer *et al.*, 2013)	Micro-hollow sphere phantom, isotopes: 68Ga, 18F, and 11C	W-P RC/CRC-Based, VOI based methods		RCs, and CRCs
Reeps et al. (Reeps *et al.*, 2013)	23 aortic aneurysm patients, 18F-FDG PET/CT	W-P Geometrical model-based correction	IDL-based software, histopathology correlation	SUVs vs. macrophages, MMP-9 expression
Torigian et al. (Torigian *et al.*, 2013)	49 subjects with emphysema	W-P Region-based	Image segmentation in 3DVIEWNIX	E volume vs. $CSUV_{max}$, $CCSUV_{max}$
Wang et al. (Wang *et al.*, 2015)	142 lymph nodes in 71 NPC FDG PET/CT patients	W-P RC-Based	Sphere-to-background ratio.	Sensitivity, accuracy for small nodes
Gallivanone et al. (Gallivanone *et al.*, 2013)	137 oncological patients; PET	W-P RC-based	Threshold isocontour	Accuracy, radioactivity recovery
Boivin et al. (Boivin *et al.*, 2014)	17 neurodegenerative patients	W-P Voxel/VOI-Based	Five different PVC methods including Alfano, Muller-Gartner, Meltzer, Shidahara, and Rousset's techniques	Gray/white matter activity changes
Giganti et al. (Giganti *et al.*, 2014)	Gastric adenocarcinoma DW-MRI and 18F-FDG-PET/CT before and after neoadjuvant therapy	W-P Region-based	RC-based PVC	ADC/PVC-SUV correlation, TRG accuracy
Grecchi et al. (Grecchi *et al.*, 2015)	Phantom, metastatic bone 18F-fluoride PET/CT, prostate/breast cancer	W-P Multimodal PVC using SFS-RR	SFS-RR using wavelet transform	Bias reduction, SUV recovery
Ortega et al. (Ortega *et al.*, 2020)	75 men, 232 metastatic nodes < 12 mm, PET/CT data	W-P	Smoothing filter, PROMISE miPSMA scoring	SUV_{max}/SUV_{mean} before/after PVC

Lue et al. (Lue *et al.*, 2014)	11 patients with Parkinson's disease and 6 healthy subjects	W-P RC-based	VOI + large VOI techniques	SOR before/after PVC
Li et al. (Li *et al.*, 2019)	14 epilepsy patients and 19 controls, PET/MR data	W-P MRI-based	SPM analysis comparison before and after PVC	Epilepsy detection rate before/after PVC
Raptis et al. (Raptis *et al.*, 2019)	High-resolution PET data, HRRT simulation	W-P Voxel-based	Lucy-Richardson deconvolution, Rousset PVC	Bias, region-specific bias
Greve et al. (Greve *et al.*, 2014)	Dynamic PET scans of serotonin-4 receptor in 16 healthy subjects	W-P Voxel based	Muller-Gartner PVC, surface-based smoothing	Bias, variance in BPND estimates
O'Keefe, et al. (Jones *et al.*, 2014)	30 Flutemetamol-PET subjects with MRI/CT-based PVC	W-P Region-based	2- and 3-compartment PVC methods	Accuracy comparison, mean correlation coefficients
Bural et al. (Bural *et al.*, 2015)	Four patients with normal brain MRI and FDG-PET scans	W-P Voxel-based	MRI segmentation for PVC	SUV for grey matter after PVC; reduction of discrepancy between CSF compartments.
Salavati et al. (Salavati *et al.*, 2015)	106 lesions from 55 lung cancer patient FDG-PET/CT.	W-P Region based	Adaptive thresholding, lesion-based PVC	Respiratory-gated vs non-gated SUV/TLG comparison
Niesporek et al. (Niesporek *et al.*, 2015)	Phantom and (23)Na-MRI volunteer study	W-P Region-based	GTM-method	Correction performance, CSF discrepancy reduction
Schwarz et al. (Schwarz *et al.*, 2019)	Mayo Clinic aging and AD studies, longitudinal PiB amyloid PET	W-P Region-based	GTM, 2- and 3-compartment models	Relative precision, longitudinal measurement variability
Rausch et al. (Rausch *et al.*, 2022)	[68Ga]Ga-pentixafor PET/MR for carotid plaques	W-P Voxel-based	Impact of PVC on MR-AC and uptake	Mean/max TBR, SUV changes
Cal-González et al. (Cal-González *et al.*, 2017)	Simulated/phantom calcified plaques; pilot NaF-PET studies	W-P Region-based	STIR MoCo-based reconstruction	Increased lesion-to-background ratios (LBR)
Turco et al. (Turco *et al.*, 2015)	XCAT thorax simulation with left ventricle lesions	W-P Anatomy-based	Edge-preserving priors, anatomy-based reconstructions	RC, CRC, CNR
Greve et al. (Greve *et al.*, 2016)	99 subjects, Harvard Aging Brain study.	W-P Region-based	SGTM, MZ, MG, NoPVC	isthmus cingulate (IC), t-test, Bias, p-value
Thomas et al. (Thomas *et al.*, 2016)	Simulated brain PET for demonstrating PETPVC toolbox	W-P Mixed (Multiple PVC Methods)	PETPVC toolbox comprising eight core PVC techniques	GM recovery, PSF mismatch sensitivity
Sasaki et al. (Sasaki *et al.*, 2016)	Nine AD patients and 11 cognitively normal controls	W-P CT-based PVC for amyloid PET imaging	Comparison of CT-based PVC to MRI-based PVC.	SUVR values, constant multiplication impact

	using 11C-PiB-PET/CT and MRI scans.			
Yang et al. (Yang *et al.*, 2017)	97 NC, 96 EMCI, 129 LMCI, and 91 AD subjects	W-P Joint-entropy-based PVC technique	Gradient projection with non-negativity	Enhanced brain network analysis, AD classification
Shigemoto et al. (Shigemoto *et al.*, 2018)	18 amyloid-positive early AD patients and 36 controls	W-P Region-based	PETPVE12 toolbox	Tau deposits vs atrophy discrepancy
Lu et al. (Lu *et al.*, 2021)	17 AD, 11 CN participants using 11C-UCB-J PET	W-P Voxel-Based	Comparison of MG and IY PVC methods	VT, BPND, K1, and volume differences before/after PVC.
Su et al. (Su *et al.*, 2015)	Simulated/human PiB amyloid imaging	W-P (RSF)-Based	RSF PVC technique	PiB retention changes, simulated accuracy
Schwarz et al. (Schwarz *et al.*, 2017)	129 Mayo Clinic subjects, serial PiB and T1-MRI scans	W-P Two-class PVC in voxel space	Linear mixed-effects regression model	MMSE score correlation, plausibility, accumulator status prediction
Matsubara et al. (Matsubara *et al.*, 2020)	[11C]PiB PET, 92 ADNI participants (16 HC, 58 MCI, 18 AD)	W-P MR -guided PVC	PoR framework iterating PVC and registration for accuracy	PV-corrected SUV, SUVR, intra-region CoV
Minhas et al. (Minhas *et al.*, 2018)	9 subjects with [C-11]PiB PET & MR, postmortem assessments	W-P Region-based	Meltzer, modified Müller-Gärtner, RBV	Correlations between PiB SUVRs & Aβ load
Sur et al. (Sur *et al.*, 2022)	18F-flutemetamol PET scans in AD patients from EPOCH trial	W-P Region-based	Assessment of MZ, SGTM for amyloid PET	Correlation between PVC vs. uncorrected SUVr values, treatment effect eval.
Gao et al. (Gao *et al.*, 2018)	Amyloid PET phantom simulating patient data (11C-PIB PET/MRI)	W-P Voxel-based PVC	Weighted least squares with NLM regularization, OSL optimization	Bias-noise tradeoff, performance in AD-relevant regions
Costoya-Sánchez et al. (Costoya-Sánchez *et al.*, 2024)	247 subjects from the AD Neuroimaging Initiative	W-P Region-Based	FTP SUVR values and longitudinal rates of change calculation for various ROIs	RCs for PVC impact assessment
Malpas et al. (Malpas *et al.*, 2015)	115 subjects (55 HC, 53 MCI, 7 AD) with FDG-PET and T1 MRI over 24 months	W-P Longitudinal PVC in AD.	PVElab using Müller-Gärtner PVC	Increased uptake w/PVC, correlation w/structural atrophy
Matsubara et al. (Matsubara *et al.*, 2016)	5 HC, 8 AD patients with [11C]PiB PET	W-P Region-based and voxel-based PVC.	GTM for region-based	BPND, cortical value changes

Eldib et al. (Eldib *et al.*, 2016)	ACR phantom (8 GBq of Y-90 solution, 10:1 contrast)	W-P MR-Guided based PVC	Wavelet decomposition approach	Contrast recovery improved by 13%
Kaida et al. (Kaida *et al.*, 2020)	108 NSCLC patients, dual-point 18F-FDG PET imaging	W-P RC-based	Tumor diameter-dependent RC	OS, DFS via Cox regression, Glut-1 expression
Lee et al. (Lee *et al.*, 2016)	38 women w/early invasive breast cancer, 18F-FDG PET	W-P RC-Based PVC for SUV_{max}	Multivariate logistic regression	High PVC-SUV_{max} association w/Oncotype DX RS
Grecchi et al. (Grecchi *et al.*, 2017)	[11C]PIB PET & T1/T2-weighted MR of 8 HC, 20 MS patients	W-P Voxel-based PVC	Wavelet-based SFS-RR	Improved WM/lesion contrast
Shidahara et al. (Shidahara *et al.*, 2017)	1 HC, 1 AD subject w/[18F]THK5351, [11C]PIB PET	W-P Multi-Method	Different PVC methods applied with FreeSurfer segmented anatomical information	Variability in SUVRs across different PVC methods and tracer types
Oyama et al. (Oyama *et al.*, 2020)	Simulated brain PET (healthy, AD)	W-P Multi-Method	Comparison of seven PVC algorithms under simulated error conditions.	Error propagation analysis; %diff for corrected SUVR
Sari et al. (Sari *et al.*, 2017)	19 subjects for CMROG estimation	W-P Voxel-Based	Single-Target Correction (STC) method	CMROG diff.
Baker et al. (Baker *et al.*, 2017b)	10 YC, 83 OC, 68 MCI/AD	W-P Region-based-PVC	GTM for inter-regional linear interaction	mean and standard deviation of SUVR
Fazio et al. (Fazio *et al.*, 2017)	40 PET studies using 18F-MNI-659 and 11C-raclopride	W-P Region-based PVC	GTM for linear interaction among regions	Binding potential (BPND)
Wolters et al. (Wolters *et al.*, 2018)	20 [18F]flortaucipir dynamic PET scans	W-P Iterative Deconvolution PVC	Van Cittert iterative deconvolution (IDM) with HYPR	Correlation between hippocampal & CP VT
López-González et al. (López-González *et al.*, 2022)	309 amyloid-β-negative subjects, simulated FTP images	W-P Multi-Compartment PVC	Employed PVC using the RBV method and dedicated compartments for specific regions.	Spill-in effects using Correlation coefficients (R2) and slopes
Sanabria Bohórquez et al. (Sanabria Bohórquez *et al.*, 2024)	Longitudinal tau [18F]GTP1 PET from NHS (CU & AD) and a semorinemab trial.	W-P Voxel-Based	Van Cittert, 2/3-compartment, region-based GTM, LMEM analysis	%ΔSUVR, within-subject variability, LMEM repeatability
van Aalst et al. (van Aalst *et al.*, 2022)	94 healthy subjects, simultaneous FDG-PET/MR with DTI	W-P Region-based PVC	Assessment of age-related changes in glucose metabolism and white matter integrity	Associations in metabolism/atrophy/WM

Funck et al. (Funck *et al.*, 2018)	Monte Carlo PET simulation (SORTEO)	W-P Multi-Method PVC	idSURF, PETPVC	PVC accuracy, voxel quantification
Smith et al. (Smith *et al.*, 2019)	D2-like radioligands (18F-Fallypride, 11C-FLB457); different PET camera resolutions	W-P Region-based PVC	VOI-based PVC using GTM	BPND values before/after PVC, adult age differences
Ferraro et al. (Ferraro *et al.*, 2022)	Analyzed PET and MRI data from 15 ALS patients.	W-P Multi-Method PVC	Meltzer, MG, SGTM	Regression analysis with age and disease duration
Scott et al. (Scott *et al.*, 2024)	343 controls, 55 MCI patients	W-P Region-Based PVC	GTM with Gaussian kernel for PVE simulation	FTP signal, effect of sex on PVC-corrected metrics
Paranjpe et al. (Paranjpe *et al.*, 2019)	Longitudinal study, 48 MCI patients (ApoE ε4 carriers vs non-carriers)	W-P Multi-Method PVC	Reblurred Van Cittert method	Longitudinal FDG SUVR changes
Hellem et al. (Hellem *et al.*, 2021)	21 HDGECs and 17 controls, hybrid 2-[18F]FDG PET/MRI	W-P Region-Based PVC	SGTM method	Correlation with disease burden (CAPS)
Cysouw et al. (Cysouw *et al.*, 2019)	10 NSCLC patients, dynamic 18F-fluorothymidine PET-CT	W-P Parametric PVC	Iterative deconvolution with/without HYPR	Correlation of simplified metrics (SUV/TBR) with VT
Teipel et al. (Teipel *et al.*, 2021)	600 ADNI subjects, 43 INSIGHT-preAD participants	W-P Multi-Compartment PVC	PVC-3 explicitly models WM signal spill-in	CSF Aβ42 levels vs PET sensitivity for amyloid detection
Laymon et al. (Laymon *et al.*, 2021)	29 subjects with [11C]PiB PET and T1 MRI	W-P Region-Based PVC	GTM with PSF specification	SUV impact on amyloid status classification
Ayubcha et al. (Ayubcha *et al.*, 2021)	24 male patients, NaF-PET/CT and MRI	W-P MR-Based PVC	Evaluation of NaF uptake in the femoral neck with PVC	Correlation with age, body weight, SUV_{mean} with/without PVC
Zadeh et al. (Zadeh *et al.*, 2022)	57 HNSCC patients, 18F-FDG-PET/CT scans	W-P Automated PVC	Cox proportional hazards regression for PFS prediction	Comparison of conventional and PVC PET metrics for PFS
GUO et al. (GUO *et al.*, 2022)	21 prostate cancer patients, 68Ga-PSMA PET/CT	W-P Region-based-PVC	BSREMA with different β values	RC, CR, BV, SUV, SNR
Mertens et al. (Mertens *et al.*, 2022)	[18F]MK-6240 PET-MR imaging in aMCI and HC	W-P Multi-Method PVC	Correction for extracerebral spill-in-effects	Group SUVR differences, correlations to clinical parameters
Kafkaletos et al. (Kafkaletos *et al.*, 2024a)	49 HNSCC patients, clinical FMISO PET/CT pre and during RT, phantom RC modeling.	W-P- Region-Based	RC-based PVC tailored for FMISO, iterative SUV segmentation	HTV, SUV, Dice Coefficient, Hausdorff Distance

Kafkaletos et al. (Kafkaletos *et al.*, 2024b)	Same HNSCC cohort, hypoxia quantification using FMISO PET/CT.	W-P- Region-Based	RC-based PVC for pO2 thresholds, tumor-to-muscle ratio segmentation (TMR)	HTVpO2, Dice Coefficient, Hausdorff Distance
Danesh et al. (Danesh *et al.*, 2024)	100 CT images (OASIS-3), labeled via MR; DCNN segmentation.	W-P-Voxel-based	DCNNs (UNet, VNet, SegResNet) on MONAI with attention-based training	Dice Coefficient (DSC), IoU
Dumouchel et al. (Dumouchel *et al.*, 2012)	Simulated and physical LV phantoms, cardiac FDG mouse PET	W-P Model-Based PVC	Convolution of LV image estimate with a 3D PSF described by a five-parameter profile	Recovery percentage, image uniformity
Hofheinz et al. (Hofheinz *et al.*, 2012)	37 simulated tumors from real data.	W-P Model-Free PVC	Implemented a two-step automated PVC algorithm.	Compared mean deviation (± s.d.) with true values.
Hatt et al. (Hatt *et al.*, 2012)	50 esophageal cancer patients	W-P Iterative Deconvolution PVC	Wavelet-based noise reduction, FLAB tumor delineation	Entropy, homogeneity, size-zone variability
Hatt et al. (Hatt *et al.*, 2013b)	50 esophageal cancer patients, PET data	W-P Iterative Deconvolution PVC	FLAB, wavelet-based PVC	Predictive heterogeneity parameters
Hatt et al. (Hatt *et al.*, 2013c)	28 LARC patients, pre/post-treatment PET data	W-P Iterative deconvolution PVC	Lucy-Richardson deconvolution with wavelet-based denoising	Predictive value for tumor response
Hatt et al. (Hatt *et al.*, 2013a)	50 patients with esophageal cancer	W-P Volume-Dependent PVC	Iterative deconvolution and wavelet-based noise reduction for PVC	Sensitivity of parameters to volume definition and PVC
Stefano et al. (Stefano *et al.*, 2014)	29 breast cancer patients with bone lesions	W-PRC model	PVC based on Recovery Coefficients and automatic metabolic volume measurement	Increase in average SUV values with PVC
Mikasa et al. (Mikasa *et al.*, 2015)	113 lesions from 96 breast cancer patients	W-P Multi-Method PVC	PSF, GTM, and background correction	Reduction in uptake overestimation near high activity regions
Golla et al. (Golla *et al.*, 2017)	Hoffman phantom, [11C]flumazenil and [11C]PIB scans	W-P Iterative Deconvolution	PVC with iterative deconvolution combined with HYPR denoising	Improved quantitative accuracy of measured activity concentrations
Akerele et al. (Akerele *et al.*, 2017)	Simulated/phantom data	W-P Multi-Method PVC	PSF, GTM, background correction	Improved lesion quantification and visibility with reduced bias
Arakawa et al. (Arakawa *et al.*, 2017)	[11C]-l-deprenyl-D2 scans on 6 subjects	W-P Voxel-based PVC	Gray matter PET algorithm determined by MRI	%Difference, ICC

Supplemental Table 3. Summary studies reporting on hybrid and AI-based PVC in PET imaging. W-H" represents within hybrid and AI methods.

Publication	Data details	Categories of PVC	Algorithm/Architecture	Evaluation metrics
Xu et al. (Xu *et al.*, 2018)	Phantom, clinical, and pre-clinical PET scans	W-H - Voxel-Based PVC	AP-based iterative clustering for segmentation; iterative voxel-based correction for PVC	SNR, RC
Song et al. (Song *et al.*, 2018)	High-res T1 MR, BrainWeb simulated brain dataset	W-H - Resolution Recovery	Very Deep Super-Resolution Convolutional Neural Network with high-resolution anatomical information for deblurring	PSNR, SSIM
Zhao et al. (Zhao *et al.*, 2019)	115 ADNI participants; 18F-AV1451 PET + structural MR	W-H - Voxel-Based PVC	Voxelwise PVC with reblurred Van Cittert iteration method	SUVR, MMSE, CSF biomarker correlation
Corda-D'Incan et al. (Corda-D'Incan *et al.*, 2020)	BrainWeb PET-MR phantom data	W-H - Synergistic PET-MR Reconstruction	Syn-Net: interconnected unrolled regularised model-based PET-MR reconstruction	MSE, NRMSE
Matsubara et al. (Matsubara *et al.*, 2022)	192 ADNI participants; T1 MR + [11C]PiB PET	W-H - DL Prediction	2D U-Net model for PV-corrected maps	SSIM, ICC
Corda-D'Incan et al. (Corda-D’Incan *et al.*, 2022)	Simulated PET from segmented MR images	W-H - Synergistic PET-MR Reconstruction	FBSEM-Net with residual CNN for anato-functional guidance	MSE
Campanioni et al. (Campanioni *et al.*, 2023)	532 subjects from the OASIS-3 database	W-H - RSF-Based PVC	PVC with RSF; t-SNE for manifold learning	t-SNE metrics
Liu et al. (Liu *et al.*, 2023)	Simulated and clinical PET datasets	W-H - DL-Based Unsupervised PVC	Conditional DIP with MR guidance; iterative deconvolution	PSNR, SSIM
Marsh et al. (Marsh *et al.*, 2023)	HNC xenograft mice; CLR 124/131 for PET/CT	W-H - Novel Preclinical PVC	131I-CLR1404 dosimetry estimation	Uptake measures, tumor dosimetry, tumor growth delay
Farag et al. (Farag *et al.*, 2023)	NEMA phantom, 32 PCa biopsy-confirmed patients	W-H - MR-guided PET reconstruction using CNN	Bowsher-CNN methods for MR-guided PET reconstruction	CR, BV, SNR, SUV, qualitative image assessment
Corda-D'Incan et al. (Corda-D’Incan *et al.*, 2023)	Simulated PET-MR datasets	W-H - Joint PET-MR Reconstruction	Deep learned Joint regularization step linking PET and MR algorithms	NRMSE, feature preservation gains
Shah et al. (Shah *et al.*, 2024)	Imaging from ADNI, OASIS-3, Centiloid; synthetic data, MRI guidance.	W-H- Voxel-Based	Latent Diffusion Model for Resolution Recovery (LDM-RR)	RC, Annualized SUVR change, SSIM, Sample Size estimation

Jomaa et al. (Jomaa *et al.*, 2018)	Cylinder phantom with 18F-FDG spheres	W-H - Deconvolution-Based PVC	Lucy-Richardson with shearlet denoising	RC, SNR
Marsh et al. (Marsh *et al.*, 2022)	Mice HNC xenograft models; CLR124/131 PET-CT	W-H - Preclinical PVC	Theranostic dosimetry based on 124I-CLR1404	Uptake measures, tumor dosimetry, tumor growth delay
Sanaat et al. (Sanaat *et al.*, 2023)	212 clinical PET scans (various tracers)	W-H - DL-Based PVC	CycleGAN for mapping from non-PVC to PVC	SSIM, RMSE, PSNR, voxel-wise and region-wise correlation
Azimi et al. (Azimi *et al.*, 2024a)	160 brain 18F-FDG PET/CT	W-H - DL Framework for FD+PVC Prediction	Encoder-decoder U-Net	PSNR, RMSE, SSIM, relative bias, and absolute relative bias
Azimi et al. (Azimi *et al.*, 2024c)	160 brain 18F-FDG PET/CT	W-H - DL-Based PVC Prediction	Attention-based CNN (ATB-Net)	PSNR, SSIM, RMSE, mean absolute relative SUV difference, mean absolute relative bias

Supplemental Table 4. Summary studies reporting on reconstruction-based PVC in SPECT imageing. "W-R" denotes within reconstruction

**Publication**	**Data details**	**Categories of PVC**	**Algorithm/Architecture**	**Evaluation metrics**
Erlandsson et al. (Erlandsson and Hutton, 2010)	Simulated SPECT data with elliptical phantom and anthropomorphic brain phantom	W-R- Distance-Dependent Blurring Correction	p-PVC combined with FBP	Recovery value for spheres, mean background COV
Erlandsson et al. (Erlandsson *et al.*, 2011)	Brain SPECT studies with DATSCAN tracer, segmented MRI into 33 anatomical regions	W-R- Anatomical-Guided Reconstruction	OSEM with projection domain PVC	Striatal contrast and regional variability (CoV)
Chan et al. (Chan *et al.*, 2013)	Simulated SPECT and dog study with 99mTc-tetrofosmin and 99mTc-RBC tracers	W-R- Noise Suppression-Based PVC	Voxel-based PVC with AMAP and MTC	Quantification, noise level, visual quality
Liu et al. (Liu *et al.*, 2015)	Simulated cardiovascular SPECT and in vivo cardiac imaging with 99mTc tracers	W-R- Anatomical-Guided PVC	pGTM, MTC, iterative approaches	Image quality, quantitative accuracy, reproducibility
Chan et al. (Chan *et al.*, 2016)	Simulated myocardial perfusion and RBC scans, canine blood pool study	W-R- Noise Suppressed PVC	AMAP with Bowsher's prior, sequential Yang's method for PVC	Quantitative assessment, visual image quality, reproducibility
Kangasmaa et al. (Kangasmaa *et al.*, 2021)	Clinical patient studies with artificial lesions, 20 real clinical patients	W-R- Anatomically Guided Reconstruction	Bayesian reconstruction methods (RDP, AMAP-S, AMAP-R)	Error in lesion SUVs, lesion detectability
Marquis et al. (Marquis *et al.*, 2021)	Dual 68Ga/177Lu IEC phantom study and clinical example using 64Cu/67Cu	W-R- Hybrid Reconstruction	HKEM algorithm	Accuracy, recovery, noise amplification, artefact reduction
Vuohijoki et al. (Vuohijoki *et al.*, 2023)	Artificial lesions added to normal bone SPECT/CT studies	W-R- Anatomically Guided Reconstruction	AMAP and KEM algorithms	Lesion SUV error, ROC area
Morphis et al. (Morphis *et al.*, 2016)	Phantom studies with Tc-99m and Au-198, one clinical patient	W-R- Monte Carlo Simulations	Calibration factor incorporated with Monte Carlo-based PVC	Activity quantification accuracy
Leube et al. (Leube *et al.*, 2024a)	Monte Carlo 177Lu SPECT/CT for 720 NEMA phantom configs; 6 physical measurements	W-R- Monte Carlo Simulations	OSEM with/without resolution recovery (CASToR & STIR frameworks)	RC, RC variation (ϑRC), R^2 goodness-of-fit

Supplemental Table 5. Summary of studies reporting on post-reconstruction-based PVC in SPECT imaging. "W-P" refers to within post-reconstruction

**Publication**	**Data details**	**Categories of PVC**	**Algorithm/Architecture**	**Evaluation metrics**
Liu et al. (Liu *et al.*, 2017)	8 canine 99mTc-labeled red blood cell SPECT/CT datasets	W-P- Region-based-PVC	Multi-atlas segmentation with novel label fusion algorithm	Dice similarity coefficient
Wu et al. (Wu *et al.*, 2017)	123I-metaiodobenzylguanidine (123I-mIBG) SPECT/CT imaging of NCAT phantoms and a physical cardiac phantom	W-P- Voxel-based PVC	Iterative blind deconvolution (BD) algorithm with anatomical-based filtering	Relative bias, SNR, HMRs
Ito et al. (Ito *et al.*, 2020)	Striatal phantom for evaluating tracer accumulations	W-P- Region-based-PVC	Novel semi-quantitative approach examining approximate images	Correlation with count density, SBR, CPR
Ren et al. (Ren *et al.*, 2021)	37 patients suspected of ATTR-CM, phantom studies for determining ICF and PVC factors	W-P- Region-based-PVC	Adjustable PVC factors integrated in quantitative SPECT analysis	SUV, reproducibility
Ito et al. (Ito *et al.*, 2022)	Striatal phantom for evaluating tracer accumulations	W-P- Region-based-PVC	Novel semi-quantitative approach examining approximate images	Correlation coefficients
Mohy-ud-Din et al. (Mohy-ud-Din *et al.*, 2018)	Dual-gated myocardial SPECT/CT imaging of 99mTc-RBCs in 12 canines	W-P- Region-based-PVC	Multiple correction schemes including PVC	IMBV estimation, ΔIMBV, correlation with ex vivo measures
Furuta et al. (Furuta *et al.*, 2018)	3D-striatum digital brain (3D-SDB) phantom, various correction methods	W-P- Voxel-based PVC	3D-iterative reconstruction algorithm with multiple corrections	SBR and SUR quantitation accuracy
Liu et al. (Liu *et al.*, 2024)	Geometrical phantom (5 spheres), 40 XCAT phantoms, 10 patient 177Lu-PSMA SPECT.	W-P- Multi-based PVC	RC-PVC, Reblurred Van-Cittert (RVC), Iterative Yang (IY)	RC, MAE, CoV, Mean Activity Differences (kidneys/tumors)
Yin et al. (Yin *et al.*, 2014)	84 healthy adults with -TRODAT-1 SPECT and MRI scans	W-P- Region-based-PVC	Comparison of six ROI methods and three PVC methods (VC, GTM, RBV)	Differences in dopamine transporter (DAT) availabilities, effect on age-related declines

Pourmoghaddas and Wells (Pourmoghaddas and Wells, 2016)	Phantom experiments with 99mTc-SPECT acquisitions	W-P- Region-based-PVC	Quantitative measurements with AC, SC, and PVC on a CZT-detector-based gamma camera	Mean error in absolute activity measurement, lesion contrast
Yin and Chiu (Yin and Chiu, 2017)	20 digital phantom simulations, clinical data from 84 healthy adults	W-P- Voxel-based PVC	Regularized geometric-transfer matrix method (RGTM)	Comparison with VC, GTM, and RBV methods in both constant and non-constant ROIs
Finocchiaro et al. (Finocchiaro *et al.*, 2019)	Two phantoms with 177Lu solution, one with spherical and one with organ-shaped inserts. Additional phantoms for comparison.	W-P- Region-based-PVC	Recovery Coefficients based PVC	Accuracy
Tran-Gia et al. (Tran-Gia *et al.*, 2020)	3D printed 2-compartment kidney phantom with different activity concentrations for SPECT/CT imaging.	W-P- Voxel-based PVC	PETPVC tool for partial-volume correction	Absorbed dose distribution discrepancies
Ramonaheng et al. (Ramonaheng *et al.*, 2021)	Spheres and kidney model in cylindrical, torso, and patient phantoms	W-P- Region-based-PVC	Monte Carlo simulations with sphere-CF and cylinder-CF for PVC	Quantification errors
Grings et al. (Grings *et al.*, 2022)	SPECT kidney, 3D-printed phantoms	W-P RC-based	RC-based using surface area-to-volume (SA:V) ratio	RC, SA:V correlation (R^2)
Jalilifar et al. (Jalilifar *et al.*, 2024)	131I solution in Carlson phantom with varying sphere sizes	W-P- Region-based-PVC	Recovery coefficient estimation through ROI adjustments	Recovery Coefficient (RC)
Azimi et al. (Azimi *et al.*, 2024b)	10 patient 177Lu-PSMA SPECT, dose maps via Monte Carlo with 500M particles.	W-P- Multi-Based	RL iterative deconvolution, PETPVC toolbox, 3D Gaussian kernel	RMSE, MSE, MAE, ME, RE, RAE, SSIM, PSNR

Supplemental Table 6. Summary studies reporting on hybrid and AI-based PVC in SPECT imaging. W-H" represents within hybrid and AI methods.

**Publication**	**Data details**	**Categories of PVC**	**Algorithm/Architecture**	**Evaluation metrics**
Adam et al. (Adam *et al.*, 2022)	3D-printed anthropomorphic head and neck phantom; 131I SPECT/CT imaging.	W-H - Volume-based PVC	Geant4 Monte Carlo RPT dosimetry workflow	Recovery Coefficients (RC), absorbed dose underprediction/overprediction
Yousefi et al. (Yousefi *et al.*, 2023)	99mTc-RBC cardiac SPECT/CT imaging; rest and stress conditions.	W-H - Region-based-PVC	Automatic segmentation/registration via blood pool topology; iYangPVC	IMBV quantification under resting and stress conditions; ΔIMBV (cycle-dependent change in IMBV); Correction effects on IMBV and ΔIMBV
Salvadori et al. (Salvadori *et al.*, 2024)	3D printed kidney geometries on Lu SPECT images; evaluated on GE Discovery NM/CT 870 DR camera.	W-H - Anatomy-based PVC	Two region-based (GTM, Labbé) and five voxel-based methods	Recovery Coefficients (RC), performance and robustness of PVC methods
Gillen et al. (Gillen *et al.*, 2022)	Simulations of 99mTc SPECT imaging; Gaussian PSFs estimated using perturbation.	W-H - Perturbation-based PVC	Single Target Correction using perturbation-estimated PSF	Root mean squared error (RMSE), corrected regional mean values
Xie et al. (Xie *et al.*, 2022)	28 canine studies for cardiac SPECT PVC using Technetium-99m-labeled red blood cells.	W-H - Segmentation-free PVC	Deep-learning-based method with densely-connected dynamic mechanism	SSIM, RMSE, PSNR, and IMBV
Leube et al. (Leube *et al.*, 2024b)	10,000 Monte Carlo simulated SPECT images, phantom validation (3D-printed)	W-H - Deep learning-based PVC	3D U-Net (R2U-Net, AttU-Net variants)	SSIM and NRMSE, Voxel Activity Accuracy (VAA), Activity Deviation
Wang et al. (Wang *et al.*, 2024)	227 PD brain phantoms, 100 clinical 99mTc-TRODAT-1 SPECT; augmented training.	W-H- Deep learning-based PVC	Attention GAN (AttGAN), U-Net, Van-Cittert (VC)	NSR, MAE, SBR, simulation and clinical noise rankings

References

Adam D P, Grudzinski J J, Bormett I, Cox B L, Marsh I R, Bradshaw T J, Harari P M and Bednarz B P 2022 Validation of Monte Carlo 131I radiopharmaceutical dosimetry workflow using a 3D-printed anthropomorphic head and neck phantom *Medical physics* **49** 5491-503

Ahn S, Asma E, Ross S G and Manjeshwar R M *2013 IEEE Nuclear Science Symposium and Medical Imaging Conference (2013 NSS/MIC),2013),* vol. Series*)*: IEEE) pp 1-4

Akerele M, Wadhwa P, Vandenberghe S and Tsoumpas C *2017 IEEE Nuclear Science Symposium and Medical Imaging Conference (NSS/MIC),2017),* vol. Series*)*: IEEE) pp 1-7

Arakawa R, Stenkrona P, Takano A, Nag S, Maior R S and Halldin C 2017 Test-retest reproducibility of [11 C]-l-deprenyl-D 2 binding to MAO-B in the human brain *EJNMMI research* **7** 1-7

Ashrafinia S, Karakatsanis N, Mohy-ud-Din H and Rahmim A *Medical Imaging 2014: Physics of Medical Imaging,2014),* vol. Series 9033*)*: SPIE) pp 625-30

Ashrafinia S, Mohy-ud-Din H, Karakatsanis N A, Jha A K, Casey M E, Kadrmas D J and Rahmim A 2017 Generalized PSF modeling for optimized quantitation in PET imaging *Physics in Medicine & Biology* **62** 5149

Ayubcha C, Raynor W Y, Borja A J, Seraj S M, Rojulpote C, Werner T J, Revheim M-E, Rajapakse C S and Alavi A 2021 Magnetic resonance imaging-based partial volume-corrected 18F-sodium fluoride positron emission tomography in the femoral neck *Nuclear Medicine Communications* **42** 416-20

Azimi M-S, Kamali-Asl A, Ay M-R, Zeraatkar N, Hosseini M-S, Sanaat A, Dadgar H and Arabi H 2024a Deep learning-based partial volume correction in standard and low-dose positron emission tomography-computed tomography imaging *Quantitative Imaging in Medicine and Surgery*

Azimi M-S, Maroufpour S, Hosseini M-S, Zahed A, Alhashim M, Dadgar H, Arabi H and Zaidi H *2024 IEEE Nuclear Science Symposium (NSS), Medical Imaging Conference (MIC) and Room Temperature Semiconductor Detector Conference (RTSD),2024b),* vol. Series*)*: IEEE) pp 1-2

Azimi M, Kamali-Asl A, Ay M-R, Zeraatkar N, Hosseini M-S, Sanaat A and Arabi H 2024c Attention-based deep neural network for partial volume correction in brain 18F-FDG PET imaging *Physica Medica* **119** 103315

Baker S L, Lockhart S N, Maass A and Jagust W J 2017a [P4–052]: PARTIAL VOLUME EFFECTS AND MEDIAL TEMPORAL LOBE TAU QUANTITATION WITH PET *Alzheimer's & Dementia* **13** P1277-P

Baker S L, Maass A and Jagust W J 2017b Considerations and code for partial volume correcting [18F]-AV-1451 tau PET data *Data in brief* **15** 648-57

Bauer C M, Cabral H, Greve D and Killiany R 2013 Differentiating between normal aging, mild cognitive impairment, and Alzheimer's disease with FDG-PET: Effects of normalization region and partial volume correction method *J Alzheimers Dis Parkinsonism* **3**

Baun C, Falch K, Gerke O, Hansen J, Nguyen T, Alavi A, Høilund-Carlsen P-F and Hildebrandt M G 2018 Quantification of FDG-PET/CT with delayed imaging in patients with newly diagnosed recurrent breast cancer *BMC Medical Imaging* **18** 1-10

Belzunce M A, Mehranian A and Reader A J 2018 Enhancement of partial volume correction in MR-guided PET image reconstruction by using MRI voxel sizes *IEEE Transactions on Radiation and Plasma Medical Sciences* **3** 315-26

Bland J, Mehranian A, Belzunce M A, Ellis S, da Costa-Luis C, McGinnity C J, Hammers A and Reader A J 2019 Intercomparison of MR-informed PET image reconstruction methods *Medical physics* **46** 5055-74

Boivin G, Genoud V and Zaidi H 2014 MRI-guided partial volume correction in brain PET imaging: comparison of five algorithms *Frontiers in Biomedical Technologies* **1** 73-81

Bousse A, Pedemonte S, Thomas B A, Erlandsson K, Ourselin S, Arridge S and Hutton B F 2012 Markov random field and Gaussian mixture for segmented MRI-based partial volume correction in PET *Physics in Medicine & Biology* **57** 6681

Bowen S L, Byars L G, Michel C J, Chonde D B and Catana C 2013 Influence of the partial volume correction method on 18F-fluorodeoxyglucose brain kinetic modelling from dynamic PET images reconstructed with resolution model based OSEM *Physics in Medicine & Biology* **58** 7081

Bural G, Torigian D, Basu S, Houseni M, Zhuge Y, Rubello D, Udupa J and Alavi A 2015 Partial volume correction and image segmentation for accurate measurement of standardized uptake value of grey matter in the brain *Nuclear Medicine Communications* **36** 1249-52

Cal-González J, Tsoumpas C, Lassen M, Rasul S, Koller L, Hacker M, Schäfers K and Beyer T 2017 Impact of motion compensation and partial volume correction for 18F-NaF PET/CT imaging of coronary plaque *Physics in Medicine & Biology* **63** 015005

Caldeira L L, da Silva N, Scheins J J, Gaens M E and Shah N J 2015 Effects of regularisation priors and anatomical partial volume correction on dynamic PET data *IEEE Transactions on Nuclear Science* **62** 1725-31

Campanioni S, González-Nóvoa J A, Busto L, Agís-Balboa R C and Veiga C 2023 Data-Driven Phenotyping of Alzheimer's Disease under Epigenetic Conditions Using Partial Volume Correction of PET Studies and Manifold Learning *Biomedicines* **11** 273

Chan C, Liu H, Grobshtein Y, Stacy M R, Sinusas A J and Liu C *2013 IEEE Nuclear Science Symposium and Medical Imaging Conference (2013 NSS/MIC),2013),* vol. Series*)*: IEEE) pp 1-6

Chan C, Liu H, Grobshtein Y, Stacy M R, Sinusas A J and Liu C 2016 Noise suppressed partial volume correction for cardiac SPECT/CT *Medical physics* **43** 5225-39

Chen K T, Salcedo S, Gong K, Chonde D B, Izquierdo-Garcia D, Drzezga A, Rosen B, Qi J, Dickerson B C and Catana C 2019 An efficient approach to perform MR-assisted PET data optimization in simultaneous PET/MR neuroimaging studies *Journal of Nuclear Medicine* **60** 272-8

Coello C, Willoch F, Selnes P, Gjerstad L, Fladby T and Skretting A 2013 Correction of partial volume effect in 18F-FDG PET brain studies using coregistered MR volumes: Voxel based analysis of tracer uptake in the white matter *Neuroimage* **72** 183-92

Corda-D'Incan G, Schnabel J A and Reader A J *2020 IEEE Nuclear Science Symposium and Medical Imaging Conference (NSS/MIC),2020),* vol. Series*)*: IEEE) pp 1-5

Corda-D'Incan G, Schnabel J A, Hammers A and Reader A J 2023 Single-modality supervised joint PET-MR image reconstruction *IEEE Transactions on Radiation and Plasma Medical Sciences*

Corda-D'Incan G, Schnabel J A and Reader A J *2022 IEEE Nuclear Science Symposium and Medical Imaging Conference (NSS/MIC),2022),* vol. Series*)*: IEEE) pp 1-4

Costoya-Sánchez A, Moscoso A, Sobrino T, Ruibal Á, Grothe M J, Schöll M, Silva-Rodríguez J, Aguiar P and Initiative A s D N 2024 Partial volume correction in longitudinal tau PET studies-is it really needed? *NeuroImage* 120537

Cysouw M C, Kramer G M, Hoekstra O S, Frings V, de Langen A J, Smit E F, van den Eertwegh A J, Oprea-Lager D E and Boellaard R 2016 Accuracy and precision of partial-volume correction in oncological PET/CT studies *Journal of Nuclear Medicine* **57** 1642-9

Cysouw M C F, Golla S V S, Frings V, Smit E F, Hoekstra O S, Kramer G M and Boellaard R 2019 Partial-volume correction in dynamic PET-CT: effect on tumor kinetic parameter estimation and validation of simplified metrics *EJNMMI Res* **9** 12

Danesh K, Azimi M, Sharifian P, Karimian A, Arabi H and Zaidi H *2024 IEEE Nuclear Science Symposium (NSS), Medical Imaging Conference (MIC) and Room Temperature Semiconductor Detector Conference (RTSD),2024),* vol. Series*)*: IEEE) pp 1-2

Deidda D, Karakatsanis N A, Robson P M, Tsai Y-J, Efthimiou N, Thielemans K, Fayad Z A, Aykroyd R G and Tsoumpas C 2019 Hybrid PET-MR list-mode kernelized expectation maximization reconstruction *Inverse Problems* **35** 044001

Du Y, Madar I, Stumpf M J, Rong X, Fung G S and Frey E C 2013 Compensation for spill-in and spill-out partial volume effects in cardiac PET imaging *Journal of Nuclear Cardiology* **20** 84-98

Dumouchel T, Thorn S, Kordos M, DaSilva J, Beanlands R S and Robert A d 2012 A three-dimensional model-based partial volume correction strategy for gated cardiac mouse PET imaging *Physics in Medicine & Biology* **57** 4309

Dutta J, El Fakhri G, Zhu X and Li Q *2015 IEEE 12th International Symposium on Biomedical Imaging (ISBI),2015),* vol. Series): IEEE) pp 1423-6

Eldib M, Oesingmann N, Faul D D, Kostakoglu L, Knešaurek K and Fayad Z A 2016 Optimization of yttrium-90 PET for simultaneous PET/MR imaging: A phantom study *Medical Physics* **43** 4768-74

Erlandsson K and Hutton B F 2010 Partial volume correction in SPECT using anatomical information and iterative FBP *Tsinghua Science & Technology* **15** 50-5

Erlandsson K, Thomas B, Dickson J and Hutton B F 2011 Partial Volume correction in SPECT reconstruction with OSEM *Nuclear Instruments and Methods in Physics Research Section A: Accelerators, Spectrometers, Detectors and Associated Equipment* **648** S85-S8

Evans E, Buonincontri G, Izquierdo D, Methner C, Hawkes R C, Ansorge R E, Krieg T, Carpenter T A and Sawiak S J 2015 Combining MRI with PET for partial volume correction improves image-derived input functions in mice *IEEE transactions on nuclear science* **62** 628-33

Farag A, Huang J, Kohan A, Mirshahvalad S A, Dias A B, Fenchel M, Metser U and Veit-Haibach P 2023 Evaluation of MR anatomically-guided PET reconstruction using a convolutional neural network in PSMA patients *Physics in Medicine & Biology* **68** 185014

Fazio P, Schain M, Mrzljak L, Amini N, Nag S, Al-Tawil N, Fitzer-Attas C J, Bronzova J, Landwehrmeyer B and Sampaio C 2017 Patterns of age related changes for phosphodiesterase type-10A in comparison with dopamine D2/3 receptors and sub-cortical volumes in the human basal ganglia: A PET study with 18F-MNI-659 and 11C-raclopride with correction for partial volume effect *Neuroimage* **152** 330-9

Ferraro P M, Campi C, Miceli A, Rolla-Bigliani C, Bauckneht M, Gualco L, Piana M, Marini C, Castellan L and Morbelli S 2022 18F-FDG-PET correlates of aging and disease course in ALS as revealed by distinct PVC approaches *European Journal of Radiology Open* **9** 100394

Filipović M, Barat E, Dautremer T, Comtat C and Stute S 2018 PET reconstruction of the posterior image probability, including multimodal images *IEEE transactions on medical imaging* **38** 1643-54

Finocchiaro D, Berenato S, Grassi E, Bertolini V, Castellani G, Lanconelli N, Versari A, Spezi E, Iori M and Fioroni F 2019 Partial volume effect of SPECT images in PRRT with 177Lu labelled somatostatin analogues: a practical solution *Physica Medica* **57** 153-9

Funck T, Larcher K, Toussaint P-J, Evans A C and Thiel A 2018 APPIAN: automated pipeline for PET image analysis *Frontiers in Neuroinformatics* **12** 64

Funck T, Paquette C, Evans A and Thiel A 2014 Surface-based partial-volume correction for high-resolution PET *Neuroimage* **102** 674-87

Furuta A, Onishi H, Yamaki N, Yada N and Amijima H 2018 Impact of quantitative index derived from 123I-FP-CIT-SPECT on reconstruction with correction methods evaluated using a 3D-striatum digital brain phantom *Radiological Physics and Technology* **11** 294-302

Gallivanone F, Canevari C, Gianolli L, Salvatore C, Della Rosa P, Gilardi M and Castiglioni I 2013 A partial volume effect correction tailored for 18 F-FDG-PET oncological studies *BioMed Research International* **2013**

Gallivanone F, Canevari C, Mapelli P, Picchio M, Gianolli L, Gilardi M C and Castiglioni I 2012 Relationship between 18 F-FDG PET SUV with Partial Volume Correction and Histology in Gastric and Gastro-Oesophageal Cancer

Gallivanone F, Canevari C, Sassi I, Zuber V, Marassi A, Gianolli L, Picchio M, Messa C, Gilardi M and Castiglioni I 2014 Partial volume corrected 18F-FDG PET mean standardized uptake value correlates with prognostic factors in breast cancer *Quarterly Journal of Nuclear Medicine and Molecular Imaging* **58** 424-39

Gao Y, Zhang H, Zhu Y, Bilgel M, Rousset O, Resnick S, Wong D F, Lu L and Rahmim A *2018 IEEE Nuclear Science Symposium and Medical Imaging Conference Proceedings (NSS/MIC),2018),* vol. Series*)*: IEEE) pp 1-4

Gao Y, Zhu Y, Bilgel M, Ashrafinia S, Lu L and Rahmim A 2021 Voxel-based partial volume correction of PET images via subtle MRI guided non-local means regularization *Physica Medica* **89** 129-39

Gargouri S, Mouelhi A, Sayadi M, Labidi S, Mahersi M and Zayed S *2018 4th International Conference on Advanced Technologies for Signal and Image Processing (ATSIP),2018),* vol. Series*)*: IEEE) pp 1-6

Giganti F, De Cobelli F, Canevari C, Orsenigo E, Gallivanone F, Esposito A, Castiglioni I, Ambrosi A, Albarello L and Mazza E 2014 Response to chemotherapy in gastric adenocarcinoma with diffusion-weighted MRI and 18F-FDG-PET/CT: Correlation of apparent diffusion coefficient and partial volume corrected standardized uptake value with histological tumor regression grade *Journal of Magnetic Resonance Imaging* **40** 1147-57

Gillen R, Erlandsson K, Denis-Bacelar A M, Thielemans K, Hutton B F and McQuaid S J 2022 Towards accurate partial volume correction in 99mTc oncology SPECT: perturbation for case-specific resolution estimation *EJNMMI physics* **9** 59

Golla S S, Lubberink M, van Berckel B N, Lammertsma A A and Boellaard R 2017 Partial volume correction of brain PET studies using iterative deconvolution in combination with HYPR denoising *EJNMMI research* **7** 1-12

Grecchi E, O'Doherty J, Veronese M, Tsoumpas C, Cook G J and Turkheimer F E 2015 Multimodal partial-volume correction: application to 18F-fluoride PET/CT bone metastases studies *Journal of Nuclear Medicine* **56** 1408-14

Grecchi E, Veronese M, Bodini B, García-Lorenzo D, Battaglini M, Stankoff B and Turkheimer F E 2017 Multimodal partial volume correction: Application to [11C] PIB PET/MRI myelin imaging in multiple sclerosis *Journal of Cerebral Blood Flow & Metabolism* **37** 3803-17

Greve D N, Salat D H, Bowen S L, Izquierdo-Garcia D, Schultz A P, Catana C, Becker J A, Svarer C, Knudsen G M and Sperling R A 2016 Different partial volume correction methods lead to different conclusions: an 18F-FDG-PET study of aging *Neuroimage* **132** 334-43

Greve D N, Svarer C, Fisher P M, Feng L, Hansen A E, Baare W, Rosen B, Fischl B and Knudsen G M 2014 Cortical surface-based analysis reduces bias and variance in kinetic modeling of brain PET data *Neuroimage* **92** 225-36

Grings A, Jobic C, Kuwert T and Ritt P 2022 The magnitude of the partial volume effect in SPECT imaging of the kidneys: a phantom study *EJNMMI physics* **9** 18

GUO B, HUANG B, LI X, ZHAO J, LI Y, LI S and WU Z 2022 Effect of different β values combined with partial volume effect correction on the semi-quantitative accuracy and image quality of 68Ga-PSMA PET/CT *Chinese Journal of Nuclear Medicine and Molecular Imaging* 401-5

Gutierrez D, Montandon M-L, Assal F, Allaoua M, Ratib O, Lövblad K-O and Zaidi H 2012 Anatomically guided voxel-based partial volume effect correction in brain PET: impact of MRI segmentation *Computerized Medical Imaging and Graphics* **36** 610-9

Hatt M, Le Pogam A, Visvikis D, Pradier O and Le Rest C C 2012 Impact of partial-volume effect correction on the predictive and prognostic value of baseline 18F-FDG PET images in esophageal cancer *Journal of Nuclear Medicine* **53** 12-20

Hatt M, Tixier F, Cheze Le Rest C, Pradier O and Visvikis D 2013a Robustness of intratumour 18 F-FDG PET uptake heterogeneity quantification for therapy response prediction in oesophageal carcinoma *European journal of nuclear medicine and molecular imaging* **40** 1662-71

Hatt M, Tixier F, Le Rest C C and Visvikis D 2013b SU-D-500-04: Impact of Delineation and Partial Volume Effects Correction On PET Uptake Heterogeneity Quantification Through Textural Features Analysis for Therapy Response in Oncology *Medical Physics* **40** 106-

Hatt M, Van Stiphout R, Le Pogam A, Lammering G, Visvikis D and Lambin P 2013c Early prediction of pathological response in locally advanced rectal cancer based on sequential 18F-FDG PET *Acta Oncologica* **52** 619-26

Hellem M N, Vinther-Jensen T, Anderberg L, Budtz-Jørgensen E, Hjermind L E, Larsen V A, Nielsen J E and Law I 2021 Hybrid 2-[18F] FDG PET/MRI in premanifest Huntington's disease gene-expansion carriers: The significance of partial volume correction *Plos one* **16** e0252683

Hofheinz F, Langner J, Petr J, Beuthien-Baumann B, Oehme L, Steinbach J, Kotzerke J and van den Hoff J 2012 A method for model-free partial volume correction in oncological PET *EJNMMI research* **2** 1-12

Hutchcroft W, Wang G, Chen K T, Catana C and Qi J 2016 Anatomically-aided PET reconstruction using the kernel method *Physics in Medicine & Biology* **61** 6668

Ibaraki M, Matsubara K, Shinohara Y, Shidahara M, Sato K, Yamamoto H and Kinoshita T 2022 Brain partial volume correction with point spreading function reconstruction in high-resolution digital PET: comparison with an MR-based method in FDG imaging *Annals of Nuclear Medicine* **36** 717-27

Irace Z, Reilhac A, De Vigo B M, Batatia H and Costes N *2016 IEEE Nuclear Science Symposium, Medical Imaging Conference and Room-Temperature Semiconductor Detector Workshop (NSS/MIC/RTSD),2016),* vol. Series*)*: IEEE) pp 1-3

Ito Y, Fujita N, Hara K, Tada T, Abe S, Katsuno M, Naganawa S and Kato K 2020 New Semi-Quantification Approach for Dopamine Transporter Scan: Quantification of Accumulation by Examining the Approximate Image

Ito Y, Fujita N, Hara K, Tada T, Abe S, Katsuno M, Naganawa S and Kato K 2022 Novel approach to semi-quantification of tracer accumulation in dopamine transporter scan *Journal of Applied Clinical Medical Physics* **23** e13626

Jalilifar M, Sadeghi M, Emami-Ardekani A, Geravand K and Geramifar P 2024 Quantifying partial volume effect in SPECT and planar imaging: optimizing region of interest for activity concentration estimation in different sphere sizes *Nuclear Medicine Communications* 10.1097

Jomaa H, Mabrouk R and Khlifa N *2018 4th International Conference on Advanced Technologies for Signal and Image Processing (ATSIP),2018),* vol. Series*)*: IEEE) pp 1-6

Jones G, O'Keefe G, Veljanovski R, Williams R, Masters C L, Rowe C C and Villemagne V L 2014 IC-P-198: ASSESSING THE ACCURACY OF A CT-BASED APPROACH TO THE PARTIAL VOLUME CORRECTION OF FLUTEMETAMOL-PET IMAGES *Alzheimer's & Dementia* **10** P110-P

Kafkaletos A, Mix M, Sachpazidis I, Carles M, Rühle A, Ruf J, Grosu A L, Nicolay N H and Baltas D 2024a The significance of partial volume effect on the estimation of hypoxic tumour volume with [18F] FMISO PET/CT *EJNMMI physics* **11** 43

Kafkaletos A, Sachpazidis I, Mix M, Carles M, Schäfer H, Rühle A, Nicolay N H, Lazzeroni M, Toma-Dasu I and Grosu A L 2024b Implications of the partial volume effect correction on the spatial quantification of hypoxia based on [18F] FMISO PET/CT data *Physica Medica* **128** 104853

Kaida H, Azuma K, Kawahara A, Takamori S, Akiba J, Fujimoto K, Ishii K and Ishibashi M 2020 Prognostic value of dual-point fluorine-18 fluorodeoxyglucose PET imaging, partial volume correction and glucose transporter-1 expression in resected nonsmall cell lung cancer patients *Nuclear medicine communications* **41** 48-57

Kang S K and Lee J S 2021 Anatomy-guided PET reconstruction using l 1 bowsher prior *Physics in Medicine & Biology* **66** 095010

Kangasmaa T S, Constable C and Sohlberg A O 2021 Quantitative bone SPECT/CT reconstruction utilizing anatomical information *EJNMMI physics* **8** 1-14

Kim E, Shidahara M, Tsoumpas C, McGinnity C J, Kwon J S, Howes O D and Turkheimer F E 2013 Partial volume correction using structural–functional synergistic resolution recovery: comparison with geometric transfer matrix method *Journal of Cerebral Blood Flow & Metabolism* **33** 914-20

Laymon C M, Minhas D S, Royse S K, Aizenstein H J, Cohen A D, Tudorascu D L and Klunk W E 2021 Characterization of point-spread function specification error on Geometric Transfer Matrix partial volume correction in [11C] PiB amyloid imaging *EJNMMI physics* **8** 1-16

Lee S H, Ha S, An H J, Lee J S, Han W, Im S-A, Ryu H S, Kim W H, Chang J M and Cho N 2016 Association between partial-volume corrected SUV max and Oncotype DX recurrence score in early-stage, ER-positive/HER2-negative invasive breast cancer *European journal of nuclear medicine and molecular imaging* **43** 1574-84

Lehnert W, Gregoire M-C, Reilhac A and Meikle S R 2012 Characterisation of partial volume effect and region-based correction in small animal positron emission tomography (PET) of the rat brain *Neuroimage* **60** 2144-57

Leube J, Claeys W, Gustafsson J, Salas-Ramirez M, Lassmann M, Koole M and Tran-Gia J 2024a Position dependence of recovery coefficients in 177Lu-SPECT/CT reconstructions–phantom simulations and measurements *EJNMMI physics* **11** 52

Leube J, Gustafsson J, Lassmann M, Salas-Ramirez M and Tran-Gia J 2024b A Deep-Learning–Based Partial-Volume Correction Method for Quantitative ¹⁷⁷Lu SPECT/CT Imaging *Journal of Nuclear Medicine* jnumed.123.266889

Li X, Zhang M, Huang P, Liu W and Huang Q *BIBE 2019; The Third International Conference on Biological Information and Biomedical Engineering,2019),* vol. Series*)*: VDE) pp 1-5

Liu H, Chan C, Grobshtein Y, Ma T, Liu Y, Wang S, Stacy M R, Sinusas A J and Liu C 2015 Anatomical-based partial volume correction for low-dose dedicated cardiac SPECT/CT *Physics in Medicine & Biology* **60** 6751

Liu Q, Mohy-ud-Din H, Boutagy N E, Jiang M, Ren S, Stendahl J C, Sinusas A J and Liu C 2017 Fully automatic multi-atlas segmentation of CTA for partial volume correction in cardiac SPECT/CT *Physics in Medicine & Biology* **62** 3944

Liu Y, Lu Z, Chen G, Shi K and Mok G S 2024 Partial volume correction for Lu-177-PSMA SPECT *EJNMMI physics* **11** 93

Liu Z, Zhu Y, Fu R and Gao Y *2023 IEEE Nuclear Science Symposium, Medical Imaging Conference and International Symposium on Room-Temperature Semiconductor Detectors (NSS MIC RTSD),2023),* vol. Series*)*: IEEE) pp 1-

López-González F J, Costoya-Sánchez A, Paredes-Pacheco J, Moscoso A, Silva-Rodríguez J, Aguiar P and Initiative A s D N 2022 Impact of spill-in counts from off-target regions on [18F] flortaucipir PET quantification *Neuroimage* **259** 119396

Lu Y, Toyonaga T, Naganawa M, Gallezot J-D, Chen M-K, Mecca A P, van Dyck C H and Carson R E 2021 Partial volume correction analysis for 11C-UCB-J PET studies of Alzheimer's disease *Neuroimage* **238** 118248

Lue K-H, Lin H-H, Kao C-H K, Hsieh H-J, Liu S-H and Chuang K-S 2014 A simple algorithm for subregional striatal uptake analysis with partial volume correction in dopaminergic PET imaging *Annals of nuclear medicine* **28** 33-41

Malpas C B, Saling M M, Velakoulis D, Desmond P, Hicks R J and O'Brien T J 2015 Longitudinal Partial Volume Correction in 2-[18F]-Fluoro-2-Deoxy-D-Glucose Positron Emission Tomography Studies of Alzheimer Disease *Journal of computer assisted tomography* **39** 559

Mandeville J B, Efthimiou N, Weigand-Whittier J, Hardy E, Knudsen G M, Jørgensen L M and Chen Y-C I 2024 Partial volume correction of PET image data using geometric transfer matrices based on uniform B-splines *Physics in Medicine & Biology* **69** 055020

Marquis H, Deidda D, Gillman A, Willowson K, Gholami Y, Hioki T, Eslick E, Thielemans K and Bailey D 2021 Theranostic SPECT reconstruction for improved resolution: application to radionuclide therapy dosimetry *EJNMMI physics* **8** 1-17

Marsh I R, Li C, Grudzinski J, Jeffery J, Longhurst C, Adam D P, Hernandez R, Weichert J P, Harari P M and Bednarz B P 2022 Partial volume correction improves theranostic 124I/131I-CLR1404 tumor dosimetry in xenograft models of head and neck cancer *arXiv preprint arXiv:2202.06423*

Marsh I R, Li C, Grudzinski J, Jeffery J, Longhurst C, Adam D P, Hernandez R, Weichert J P, Harari P M and Bednarz B P 2023 Targeting of Head and Neck Cancer by Radioiodinated CLR1404 in Murine Xenograft Tumor Models with Partial Volume Corrected Theranostic Dosimetry *Cancer Biotherapy & Radiopharmaceuticals* **38** 458-67

Matsubara K, Ibaraki M, Kinoshita T and Initiative A s D N 2022 DeepPVC: prediction of a partial volume-corrected map for brain positron emission tomography studies via a deep convolutional neural network *EJNMMI physics* **9** 50

Matsubara K, Ibaraki M, Shidahara M, Kinoshita T and Initiative A s D N 2020 Iterative framework for image registration and partial volume correction in brain positron emission tomography *Radiological Physics and Technology* **13** 348-57

Matsubara K, Ibaraki M, Shimada H, Ikoma Y, Suhara T, Kinoshita T and Itco H 2016 Impact of spillover from white matter by partial volume effect on quantification of amyloid deposition with [11C] PiB PET *Neuroimage* **143** 316-24

McGinnity C J, Shidahara M, Feldmann M, Keihaninejad S, Barros D A R, Gousias I S, Duncan J S, Brooks D J, Heckemann R A and Turkheimer F E 2013 Quantification of opioid receptor availability following spontaneous epileptic seizures: correction of [11C] diprenorphine PET data for the partial-volume effect *Neuroimage* **79** 72-80

Meechai T, Tepmongkol S and Pluempitiwiriyawej C 2015 Partial-volume effect correction in positron emission tomography brain scan image using super-resolution image reconstruction *The British journal of radiology* **88** 20140119

Mehranian A, Belzunce M A, McGinnity C J, Bustin A, Prieto C, Hammers A and Reader A J 2019 Multi-modal synergistic PET and MR reconstruction using mutually weighted quadratic priors *Magnetic resonance in medicine* **81** 2120-34

Mehranian A, Belzunce M A, McGinnity C J, Prieto C, Hammers A and Reader A J *2017 IEEE Nuclear Science Symposium and Medical Imaging Conference (NSS/MIC),2017a),* vol. Series*)*: IEEE) pp 1-3

Mehranian A, Belzunce M A, Niccolini F, Politis M, Prieto C, Turkheimer F, Hammers A and Reader A J 2017b PET image reconstruction using multi-parametric anato-functional priors *Physics in Medicine & Biology* **62** 5975

Mehranian A, Zaidi H and Reader A J 2017c MR-guided joint reconstruction of activity and attenuation in brain PET-MR *NeuroImage* **162** 276-88

Mertens N, Michiels L, Vanderlinden G, Vandenbulcke M, Lemmens R, Van Laere K and Koole M 2022 Impact of meningeal uptake and partial volume correction techniques on [18F] MK-6240 binding in aMCI patients and healthy controls *Journal of Cerebral Blood Flow & Metabolism* **42** 1236-46

Mikasa S, Akamatsu G, Taniguchi T, Kidera D, Kihara K, Matsuoka K, Amakusa S, Yoshida T and Sasaki M 2015 Standardization of dual time point [18F] 2-Deoxy-2-fluoro-D-glucose-positron emission tomography performed with different positron emission tomography scanners using partial volume correction *Research and Reports in Nuclear Medicine* 1-7

Minhas D S, Price J C, Laymon C M, Becker C R, Klunk W E, Tudorascu D L, Abrahamson E E, Hamilton R L, Kofler J K and Mathis C A 2018 Impact of partial volume correction on the regional correspondence between in vivo [C-11] PiB PET and postmortem measures of Aβ load *NeuroImage: Clinical* **19** 182-9

Mohy-ud-Din H, Boutagy N E, Stendahl J C, Zhuang Z W, Sinusas A J and Liu C 2018 Quantification of intramyocardial blood volume with 99mTc-RBC SPECT-CT imaging: a preclinical study *Journal of Nuclear Cardiology* **25** 2096-111

Morphis M, Van Staden J and du Raan H 2016 O32. Technetium-99m and Gold-198 activity quantification using SPECT/CT Monte Carlo Simulations *Physica Medica: European Journal of Medical Physics* **32** 151

Muellauer J, Willimayer R, Goertzen A, Wanek T, Langer O, Birkfellner W and Kuntner C 2013 18F, 11C and 68Ga in small animal PET imaging *Nuklearmedizin-NuclearMedicine* **52** 250-61

Niesporek S C, Hoffmann S H, Berger M C, Benkhedah N, Kujawa A, Bachert P and Nagel A M 2015 Partial volume correction for in vivo 23Na-MRI data of the human brain *Neuroimage* **112** 353-63

Novosad P and Reader A J 2016 MR-guided dynamic PET reconstruction with the kernel method and spectral temporal basis functions *Physics in Medicine & Biology* **61** 4624

Ortega C, Schaefferkoetter J, Veit-Haibach P, Anconina R, Berlin A, Perlis N and Metser U 2020 18F-DCFPyL PET/CT in patients with subclinical recurrence of prostate cancer: effect of lesion size, smoothing filter, and partial-volume correction on PROMISE criteria *Journal of Nuclear Medicine* **61** 1615-20

Oyama S, Hosoi A, Ibaraki M, McGinnity C J, Matsubara K, Watanuki S, Watabe H, Tashiro M and Shidahara M 2020 Error propagation analysis of seven partial volume correction algorithms for [18 F] THK-5351 brain PET imaging *EJNMMI physics* **7** 1-15

Pandey A K, Sharma P, Pandey M, Aswathi K, Malhotra A and Kumar R 2012 Spreadsheet program for estimating recovery coefficient to get partial volume corrected standardized uptake value in clinical positron emission tomography-computed tomography studies *Indian Journal of Nuclear Medicine: IJNM: The Official Journal of the Society of Nuclear Medicine, India* **27** 89

Paranjpe M D, Chen X, Liu M, Paranjpe I, Leal J P, Wang R, Pomper M G, Wong D F, Benzinger T L and Zhou Y 2019 The effect of ApoE ε4 on longitudinal brain region-specific glucose metabolism in patients with mild cognitive impairment: a FDG-PET study *NeuroImage: Clinical* **22** 101795

Picchio M, Kirienko M, Mapelli P, Dell'Oca I, Villa E, Gallivanone F, Gianolli L, Messa C and Castiglioni I 2014 Predictive value of pre-therapy 18 F-FDG PET/CT for the outcome of 18 F-FDG PET-guided radiotherapy in patients with head and neck cancer *European journal of nuclear medicine and molecular imaging* **41** 21-31

Pourmoghaddas A and Wells R G 2016 Quantitatively accurate activity measurements with a dedicated cardiac SPECT camera: Physical phantom experiments *Medical Physics* **43** 44-51

Ramonaheng K, van Staden J A and du Raan H 2021 The effect of calibration factors and recovery coefficients on 177 Lu SPECT activity quantification accuracy: a Monte Carlo study *EJNMMI physics* **8** 1-23

Raptis E, Parkes L, Anton-Rodriguez J, Carter S, Herholz K and Matthews J 2020 Investigation of the benefit of PVC in high resolution PET as a post and within reconstruction method for FDG brain data

Raptis E, Parkes L M, Anton-Rodriguez J M, Carter S F, Herholz K and Matthews J C *2019 IEEE Nuclear Science Symposium and Medical Imaging Conference (NSS/MIC),2019),* vol. Series*)*: IEEE) pp 1-3

Rausch I, Beitzke D, Li X, Pfaff S, Rasul S, Haug A R, Mayerhoefer M E, Hacker M, Beyer T and Cal-González J 2022 Accuracy of PET quantification in [68Ga] Ga-pentixafor PET/MR imaging of carotid plaques *Journal of Nuclear Cardiology* **29** 492-502

Reeps C, Bundschuh R A, Pellisek J, Herz M, van Marwick S, Schwaiger M, Eckstein H-H, Nekolla S G and Essler M 2013 Quantitative assessment of glucose metabolism in the vessel wall of abdominal aortic aneurysms: correlation with histology and role of partial volume correction *The international journal of cardiovascular imaging* **29** 505-12

Ren C, Ren J, Tian Z, Du Y, Hao Z, Zhang Z, Fang W, Li F, Zhang S and Hsu B 2021 Assessment of cardiac amyloidosis with 99m Tc-pyrophosphate (PYP) quantitative SPECT *EJNMMI physics* **8** 1-16

Salavati A, Borofsky S, Boon-Keng T K, Houshmand S, Khiewvan B, Saboury B, Codreanu I, Torigian D A, Zaidi H and Alavi A 2015 Application of partial volume effect correction and 4D PET in the quantification of FDG avid lung lesions *Molecular imaging and biology* **17** 140-8

Salvadori J, Allegrini O, Opsommer T, Carullo J, Sarrut D, Porot C, Ritzenthaler F, Meyer P and Namer I-J 2024 Anatomy-based correction of kidney PVE on 177 Lu SPECT images *EJNMMI physics* **11** 15

Sanaat A, Shooli H, Böhringer A S, Sadeghi M, Shiri I, Salimi Y, Ginovart N, Garibotto V, Arabi H and Zaidi H 2023 A cycle-consistent adversarial network for brain PET partial volume correction without prior anatomical information *European Journal of Nuclear Medicine and Molecular Imaging* **50** 1881-96

Sanabria Bohórquez S M, Baker S, Manser P T, Tonietto M, Galli C, Wildsmith K R, Zou Y, Kerchner G A, Weimer R and Teng E 2024 Evaluation of partial volume correction and analysis of longitudinal [18F] GTP1 tau PET imaging in Alzheimer's disease using linear mixed-effects models *Frontiers in Neuroimaging* **3** 1355402

Sari H, Erlandsson K, Law I, Larsson H B, Ourselin S, Arridge S, Atkinson D and Hutton B F 2017 Estimation of an image derived input function with MR-defined carotid arteries in FDG-PET human studies using a novel partial volume correction method *Journal of Cerebral Blood Flow & Metabolism* **37** 1398-409

Sasaki K, Maikusa N, Imabayashi E, Yuasa T and Matsuda H 2016 The feasibility of 11C-PIB-PET/CT for amyloid plaque burden: validation of the effectiveness of CT-based partial volume correction *Brain and Behavior* **6** e00532

Sattarivand M, Armstrong J, Szilagyi G M, Kusano M, Poon I and Caldwell C 2013 Region-based partial volume correction techniques for PET Imaging: sinogram implementation and robustness *International Journal of Molecular Imaging* **2013**

Sattarivand M, Kusano M, Poon I and Caldwell C 2012 Symmetric geometric transfer matrix partial volume correction for PET imaging: principle, validation and robustness *Physics in Medicine & Biology* **57** 7101

Schwarz C G, Gunter J L, Lowe V J, Weigand S, Vemuri P, Senjem M L, Petersen R C, Knopman D S and Jack Jr C R 2019 A comparison of partial volume correction techniques for measuring change in serial amyloid PET SUVR *Journal of Alzheimer's Disease* **67** 181-95

Schwarz C G, Senjem M L, Gunter J L, Tosakulwong N, Weigand S D, Kemp B J, Spychalla A J, Vemuri P, Petersen R C and Lowe V J 2017 Optimizing PiB-PET SUVR change-over-time measurement by a large-scale analysis of longitudinal reliability, plausibility, separability, and correlation with MMSE *Neuroimage* **144** 113-27

Scott M R, Edwards N C, Properzi M J, Jacobs H I, Price J C, Lois C, Farrell M E, Hanseeuw B J, Thibault E G and Rentz D M 2024 Contribution of extracerebral tracer retention and partial volume effects to sex differences in Flortaucipir-PET signal *Journal of Cerebral Blood Flow & Metabolism* **44** 131-41

Shah J, Che Y, Sohankar J, Luo J, Li B, Su Y, Wu T and Initiative A s D N 2024 Enhancing Amyloid PET Quantification: MRI-Guided Super-Resolution Using Latent Diffusion Models *Life* **14** 1580

Shidahara M, Thomas B A, Okamura N, Ibaraki M, Matsubara K, Oyama S, Ishikawa Y, Watanuki S, Iwata R and Furumoto S 2017 A comparison of five partial volume correction methods for tau and amyloid PET imaging with [18 F] THK5351 and [11 C] PIB *Annals of nuclear medicine* **31** 563-9

Shigemoto Y, Sone D, Imabayashi E, Maikusa N, Okamura N, Furumoto S, Kudo Y, Ogawa M, Takano H and Yokoi Y 2018 Dissociation of tau deposits and brain atrophy in early Alzheimer’s disease: a combined positron emission tomography/magnetic resonance imaging study *Frontiers in aging neuroscience* **10** 223

Smith C T, Crawford J L, Dang L C, Seaman K L, San Juan M D, Vijay A, Katz D T, Matuskey D, Cowan R L and Morris E D 2019 Partial-volume correction increases estimated dopamine D2-like

receptor binding potential and reduces adult age differences *Journal of Cerebral Blood Flow & Metabolism* **39** 822-33

Song T-A, Chowdhury S R, Kim K, Gong K, El Fakhri G, Li Q and Dutta J *2018 IEEE Nuclear Science Symposium and Medical Imaging Conference Proceedings (NSS/MIC),2018),* vol. Series*)*: IEEE) pp 1-2

Stefano A, Gallivanone F, Messa C, Gilardi M and Gastiglioni I 2014 Metabolic impact of partial volume correction of [18F] FDG PET-CT oncological studies on the assessment of tumor response to treatment *The Quarterly Journal of Nuclear Medicine and Molecular Imaging: Official Publication of the Italian Association of Nuclear Medicine (AIMN)[and] the International Association of Radiopharmacology (IAR),[and] Section of the Society of...* **58** 413-23

Su Y, Blazey T M, Snyder A Z, Raichle M E, Marcus D S, Ances B M, Bateman R J, Cairns N J, Aldea P and Cash L 2015 Partial volume correction in quantitative amyloid imaging *Neuroimage* **107** 55-64

Sudarshan V P, Egan G F, Chen Z and Awate S P 2020 Joint PET-MRI image reconstruction using a patch-based joint-dictionary prior *Medical image analysis* **62** 101669

Sur C, Adamczuk K, Scott D, Kost J, Sampat M, Buckley C, Farrar G, Newton B, Suhy J and Bennacef I 2022 Evaluation of 18F-flutemetamol amyloid PET image analysis parameters on the effect of verubecestat on brain amlyoid load in Alzheimer’s disease *Molecular Imaging and Biology* **24** 862-73

Taghvaei R, Zadeh M Z, Sirous R, Shamchi S P, Raynor W Y, Seraj S M, Moghbel M, Wang S, Werner T J and Zhuang H 2018 Pre-treatment partial-volume-corrected TLG is the best predictor of overall survival in patients with relapsing/refractory non-hodgkin lymphoma following radioimmunotherapy *American Journal of Nuclear Medicine and Molecular Imaging* **8** 407

Tang J and Rahmim A 2014 Anatomy assisted PET image reconstruction incorporating multi-resolution joint entropy *Physics in Medicine & Biology* **60** 31

Teipel S J, Dyrba M, Vergallo A, Lista S, Habert M O, Potier M-C, Lamari F, Dubois B, Hampel H and Grothe M J 2021 Partial Volume correction increases the sensitivity of 18F-florbetapir-positron emission tomography for the detection of early stage amyloidosis *Frontiers in Aging Neuroscience* **13** 748198

Thomas B A, Cuplov V, Bousse A, Mendes A, Thielemans K, Hutton B F and Erlandsson K 2016 PETPVC: a toolbox for performing partial volume correction techniques in positron emission tomography *Physics in Medicine & Biology* **61** 7975

Torigian D A, Dam V, Chen X, Saboury B, Udupa J K, Rashid A, Moghadam-Kia S and Alavi A 2013 In vivo quantification of pulmonary inflammation in relation to emphysema severity via partial volume corrected (18) F-FDG-PET using computer-assisted analysis of diagnostic chest CT *Hellenic journal of nuclear medicine* **16** 12-8

Tran-Gia J, Salas-Ramirez M and Lassmann M 2020 What you see is not what you get: on the accuracy of voxel-based dosimetry in molecular radiotherapy *Journal of Nuclear Medicine* **61** 1178-86

Turco A, Duchenne J, Gheysens O, Nuyts J, Voigt J, Claus P and Vunckx K *2015 IEEE Nuclear Science Symposium and Medical Imaging Conference (NSS/MIC),2015),* vol. Series*)*: IEEE) pp 1-4

Turco A, Gheysens O, Duchenne J, Nuyts J, Rega F, Voigt J-U, Vunckx K and Claus P 2019 Partial volume and motion correction in cardiac PET: First results from an in vs ex vivo comparison using animal datasets *Journal of Nuclear Cardiology* **26** 2034-44

Turco A, Nuyts J, Duchenne J, Gheysens O, Voigt J-U, Claus P and Vunckx K 2020 Analysis of partial volume correction on quantification and regional heterogeneity in cardiac PET *Journal of Nuclear Cardiology* **27** 62-70

Turco A, Nuyts J, Gheysens O, Duchenne J, Voigt J-U, Claus P and Vunckx K 2016 Lesion quantification and detection in myocardial 18F-FDG PET using edge-preserving priors and anatomical information from CT and MRI: A simulation study *EJNMMI physics* **3** 1-32

van Aalst J, Devrome M, Van Weehaeghe D, Rezaei A, Radwan A, Schramm G, Ceccarini J, Sunaert S, Koole M and Van Laere K 2022 Regional glucose metabolic decreases with ageing are associated with microstructural white matter changes: a simultaneous PET/MR study *European Journal of Nuclear Medicine and Molecular Imaging* **49** 664-80

Vunckx K, Atre A, Baete K, Reilhac A, Deroose C M, Van Laere K and Nuyts J 2011 Evaluation of three MRI-based anatomical priors for quantitative PET brain imaging *IEEE transactions on medical imaging* **31** 599-612

Vuohijoki H E, Constable C J and Sohlberg A O 2023 Anatomically guided reconstruction improves lesion quantitation and detectability in bone SPECT/CT *Nuclear Medicine Communications* **44** 330-7

Wang B, Wang H, Huang W, Hung G-U, Hu Z and Mok G *2024 IEEE Nuclear Science Symposium (NSS), Medical Imaging Conference (MIC) and Room Temperature Semiconductor Detector Conference (RTSD),2024),* vol. Series*)*: IEEE) pp 1-2

Wang H and Fei B 2012 An MR image-guided, voxel-based partial volume correction method for PET images *Medical physics* **39** 179-94

Wang Y-W, Wu C-S, Chang C-H, Cheng K-S, Chang Y-K, Huang I-W, Lu C-L and Yao W-J 2015 Partial Volume Correction for Equivocal Retropharyngeal Nodal Metastases of Nasopharyngeal Carcinoma with Fluorodeoxyglucose Positron Emission Tomography–Computed Tomography *Journal of Medical and Biological Engineering* **35** 218-25

Wolters E E, Golla S S, Timmers T, Ossenkoppele R, van der Weijden C W, Scheltens P, Schwarte L, Schuit R C, Windhorst A D and Barkhof F 2018 A novel partial volume correction method for accurate quantification of [18 F] flortaucipir in the hippocampus *EJNMMI research* **8** 1-5

Wu J, Liu H, Hashemi Zonouz T, Sandoval V M, Mohy-ud-Din H, Lampert R J, Sinusas A J, Liu C and Liu Y H 2017 A blind deconvolution method incorporated with anatomical-based filtering for partial volume correction: Validations with 123I-mIBG cardiac SPECT/CT *Medical physics* **44** 6435-46

Wu Z, Guo B, Huang B, Hao X, Wu P, Zhao B, Qin Z, Xie J and Li S 2021 Phantom and clinical assessment of small pulmonary nodules using Q. Clear reconstruction on a silicon-photomultiplier-based time-of-flight PET/CT system *Scientific reports* **11** 10328

Xie H, Liu Z, Shi L, Greco K, Chen X, Zhou B, Feher A, Stendahl J C, Boutagy N and Kyriakides T C 2022 Segmentation-free PVC for cardiac SPECT using a densely-connected multi-dimensional dynamic network *IEEE Transactions on Medical Imaging*

Xu Z, Bagci U, Gao M and Mollura D J *2015 IEEE 12th International Symposium on Biomedical Imaging (ISBI),2015),* vol. Series*)*: IEEE) pp 1196-9

Xu Z, Gao M, Papadakis G Z, Luna B, Jain S, Mollura D J and Bagci U 2018 Joint solution for PET image segmentation, denoising, and partial volume correction *Med Image Anal* **46** 229-43

Yan J, Lim J C-S and Townsend D W 2015 MRI-guided brain PET image filtering and partial volume correction *Physics in medicine & biology* **60** 961

Yang J, Hu C, Guo N, Dutta J, Vaina L M, Johnson K A, Sepulcre J, Fakhri G E and Li Q 2017 Partial volume correction for PET quantification and its impact on brain network in Alzheimer's disease *Scientific Reports* **7** 13035

Yin T-K and Chiu N-T *2017 IEEE 14th International Symposium on Biomedical Imaging (ISBI 2017),2017),* vol. Series*)*: IEEE) pp 1074-7

Yin T-K, Lee B-F, Yang Y K and Chiu N-T 2014 Differences of various region-of-interest methods for measuring dopamine transporter availability using-TRODAT-1 SPECT *The Scientific World Journal* **2014**

Yousefi H, Shi L, Soufer A, Tsatkin V, Bruni W, Avendano R, Greco K, McMahon D, Thorn S and Miller E 2023 Quantification of intramyocardial blood volume using 99mTc-RBC SPECT/CT: a pilot human study *Journal of Nuclear Cardiology* **30** 292-7

Zadeh M Z, Asadollahi S, Kaghazchi F, Raynor W Y, Seraj S M, Werner T J, Seierstad T, Korostoff J, Swisher-McClure S and Alavi A 2022 Prognostic significance of conventional and

volumetric PET parameters with and without partial volume correction in the assessment of head and neck squamous cell carcinoma *Nuclear Medicine Communications* **43** 800-6

Zhao J, Song Y, Liu Q, Chen S and Chen J-C 2023 Optimization of the Algorithm for the Implementation of Point Spread Function in the 3D-OSEM Reconstruction Algorithm Based on the List-Mode Micro PET Data *Electronics* **12** 1309

Zhao Q, Liu M and Zhou Y 2019 Quantitative 18F-AV1451 brain tau PET imaging in cognitively normal older adults, mild cognitive impairment, and Alzheimer's disease patients *Frontiers in neurology* **10** 425111

Zhu Y, Bilgel M, Gao Y, Rousset O G, Resnick S M, Wong D F and Rahmim A 2021 Deconvolution-based partial volume correction of PET images with parallel level set regularization *Physics in Medicine & Biology* **66** 145003

Zhu Y, Gao Y and Rahmim A *2019 IEEE Nuclear Science Symposium and Medical Imaging Conference (NSS/MIC),2019),* vol. Series*)*: IEEE) pp 1-3
